# *N*-Formylsaccharin: A Sweet(able) Formylating Agent in Mechanochemistry

**DOI:** 10.3390/molecules27175450

**Published:** 2022-08-25

**Authors:** Federico Cuccu, Francesco Basoccu, Claudia Fattuoni, Andrea Porcheddu

**Affiliations:** Department of Chemical and Geological Sciences, University of Cagliari, 09124 Monserrato, Italy

**Keywords:** formamides, green chemistry, ball milling, mechanochemistry, saccharin

## Abstract

The acylation of amines has always attracted a deep interest as a synthetic route due to its high versatility in organic chemistry and biochemical processes. The purpose of this article is to present a mechanochemical acylation procedure based on the use of acyl-saccharin derivatives, namely *N*-formylsaccharin, *N*-acetylsaccharin, and *N*-propionylsaccharin. This protocol furnishes a valuable solvent-free alternative to the existing processes and aims to be highly beneficial in multi-step procedures due to its rapid and user-friendly workup.

## 1. Introduction

**Figure 1:**
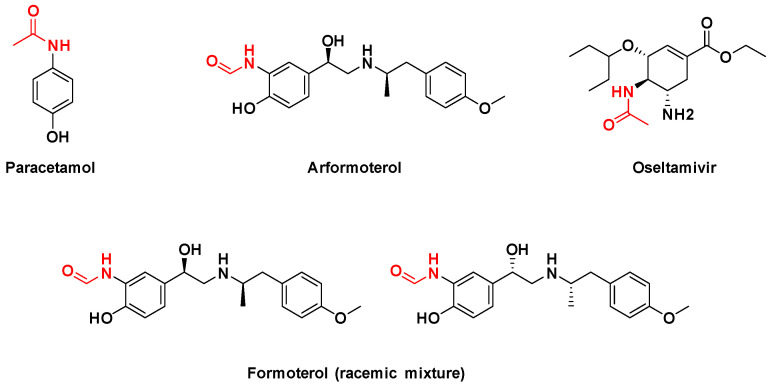
Some examples of acetylated and formylated commercially available drugs.

The derivatization of heteroatoms has recently gained a high degree of interest in the organic and pharmaceutical fields. Therefore, converting the starting material into a new molecule is crucial to emphasize the introduction of valuable functional groups such as pharmaceutical moieties as much as possible. Regarding this, it was found that the attachment of a formyl or acetyl group often confers biological or pharmacological properties, as they can create a more active drug or what could be termed as a pro-drug [1,2]. Several medicines with different therapeutical effects can be numbered among these two classes: paracetamol and other NSAIDs [3] and the antiviral compound Oseltamivir [4] are prominent representatives in the case of the *N*-acetyl group. The *N*-formylated compounds, on the other hand, have been less explored, probably because of their more challenging preparation [5]. However sporadic a chemical structure, some noteworthy drugs are available in the market, namely Formoterol [6] and Arformoterol [7], for pulmonary diseases and Orlistat for the treatment of obesity [8] (Figure 1).

Nonetheless, the utility of these formylated compounds is considerably beyond pure pharmaceutical use. Remarkably, different reactions involve the use of formamides as starting materials, among which the synthesis of isocyanides represents one of the most important topics in recent years [9,10,11,12,13,14]. In this regard, our team’s recent publication has depicted an efficient mechanochemical synthesis of isocyanides starting from formamides [15], again proving the undeniable relevance of mechanochemistry in the panorama of organic synthesis [16,17]. Furthermore, previous studies have demonstrated the potential utility of this class of compounds for successfully synthesizing amidines [18], symmetrical and not symmetrical ureas [19,20], isocyanates [21], and heterocycles [22,23].

Whilst acetylation is a general and effortless procedure to deal with; some operational issues instantly emerge when facing a formylating process. The primary problem lies in the required troublesome conditions: for instance, the employment of gaseous CO_2_ under reductive conditions [24]. Other methodologies involve the utilization of an acidic media, whose acid source could be represented by SiO_2_ [25] or ZrO_2_ [26] as Lewis acids and sulfuric acid [27] or formic acid [28] as Brønsted acid. Similarly, microwave reactions, although a quite performing method, involve ethyl formate at high temperatures (around 100 °C) [29]. Other pathways may involve small molecules such as thiamine hydrochloride in combination with formic acid [30]. Finally, some other formylating agents have been recently proposed, such as DMF [31], sodium formate [32], and CDMT [33].

Analyzing the literature, we foresaw a significant interest in developing better formylating/acylating agents. Taking inspiration from a recent paper by Cossy and co-workers, we chose *N*-formylsaccharin as a suitable solid reagent for running formylation reactions in a ball mill [34]. In addition, *N*-formylsaccharin is also an inexpensive, environmentally friendly reagent and a promising candidate for the large-scale preparation of (form)amides. Furthermore, due to the outstanding relevance of this topic, we decided to expand the scope to the *N*-acylation reactions by examining other *N*-acylsaccharin compounds—namely the *N*-acetyl and *N*-propionyl derivatives. The considerable interest in such products lies in the possibility of accessing many different moieties, laying the groundwork for synthesizing anti-inflammatory drugs and analgesics, such as paracetamol-like compounds [35] and fentanyl derivatives [36].

In many organic processes, the solvent plays a crucial role in the overall performance. It is the major reaction component [37], representing a high economic cost for the industry and a relevant global issue for public health [38,39]. Unfortunately, solvent removal from a process is not a trivial task as it often seriously affects the reaction outcome [40]. Mechanochemistry [41], which IUPAC recently recognized as one of the ten chemical innovations that will change the world [42], has seriously contributed to overcoming these issues by greening many classical solvent-based procedures [43,44,45,46,47,48,49]. In addition, this technology has paved the way for preparing new compounds that are often challenging to synthesize due to solubility concerns [50,51].

Given these considerations, in this article, we have directed our attention to the solventless synthesis of primary and secondary formamides by exploiting the potential of mechanical forces [52,53] as the energy source and the reactivity of a green formylating agent. Moreover, we developed a mechanochemical workup, which allowed us to minimize the amount of an environmentally benign solvent needed for the sole recovery of the final product.

## 2. Results and Discussion

**Scheme 1:**
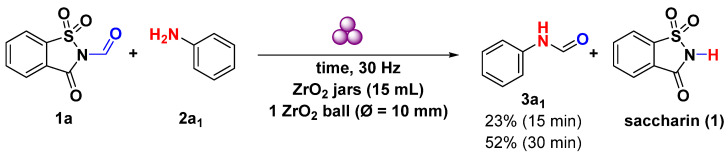
General Scheme of the reaction.

At first, we explored the mechanochemical reaction using experimental conditions close to the classical referral procedure so that a direct comparison with the solvent-based method would be possible. We performed the initial mechanochemical reactions on a mmol scale, milling aniline **2a_1_** (1 mmol) and *N*-formyl saccharin (1 mmol) inside a 15 mL ZrO_2_ vessel equipped with one ball (Ø = 10 mm) of the same material (Scheme 1). We ran the mechanochemical reaction at a frequency of 30 Hz for 15 min. Unfortunately, the reaction gave only a 23% yield of compound **3a_1_**, whereas, when the reaction time was doubled (30 min), we obtained up to 52% yield of the desired product. Therefore, we investigated the influence of other parameters to achieve an almost complete conversion and avoid tedious workups. Considering the formation during the formylating process of saccharin, a relatively acidic compound, we first evaluated whether a base’s presence might avoid the supposed acid-base side-by-side reactions of amines.

In this context, we tested the effect of different carbonates to evaluate the cation (Na, K, Cs) influence and whether the anhydrous or moist form could give better results. All the reagents were added simultaneously into the jar and ground for the time shown in Table 1. At the end of the reaction, the crude was triturated and recovered with ethyl acetate. The resulting formamide was quantified by GC-MS. Unluckily, apart from a slight increase in specific cases, none of these bases showed beneficial effects by shortening the reaction time. Furthermore, metal oxides did not bring any advantage as well. Other solid o liquid organic bases such as imidazole, *N*-methylimidazole and potassium *tert*-butylate failed to improve the conversion yields. Moreover, the use of stainless-steel jars or (more) balls of different materials did not dramatically affect the result. Several hypotheses could be depicted in this regard [54], but it goes beyond the scope of the current study.

When we established that the base’s nature was almost irrelevant, we turned our attention to stoichiometry, especially when the yield seemed to increase slightly. Once again, the results were unsatisfactory, even using a starting material/base ratio of 1:3 (Table 1, entries 4–5, 8–9, 12–13, 16–17). Lastly, we tested some Liquid Assisted Grinding (LAG) conditions. The solvents designated for these experiments were THF and CPME, allowing us to compare the solution approach in the former case and to give a greener alternative in the latter. Different η values were tested, ranging from 0.1 µL/mg to 0.5 µL/mg. The results indicated that small amounts of solvent (LAG) did not enhance the reactivity, at least under our experimental conditions.

Gratifyingly, we found the complete conversion of amine **2a_1_** into the desired formamide **3a_1_** as we increased the reaction time up to 60 minutes without adding a base. Adding a stoichiometric amount of non-anhydrous NaHCO_3_ at the end of the reaction, followed by grinding the resulting reaction crude for an additional 10 min, afforded the formamide **3a_1_** in high yields and purities. The poor solubility of the solid saccharin salt in AcOEt is such that it allows the recovery of **3a1** alone [55].

**Scheme 2:**
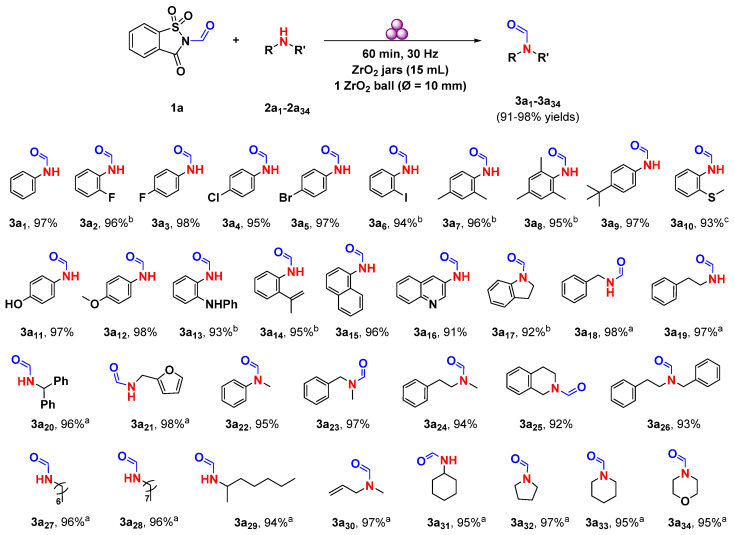
Mechanosynthesis of aryl, alkyl, and heterocyclic formamides. ^a^ 30 min of reaction time; ^b^ 120 min of reaction time; ^c^ 180 min of reaction time. Yields refer to pure isolated compounds.

In the case of aromatic amines and secondary benzylic ones, namely compounds **2a_1_**, **2a_3_**–**2a_5_**, **2a_9_**, **2a_11_**, **2a_12_**, **2a_15_**, **2a_16_**, **2a_22_**–**2a_26_**, the reaction proceeded to a complete conversion within 1 h (Scheme 2).

The results considerably differed as the nucleophilicity of the amine changed. Due to their enhanced nucleophilicity, both primary (aliphatic and benzylic **2a_18_**, **2a_19_**, **2a_21_**, **2a_27_**–**2a_29_**, **2a_31_**) and secondary aliphatic amines **2a_30_**, **2a_32_**–**2a_34_**, only needed 30 min of reaction (Scheme 2). By contrast, the more hindered ortho-substituted anilines **2a_2_**, **2a_6_**, **2a_7_**, **2a_13_**, **2a_14_**, and some heterocycles such as the indoline **2a_17_** required a longer reaction time (2 h) to reach the complete conversion. The *o*-methylthio aniline **2a_10_** required 3 h of milling to be fully converted into its corresponding formamide **3a_10_** (Scheme 2).

**Figure 2:**
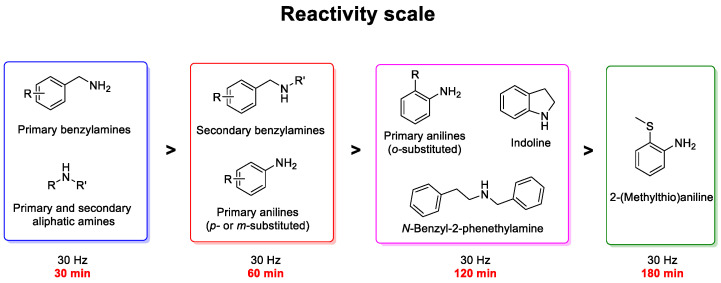
The general reaction trend related to the substrate’s structure.

These results show that the reaction follows a definite trend and a logical behavior intrinsic to each substrate’s chemical characteristics, briefly summarized in Figure 2.

Finally, we calculated the environmental factor (*E*-factor), defined as the waste-to-product ratio for the two formylation methodologies. The results summarized in Table 2 depict a significant improvement, in a green chemistry framework, of the proposed mechanochemical method with respect to the solvent-based procedure (see Supplementary Materials for further details).

Once we explored the formylation reaction, we pursued the idea of extending the methodology to other potential acylating systems. According to the limited literature previously reported on this topic, we moved to the synthesis of *N*-acetyl and *N*-propionyl saccharin-activating agents [56].

**Scheme 3:**
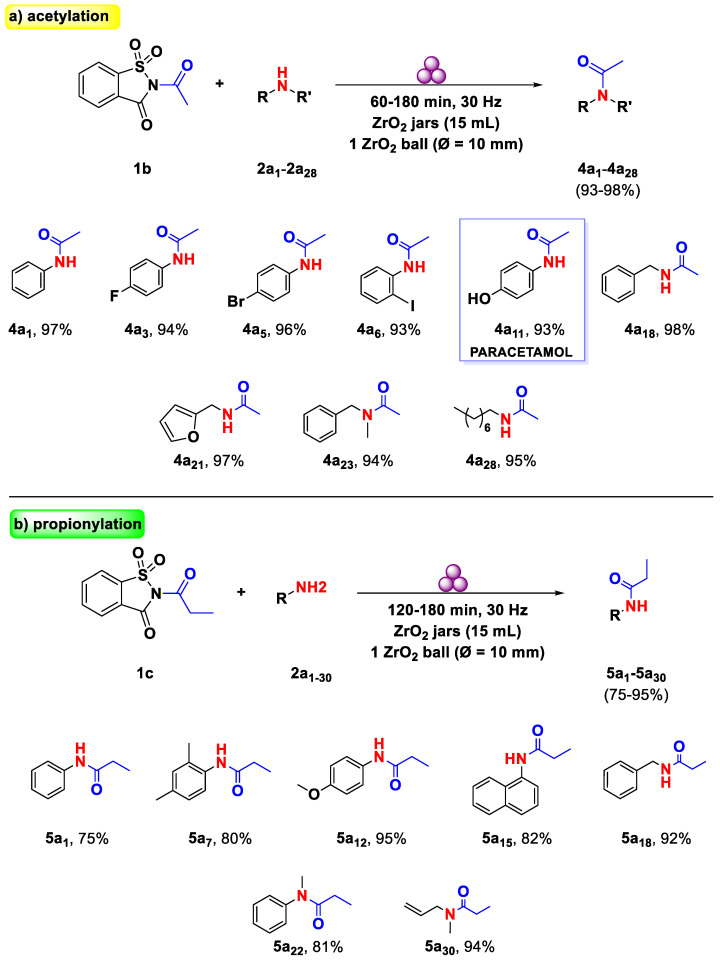
The other acylation scope. (**a**) Acetylation; (**b**) Propionylation.

With an efficient and green amine formylation procedure in hand, we extended this methodology to the mechanosynthesis of acetamides **4a_1_**–**4a_28_** and propionamides **5a_1_**–**5a_30_** from the corresponding amines. The mechanochemical approach allowed a straightforward synthesis of the target amides, avoiding the need for an aqueous acid purification or the requirement of tedious chromatographic techniques. In contrast, analogous solvent-based processes usually require an additional post-synthesis purification stage. The remarkable results can be seen from the data summarized in Scheme 3.

Remarkably, we also obtained an Active Pharmaceutical Ingredient (API) of considerable interest, paracetamol **4a_11_**, an evergreen drug of worldwide use (Scheme 3). The reported methodology results in a straightforward and solvent-free process but also highlights an often-undervalued aspect, namely the purification process. At the end of the reactions, our by-product is saccharin, a non-toxic compound, which can be easily converted into sodium salt through a rapid grinding with moist NaHCO_3_. We used a commercially available, not anhydrous base. Otherwise, two equivalents of water per mmol of NaHCO_3_ should be added to the anhydrous form. This base is strong enough to deprotonate the resulting saccharin but not to hydrolyze our newly formed amide. This was the main problem with the moist form of Na_2_CO_3_, which decomposed about 30% of our product. Therefore, we are firmly convinced that this methodology is suitable for an industrial scale-up and production.

Lastly, aniline was chosen for the propionylation reaction because of the nature of the relative product **5aa** (Scheme 3b). This decision was mainly based on the premise that this amine can represent a perfect building block for fentanyl derivatives, a well-known and widespread drug for treating pain. The model procedure was then successfully extended to an array of amines to validate the methodology (Scheme 3b).

**Scheme 4:**
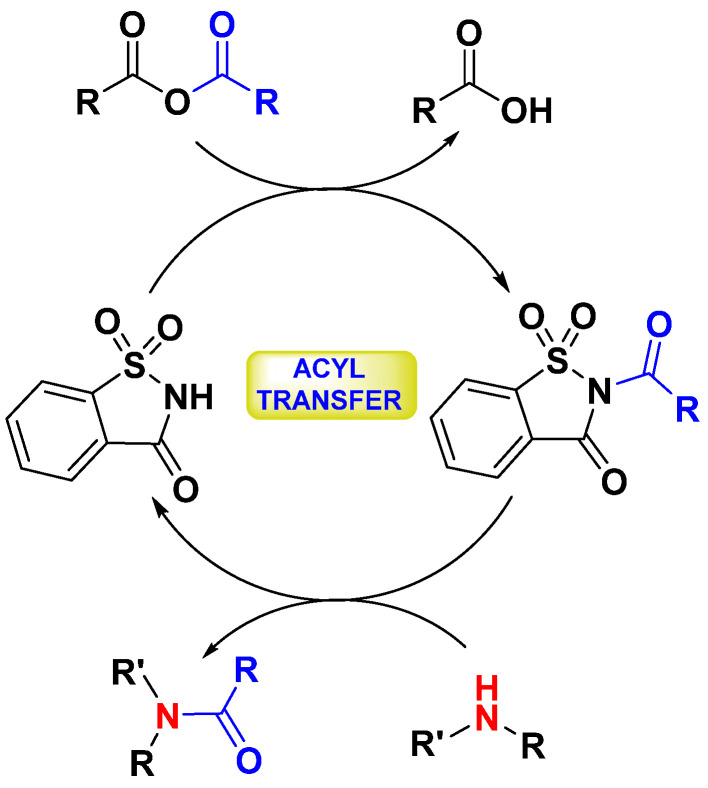
The assumed mechanism for the mechanochemical promoted acyl transfer reaction.

All the syntheses presented are easy to accomplish and proceed with an acyl transfer process. It is a mere transamidation between the *N*-acylsaccharin and the amine. Naturally, the larger the steric hindrance of the acyl group, the lower the yields will be, as reported in the schemes above. The reaction mechanism is briefly described in Scheme 4. It is worth underlining that, at the end of each reaction, the residual saccharinate salt inside the jar can be recovered as a solid to be recycled.

## 3. Materials and Methods

### 3.1. Materials

Commercially available reagents were purchased from Acros (Geel, Belgium), Aldrich (Darmstadt, Germany), Strem Chemicals (Newburyport, MA, USA), Alfa-Aesar (Haverhill, MA, USA), and TCI Europe (Zwijndrecht, Belgium) and used as received. All of the reactions were monitored by thin-layer chromatography (TLC) performed on glass-backed silica gel 60 F254, 0.2 mm plates (Merck, Darmstadt, Germany), and compounds were visualized under UV light (254 nm) or using cerium ammonium molybdate solution with subsequent heating. The eluents were technical grade. The mechanochemical reactions were performed using a FormTech (Hamilton, Canada) FTS-1000 Shaker Mill® apparatus (horizontal vibratory mill). The reagents were milled using a zirconia SmartSnap™ grinding jar (15 mL) equipped with one ball (Ø = 10 mm) of the same material. The ^1^H- and ^13^C-NMR spectra were recorded on a Bruker (Billerica, MA, USA) Avance III HD 600 MHz NMR spectrometer at 298 K. Proton chemical shifts are expressed in parts per million (ppm, δ scale) and are referred to as the residual hydrogen in the solvent (CDCl_3_, 7.27 ppm or DMSO-*d*_6_ 2.54 ppm). Carbon chemical shifts are expressed in parts per million (ppm, δ scale) and are referenced to the carbon resonances of the NMR solvent (CDCl_3_, 77.0 ppm or DMSO-*d*_6_ 39.5 ppm). GC-MS analyses were performed on an Agilent 5977B MS interfaced to the GC 7890B equipped with a DB-5ms column (J & W, New Brighton, UK). Yields refer to pure isolated materials.

### 3.2. General Procedure for N-Formamides Synthesis from Primary and Secondary Amines

A 15 mL ZrO_2_ jar equipped with one ZrO_2_ milling ball (Ø = 10 mm) was filled with amine **2a_1_**–**2a_34_** (1 mmol) and **1a** (1.1 mmol). The vessel was then closed, and the mechanochemical reaction was conducted from 30 to 180 min at a frequency of 30 Hz. At the end of the reaction, an additional 10 min grinding was made with NaHCO_3_ to purify the reaction mixture. The crude was then recovered as a solid in a beaker, dissolved in EtOAc (4 mL) and filtered on paper. The solvent was removed under reduced pressure to afford the pure formamide **3a_1_**–**3a_34_**.

^1^H- and ^13^C-NMR data of compounds are reported in the Supplementary Materials file.

### 3.3. General Procedure for N-Acetamides Pynthesis from Primary and Secondary Amines

A 15 mL ZrO_2_ jar equipped with one ZrO_2_ milling ball (Ø = 10 mm) was filled with amine **2a_1_**, **2a_3_**, **2a_5_**, **2a_6_**, **2a_11_**, **2a_18_**, **2a_21_**, **2a_23_**, or **2a_28_** (1 mmol) and **1b** (1.1 mmol). The vessel was then closed, and the mechanochemical reaction was conducted from 60 to 90 min at a frequency of 30 Hz. At the end of the reaction, an additional 10 min grinding was made with NaHCO_3_ to purify the reaction mixture. The crude was then recovered as a solid in a beaker, dissolved in EtOAc (4 mL) and filtered on paper. The solvent was removed under reduced pressure to afford the pure acetamide **4a_1_**, **4a_3_**, **4a_5_**, **4a_6_**, **4a_11_**, **4a_18_**, **4a_21_**, **4a_23_**, or **4a_28_**.

### 3.4. General Procedure for N-Propionamide Pynthesis from Aniline 

A 15 mL ZrO_2_ jar equipped with one ZrO_2_ milling ball (Ø = 10 mm) was filled with amine **2a_1_**, **2a_7_**, **2a_12_**, **2a_15_**, **2a_18_**, **2a_22_**, or **2a_30_** (1 mmol) and **1c** (1.1 mmol). The vessel was then closed, and the mechanochemical reaction was conducted for 120 min at a frequency of 30 Hz. At the end of the reaction, an additional 10 min grinding was made with NaHCO_3_ to purify the reaction mixture. The crude was then recovered as a solid in a beaker, dissolved in EtOAc (4 mL), and washed with a citric acid solution (5% *p*/*p*). After that, it was dried on Na_2_SO_4_ and filtered on paper. The solvent was removed under reduced pressure to afford the pure propionamide **5a_1_**, **5a_7_**, **5a_12_**, **5a_15_**, **5a_18_**, **5a_22_**, or **5a_30_**.

## 4. Conclusions

A mechanochemical protocol for the formylation of amines using a solid formylated reagent such as *N*-formyl saccharin has been thoroughly described in this work. The reaction is easy to perform and allows to obtain the desired products with good to excellent yields. The purification method is robust, green, and performed as much as possible in the solid phase by limiting the employment of solvent to the sole recovery of the desired compounds. NaHCO_3_ proved to be a green and efficient inorganic salt useful in an easy acid-base purification process. With this method, we could also provide an alternative pathway for synthesizing APIs, such as paracetamol, and valuable building blocks with potential application in the design of fentanyl-like drugs.

## Figures and Tables

**Table 1:** Screening of different bases and ratios.

Entry	Bases	R-NH_2_/Base Ratio	Yields ^a^
1	None	-	52%
2	Li_2_CO_3_ (anhydrous)	1:1	35%
3	Li_2_CO_3_ (wet) ^b^	1:1	32%
4	Li_2_CO_3_ (anhydrous)	1:3	29%
5	Li_2_CO_3_ (wet) ^b^	1:3	29%
6	**K_2_CO_3_ (anhydrous)**	**1:1**	**56%**
7	K_2_CO_3_ (wet) ^b^	1:1	40%
8	K_2_CO_3_ (anhydrous)	1:3	35%
9	K_2_CO_3_ (wet) ^b^	1:3	32%
10	Na_2_CO_3_ (anhydrous)	1:1	53%
11	Na_2_CO_3_ (wet) ^b^	1:1	42%
12	Na_2_CO_3_ (anhydrous)	1:3	39%
13	Na_2_CO_3_ (wet) ^b^	1:3	40%
14	Cs_2_CO_3_ (anhydrous)	1:1	46%
15	Cs_2_CO_3_ (wet) ^b^	1:1	43%
16	Cs_2_CO_3_ (anhydrous)	1:3	45%
17	Cs_2_CO_3_ (wet) ^b^	1:3	45%
18	MgO	1:1	26%
19	CaO	1:1	15%
20	*N*-methylimidazole	1:1	36%
21	Imidazole	1:1	33%
22	*t*-BuOK	1:1	23%

All of the reactions were carried out with the same experimental parameters unless otherwise specified: aniline (1.0 mmol), *N*-formyl saccharin (1.1 mmol), base (1.0 mmol), ZrO_2_ jar (15 mL) and 1 ball (ZrO_2,_ Ø = 10 mm), 30 Hz for 30 min. ^a^ The yields were calculated by GC-MS analysis. ^b^ We used a commercially available, not anhydrous base. Otherwise, two equivalents of H_2_O per mmol of base should be added to the anhydrous form.

**Table 2:** The environmental factor for the synthesis of compound **3a_19_**.

Procedure	*E*-Factor
Solvent-based [32]	>374
Mechanochemistry	26.9

## Data Availability

The data presented in this study are available in Supplementary Materials.

## Supplementary Materials

The following supporting information can be downloaded at: https://www.mdpi.com/article/10.3390/molecules27175450/s1, including General information, Synthesis of compounds, Green chemistry metrics calculations, and Spectra [56,57,58,59,60,61,62,63,64,65,66,67,68,69,70,71,72,73,74,75,76,77,78,79,80,81,82,83,84,85].

## Sample Availability

Samples of all synthesized compound are available from the authors.
