# Supplementary material for: N-Formylsaccharin: A Sweet(able) Formylating Agent in Mechanochemistry
Source: Molecules. 2022 Aug 25;27(17):5450. doi: 10.3390/molecules27175450 (PMC9457594; doi:10.3390/molecules27175450)

1. General information

Commercially available reagents were purchased from Acros, Aldrich, Strem Chemicals, Alfa-Aesar, and TCI Europe and used as received. All reactions were monitored by thin-layer chromatography (TLC) performed on glass-backed silica gel 60 F254, 0.2 mm plates (Merck), and compounds were visualised under UV light (254 nm) or using cerium ammonium molybdate solution with subsequent heating. The eluents were technical grade. Mechanochemical reactions were carried out using a FormTech FTS-1000 Shaker Mill® apparatus (horizontal vibrating mill). The reagents were milled using a zirconia SmartSnap™ grinding jar (15 mL) equipped with balls (ϕ = 10 mm) of the same material. Precisely, the zirconium oxide of the vessels and balls used for all reactions accomplished in the mixer mill is yttrium oxide stabilised (ZrO_2). These parameters were applied if not stated otherwise. ^{1}H and ^{13}C liquid NMR spectra were recorded on a Varian 500 MHz and Bruker Avance III HD 600 MHz NMR spectrometer at 298 K and were calibrated using trimethylsylane (TMS). Proton chemical shifts are expressed in parts per million (ppm, δ scale) and are referred to as the residual hydrogen in the solvent ($CHCl_3$, 7.27 ppm or DMSO 2.54 ppm). The data are represented as follows: chemical shift, multiplicity (s = singlet, d = doublet, t = triplet, q = quartet, m = multiplet and/or multiple resonances, bs = broad singlet, and combination of thereof), coupling constant (J) in Hertz (Hz) and integration. Carbon chemical shifts are expressed in parts per million (ppm, δ scale) and are referenced to the carbon resonances of the NMR solvent ($CDCl_3$, δ 77.0 ppm or δ DMSO-d_6 δ 39.5 ppm). Deuterated NMR solvents were obtained from Aldrich. The samples were analysed using an Agilent 5977B MS interfaced to the GC 7890B equipped with a DB-5ms column (J & W), injector temperature at 230 °C, detector temperature at 280 °C, helium carrier gas flow rate of 1 ml/min. The GC oven temperature program was 60 °C initial temperature with 4 min hold time and ramping at 15°C/min to a final temperature of 270°C with 7 min hold time. Then, 1 µL of each sample was injected in split (1:20) mode. After a solvent delay of three minutes, mass spectra were acquired in full scan mode using 2.28 scans/s with a mass range of 50–500 Amu. Retention times of different compounds were determined by injecting pure compounds under identical conditions. HRMS were recorded on LTQ Orbitrap Elite (Thermofischer) instrument (ESI). All of the experiments were carried out in duplicate to ensure the reproducibility of the experimental data. Yields refer to pure isolated materials. Infrared spectra were recorded on an FT–IR spectrophotometer. All of the experiments were carried out in duplicate to ensure reproducibility of the experimental data. Yields refer to pure isolated materials.

2. Compound 1b and 1c synthesis

General procedure **A** for *N*-acetylsaccharin synthesis from **1**

A 10 mL microwave vessel equipped with one stirring magnet was filled with saccharin **1** (1.0 mmol) and acetic anhydride (1.0 mL, 10.5 eq.). The vessel was then closed, and the reaction was conducted for 240 min at 100 °C and 200 W (power max). At the end of the reaction, the solid was washed with a saturated aqueous $KHCO_3$ solution and filtered on paper to purify the reaction mixture. Lastly, the white solid was let air dry to afford the pure compound **1b**.

General procedure **B** for *N*-propionylsaccharin synthesis from **1**

A 10 mL microwave vessel equipped with one stirring magnet was filled with saccharin **1** (1.0 mmol) and propionic anhydride (1.5 mL, 11.8 eq.). The vessel was then closed, and the reaction was conducted for 300 min at 100 °C and 200 W (power max). At the end of the reaction, the solid was washed with a saturated aqueous $KHCO_3$ solution and filtered on paper to purify the reaction mixture. Lastly, the white solid was let air dry to afford the pure compound **1c**.

2-Acetylbenzo[d]isothiazol-3(2H)-one 1,1-dioxide (**1b)**

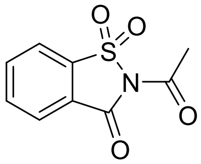

The title compound was synthesized according to the general procedure **A**. **1** (183.2 mg, 1.0 mmol) and acetic anhydride (1.0 mL, 10.5 mmol) were used, to afford **1b** as a white solid (202.7 mg, 0.9 mmol, 90%).

^{1}H NMR (600 MHz, DMSO-*d*$_6$, mixture of rotamers: 50/50) δ = 8.34 – 8.21 (m, 1H), 8.22 – 7.47 (m, 1H), 8.09 – 7.82 (m, 2H), 2.62 – 1.91 (m, 3H).

^{13}C NMR (151 MHz, DMSO-*d*$_6$, mixture of rotamers) δ = 172.0, 168.7, 157.3, 137.2, 135.7, 127.0, 126.1, 124.7, 124.5, 121.5, 120.8, 25.8, 21.0.

The spectroscopic data closely match the ones previously reported in the literature. [56]

2-Propionylbenzo[d]isothiazol-3(2H)-one 1,1-dioxide (**1c**)

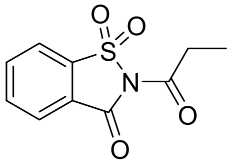

The title compound was synthesized according to the general procedure **B**. **1** (183.2 mg, 1.0 mmol) and propionic anhydride (1.5 mL, 11.8 mmol) were used to afford **1c** as a white solid (208.1 mg, 0.87 mmol, 87%).

^{1}H NMR (600 MHz, DMSO-*d*$_6$, mixture of rotamers: 65/35) δ = 8.33 – 8.20 (m, 1H), 8.14 – 7.89 (m, 1H), 8.09 – 8.04 (m, 1H), 7.98 – 7.85 (m, 1H), 3.07 – 2.19 (m, 2H), 1.13 – 0.97 (m, 3H).

^{13}C NMR (151 MHz, DMSO-*d*$_6$, mixture of rotamers) δ = 175.2, 172.4, 157.3, 137.2, 137.1, 135.6, 126.1, 124.7, 124.6, 121.4, 120.9, 31.0, 26.9, 9.1, 7.5.

The spectroscopic data closely match the ones previously reported in the literature. [56]

3. Mechanochemical synthesis of 3a$_1$-3a$_{34}$, 4a$_1$, 4a$_3$, 4a$_5$, 4a$_6$, 4a$_{11}$, 4a$_{18}$, 4a$_{21}$, 4a$_{23}$, 4a$_{28}$, 5a$_1$, 5a$_7$, 5a$_{12}$, 5a$_{15}$, 5a$_{18}$, 5a$_{22}$, 5a$_{30}$

GENERAL PROCEDURE C FOR *N*-FORMAMIDES SYNTHESIS FROM PRIMARY AND SECONDARY AMINES

A 15 mL ZrO_2 jar equipped with one ZrO_2 milling ball (10 mm diameter) was filled with amine **2a$_1$-2a$_{34}$** (1.0 mmol) and **1a** (1.1 mmol). The vessel was then closed and the mechanochemical reaction was conducted ranging from 30 to 180 min at a frequency of 30 Hz. At the end of the reaction, an additional 10 min grinding was made with 1 equiv. of wet $NaHCO_3$ (it is prepared by adding 2 mmol of H_2O for mmol of dry $NaHCO_3$) for purifying the reaction mixture. The crude was then recovered as a solid in a beaker, dissolved in EtOAc (4 mL) and filtered on paper. Lastly, the solvent was removed under reduced pressure to afford the pure formamide **3a$_1$-3a$_{34}$**.

GENERAL PROCEDURE D FOR *N*-ACETAMIDES SYNTHESIS FROM PRIMARY AND SECONDARY AMINES

A 15 mL ZrO_2 jar equipped with one ZrO_2 milling ball (10 mm diameter) was filled with amine **2a$_1$, 2a$_3$, 2a$_5$, 2a$_6$, 2a$_{11}$, 2a$_{18}$, 2a$_{21}$, 2a$_{23}$, 2a$_{28}$** (1.0 mmol) and **1b** (1.1 mmol). The vessel was then closed and the mechanochemical reaction was conducted ranging from 60 to 180 min at a frequency of 30 Hz. At the end of the reaction, an additional 10 min grinding was made with 1 equiv. of wet $NaHCO_3$ (it is prepared by adding 2 mmol of H_2O for mmol of dry $NaHCO_3$) for purifying the reaction mixture. The crude was then recovered as a solid in a beaker, dissolved in EtOAc (4 mL) and filtered on paper. Lastly, the solvent was removed under reduced pressure to afford the pure acetamide **4a$_1$, 4a$_3$, 4a$_5$, 4a$_6$, 4a$_{11}$, 4a$_{18}$, 4a$_{21}$, 4a$_{23}$, 4a$_{28}$**.

GENERAL PROCEDURE E FOR *N*-PROPIONAMIDE SYNTHESIS FROM PRIMARY AND SECONDARY AMINES

A 15 mL ZrO_2 jar equipped with one ZrO_2 milling ball (10 mm diameter) was filled with amine **2a$_1$, 2a$_7$, 2a$_{12}$, 2a$_{15}$, 2a$_{18}$, 2a$_{22}$, 2a$_{30}$** (1.0 mmol), and **1c** (1.1 mmol). The vessel was then closed, and the mechanochemical reaction was conducted for 120 min at a frequency of 30 Hz. At the end of the reaction, an additional 10 min grinding was made with 1 equiv. of wet $NaHCO_3$ (it is prepared by adding 2 mmol of H_2O for mmol of dry $NaHCO_3$) for purifying the reaction mixture. The crude was then recovered as a solid in a beaker, dissolved in EtOAc (4 mL) and washed with a citric acid solution (5% p/p) dried on Na_2SO_4, when necessary. Lastly, the crude was filtered on paper, and the solvent was removed under reduced pressure to afford the pure propionamide **5a$_1$, 5a$_7$, 5a$_{12}$, 5a$_{15}$, 5a$_{18}$, 5a$_{22}$, 5a$_{30}$**.

N-phenyl formamide (**3a$_1$**)

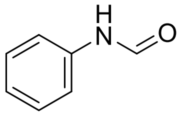

The title compound was synthesized according to the general procedure **C**. **2a$_1$** (93.1 mg, 1.0 mmol) and **1a** (232.3 mg, 1.1 mmol) were used to afford **3a$_1$** as a colourless solid (117.5 mg, 0.97 mmol, 97%).

^{1}H NMR (600 MHz, CDCl$_3$, mixture of rotamers *trans/cis:* 53/47) δ = 8.70 (d, J = 11.3 Hz, 0.53H), 8.38 (s, 0.47H), 8.21 – 7.32 (m, 1H), 7.55 – 7.54 (m, 1H), 7.38 – 7.32 (m, 2H), 7.21 – 7.13 (m, 2H).

^{13}C NMR (151 MHz, CDCl$_3$, mixture of rotamers) δ = 162.7, 159.1, 137.0, 136.8, 129.9, 129.3, 125.5, 125.0, 120.1, 119.0.

The spectroscopic data closely match the ones previously reported in the literature. [57]

N-(2-fluorophenyl) formamide (**3a$_2$**)

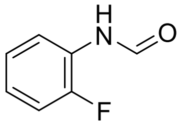

The title compound was synthesized according to the general procedure **C**. **2a$_2$** (111.1 mg, 1.0 mmol) and **1a** (232.3 mg, 1.1 mmol) were used to afford **3a$_2$** as a yellowish liquid (133.5 mg, 0.96 mmol, 96%).

^{1}H NMR (600 MHz, CDCl$_3$, mixture of rotamers *trans/cis:* 68/32) δ = 8.69 (dd, J^1 = 11.3 Hz, J^2 = 1.5 Hz, 0.68H), 8.47 (s, 0.32H), 8.34 – 7.23 (m, 1H), 7.65 – 7.54 (m, 1H), 7.17 – 7.06 (m, 3H).

^{13}C NMR (151 MHz, CDCl$_3$, mixture of rotamers) δ = 162.0, 158.9, 154.1, 153.2, 151.5, 126.2, 126.1, 125.6, 125.5, 125.1, 125.0, 124.9, 124.8, 124.7, 122.3, 119.6, 116.6, 116.5, 115.1, 115.0.

IR (FTIR): 3217, 3074, 1694, 1672, 1618, 1529, 1457, 1413, 1287, 1100, 1037, 748, 698, 635, 519.

HRMS: calculated for $C_7H_6FNO+Na^+$: 162.0331 [*M*+Na]$^+$; found: 162.0329.

N-(4-fluorophenyl) formamide (**3a$_3$**)

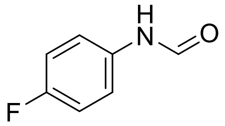

The title compound was synthesized according to the general procedure **C**. **2a$_3$** (111.1 mg, 1.0 mmol) and **1a** (232.3 mg, 1.1 mmol) were used to afford **3a$_3$** as a yellowish solid (136.3 mg, 0.98 mmol, 98%).

^{1}H NMR (600 MHz, CDCl$_3$, mixture of rotamers *trans/cis:* 43/57) δ = 8.57 (d, J = 11.2 Hz, 0.43H), 8.36 – 8.35 (s, 0.57H), 8.17 – 7.37 (m, 1H), 7.52 – 7.49 (dd, 1H), 7.07 – 7.01 (m, 3H).

^{13}C NMR (151 MHz, CDCl$_3$, mixture of rotamers) δ= 162.9, 161.4, 160.6, 159.8, 159.0, 159.0, 133.0, 122.0, 121.9, 121.5, 121.4, 116.8, 116.7, 116.0, 115.9.

The spectroscopic data closely match the ones previously reported in the literature. [58]

N-(4-chlorophenyl) formamide (**3a$_4$**)

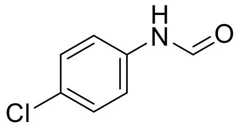

The title compound was synthesized according to the general procedure **C**. **2a$_4$** (127.6 mg, 1.0 mmol) and **1a** (232.3 mg, 1.1 mmol) were used to afford **3a$_4$** as a grey solid (147.8 mg, 0.95 mmol, 95%).

^{1}H NMR (600 MHz, CDCl$_3$, mixture of rotamers *trans/cis:* 41/59) δ= 8.65 (d, J = 11.3 Hz, 0.41H), 8.49 (s, 0.59H), 8.08 (s, 1H), 7.51 – 7.02 (m, 2H), 7.34 – 7.26 (m, 2H).

^{13}C NMR (151 MHz, $CDCl_3$, mixture of rotamers) δ= 162.4, 159.0, 135.5, 135.4, 131.0, 130.1, 130.0, 129.3, 121.3, 120.3.

The spectroscopic data closely match the ones previously reported in the literature. [58]

N-(4-bromophenyl) formamide (**3a$_5$**)

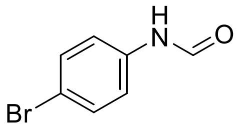

The title compound was synthesized according to the general procedure **C**. **2a$_5$** (172.0 mg, 1.0 mmol) and **1a** (232.3 mg, 1.1 mmol) were used to afford **3a$_5$** as a yellowish liquid (194.0 mg, 0.97 mmol, 97%).

^{1}H NMR (600 MHz, $CDCl_3$, mixture of rotamers *trans/cis:* 40/60) δ = 8.65 (d, *J* = 11.0 Hz, 0.40H), 8.39 – 8.38 (s, 0.60H), 7.85 – 7.22 (m, 1H), 7.49 –6.96 (m, 4H).

^{13}C NMR (151 MHz, $CDCl_3$, mixture of rotamers) δ = 162.1, 158.9, 136.0, 133.0, 132.3, 121.6, 120.6, 118.5, 117.7.

The spectroscopic data closely match the ones previously reported in the literature. [58]

N-(2-iodophenyl) formamide (**3a$_6$**)

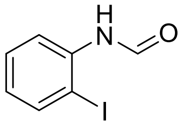

The title compound was synthesized according to the general procedure **C**. **2a$_6$** (219.0 mg, 1.0 mmol) and **1a** (232.3 mg, 1.1 mmol) were used to afford **3a$_6$** as a yellowish liquid (232.2 mg, 0.94 mmol, 94%).

^{1}H NMR (600 MHz, $CDCl_3$, mixture of rotamers *trans/cis:* 38/62) δ = 8.65 (d, *J* = 11.2 Hz, 0.38H), 8.48 (s, 0.62H), 8.28 – 6.86 (m, 2H), 7.85 – 7.78 (m, 1H), 7.59 – 7.51 (m, 1H), 7.37 – 7.34 (m, 1H).

^{13}C NMR (151 MHz, $CDCl_3$, mixture of rotamers) δ = 162.1, 159.1, 159.0, 140.1, 139.1, 137.9, 137.4, 129.8, 129.5, 127.2, 126.5, 122.5, 122.4, 119.5, 90.9, 89.4.

IR (FTIR): 3222, 3017, 2919, 2901, 1655, 1583, 1570, 1521, 1431, 1391, 1279, 1150, 1016,882, 743, 694.

HRMS: calculated for $C_7H_6INO+Na^+$: 269.9392 [*M*+Na]$^+$; found: 269.9392.

The spectroscopic data closely match the ones previously reported in the literature. [59]
N-(2,4-dimethyl) phenyl formamide (**3a$_7$**)

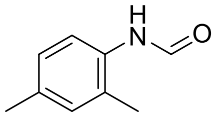

The title compound was synthesized according to the general procedure **C**. **2a$_7$** (121.2 mg, 1.0 mmol) and **1a** (232.3 mg, 1.1 mmol) were used to afford **3a$_7$** as a black solid (143.2 mg, 0.96 mmol, 96%).

^{1}H NMR (600 MHz, $CDCl_3$, mixture of rotamers *trans/cis:* 63/37) δ = 8.47 (d, *J* = 11.4 Hz, 0.63 H), 8.42 (s, 0.37 H), 7.72 – 7.02 (m, 3H) 7.34 – 6.94 (m, 1H), 2.31 – 2.30 (d, 3H), 2.25 (d, 3H).

^{13}C NMR (151 MHz, $CDCl_3$, mixture of rotamers) δ = 163.3, 159.1, 136.2, 135.5, 132.4, 132.0, 131.4, 129.9, 128.9, 127.8, 127.5, 123.4, 121.3, 21.0, 20.9, 17.9, 17.8.

IR (FTIR): 3279, 3021, 2962, 2910, 1675, 1580, 1513, 1434, 1264, 743, 671, 593.

HRMS: calculated for $C_9H_{11}NO+Na^+$: 172.0738 [M+Na]$^+$; found: 172.0735.

N-mesityl formamide (**3a$_8$**)

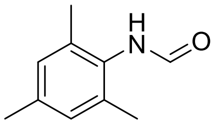

The title compound was synthesized according to the general procedure **C**. **2a$_8$** (135.2 mg, 1.0 mmol) and **1a** (232.3 mg, 1.1 mmol) were used to afford **3a$_8$** as a brown solid (155.0 mg, 0.95 mmol, 95%).

^{1}H NMR (600 MHz, $CDCl_3$, mixture of rotamers *trans*/*cis:* 50/50) δ = 8.40 (s, 0.45 H), 8.05 (d, J = 12.0 Hz, 0.45 H), 6.93 – 6.91 (d, 2H), 6.81 – 6.75 (bs, 1H), 2.34 – 2.22 (m, 9H).

^{13}C NMR (151 MHz, $CDCl_3$, mixture of rotamers) δ = 165.1, 159.6, 158.7, 139.0, 137.8, 137.6, 135.4, 135.2, 133.9, 131.7, 130.5, 129.9, 129.5, 129.4, 129.1, 129.0, 21.1, 21.0, 18.8, 18.7, 18.6.

IR (FTIR): 3282, 3024, 2967, 2900, 1680, 1582, 1513, 1434, 1261, 743, 671, 639.

HRMS: calculated for $C_{10}H_{13}NO+Na^+$: 186.0895 [M+Na]$^+$; found: 186.0894.

The spectroscopic data closely match the ones previously reported in the literature. [60]

N-(4-(*tert*-butyl) phenyl) formamide (**3a$_9$**)

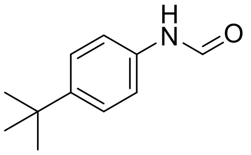

The title compound was synthesized according to the general procedure **C**. **2a$_9$** (149.2 mg, 1.0 mmol) and **1a** (232.3 mg, 1.1 mmol) were used to afford **3a$_9$** as a brownish solid (171.9 mg, 0.97 mmol, 97%).

^{1}H NMR (600 MHz, $CDCl_3$, mixture of rotamers *trans*/*cis:* 54/46) δ= 8.64 (d, J= 11.1 Hz, 0.54H), 8.65 – 8.63 (d, 0.54H), 8.37 – 8.36 (s, 0.46H), 7.71 – 7.18 (m, 1H), 7.46 – 7.35 (m, 3H), 7.03 – 7.01 (m, 1H), 1.32 – 1.31 (d, 9H).

^{13}C NMR (151 MHz, $CDCl_3$, mixture of rotamers) δ= 162.6, 158.9, 148.8, 148.1, 134.3, 134.1, 126.8, 126.1, 119.9, 119.1, 34.7, 34.6, 31.6, 31.5.

The spectroscopic data closely match the ones previously reported in the literature. [61]

N-(2-(methylthio) phenyl) formamide (**3a$_{10}$**)

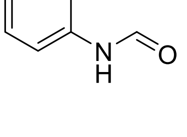

The title compound was synthesized according to the general procedure **C**. **2a$_{10}$** (139.2 mg, 1.0 mmol) and **1a** (232.3 mg, 1.1 mmol) were used to afford **3a$_{10}$** as a black oil (155.5 mg, 0.93 mmol, 93%).

^{1}H NMR (600 MHz, $CDCl_3$, mixture of rotamers *trans/cis:* 34/66) δ = 8.69 (d, J = 11.2 Hz, 0.34H), 8.50 (s, 0.66H), 8.42 – 8.18 (bs, 1H), 8.33 – 7.08 (m, 4H), 2.38 (d, 3H).

^{13}C NMR (151 MHz, $CDCl_3$, mixture of rotamers) δ= 162.1, 159.3, 137.3, 136.2, 132.9, 131.6, 128.8, 128.3, 128.0, 125.8, 125.5, 124.9, 121.2, 118.5, 18.9, 17.7.

IR (FTIR): 3293, 3069, 2994, 2922, 1672, 1582, 1515, 1430, 1296, 743, 671, 635, 533.

HRMS: calculated for C_8H_9NOS+Na^+: 190.0303 [M+Na]$^+$; found: 190.0302.

The spectroscopic data closely match the ones previously reported in the literature. [62]

N-(4-hydroxyphenyl) formamide (**3a11**)

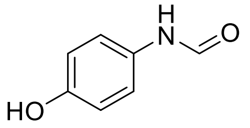

The title compound was synthesized according to the general procedure **C**. **2a11** (109.1 mg, 1.0 mmol) and **1a** (232.3 mg, 1.1 mmol) were used to afford **3a11** as a white solid (133.0 mg, 0.97 mmol, 97%).

^{1}H NMR (600 MHz, DMSO-*d6*, mixture of rotamers *trans/cis:* 35/65) δ= 9.88 – 9.82 (m, 1H), 9.26 – 9.22 (m, 1H), 8.50 (d, J = 11.3 Hz, 0.35H), 8.16 (s, 0.65H), 8.10 – 6.69 (m, 2H), 7.38 – 6.97 (m, 2H).

^{13}C NMR (151 MHz, DMSO-*d6*, mixture of rotamers) δ= 162.4, 159.3, 159.1, 159.0, 122.8, 122.0, 121.2, 120.4.

The spectroscopic data closely match the ones previously reported in the literature. [58]

N-(4-methoxyphenyl) formamide (**3a12**)

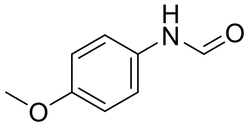

The title compound was synthesized according to the general procedure **C**. **2a12** (123.2 mg, 1.0 mmol) and **1a** (232.3 mg, 1.1 mmol) were used to afford **3a12** as a brownish solid (148.2 mg, 0.98 mmol, 98%).

^{1}H NMR (600 MHz, $CDCl_3$, mixture of rotamers *trans/cis:* 49/51) δ= 8.50 (d, J = 11.2 Hz, 0.49H), 8.33 (s, 0.51H), 7.63 – 7.13 (m, 1H), 7.45 – 7.44 (m, 1H), 7.04 – 7.02 (m, 1H), 6.90 – 6.86 (m, 2H), 3.81 – 3.79 (d, 3H).

^{13}C NMR (151 MHz, $CDCl_3$, mixture of rotamers) δ= 163.0, 158.9, 157.9, 156.9, 130.0, 129.6, 122.0, 121.9, 115.1, 114.4, 55.7, 55.6.

The spectroscopic data closely match the ones previously reported in the literature. [61]
N-(2-(phenylamino) phenyl) formamide (**3a13**)

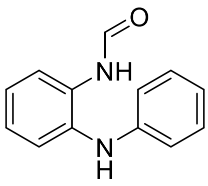

The title compound was synthesized according to the general procedure **C**. **2a13** (184.2 mg, 1.0 mmol) and **1a** (232.3 mg, 1.1 mmol) were used to afford **3a13** as a dark purple oil (197.3 mg, 0.93 mmol, 93%).

^{1}**H NMR (600 MHz, CDCl$_3$, mixture of rotamers)** *δ*= 9.37 (bs, 1H), 8.28 (s, 1H), 7.98 – 7.72 (m, 1H), 7.60 – 7.48 (m, 6H), 7.39 – 7.34 (m, 2H).

^{13}**C NMR (151 MHz, CDCl$_3$, mixture of rotamers)** *δ*= 142.8, 142.2, 136.1, 133.7, 133.4, 130.3, 128.5, 124.3, 124.2, 123.4, 120.3, 110.8.

IR (FTIR): 3300, 3063, 3044, 2897, 1713, 1642, 1579, 1460, 1237, 1180, 1078, 1013, 899.

HRMS: calculated for $C_{11}H_9NO+Na^+$: 194.0582 [*M*+Na]$^+$; found: 194.0586.

N-(2-(prop-1-en-2-yl) phenyl) formamide (**3a$_{14}$**)

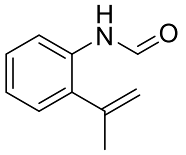

The title compound was synthesized according to the general procedure **C**. **2a$_{14}$** (133.2 mg, 1.0 mmol) and **1a** (232.3 mg, 1.1 mmol) were used to afford **3a$_{14}$** as an orangish solid (153.1 mg, 0.95 mmol, 95%).

^{1}**H NMR (600 MHz, CDCl$_3$, mixture of rotamers *trans/cis:* 50/50)** *δ*= 8.69 (d, *J* = 11.4 Hz, 0.50H), 8.41 – 8.40 (s, 0.50H), 8.33 – 7.09 (m, 4H), 7.62 (bs, 1H), 5.41 – 5.36 (m, 1H), 5.04 – 5.00 (m, 1H), 2.08 – 2.05 (d, 3H).

^{13}**C NMR (151 MHz, CDCl$_3$, mixture of rotamers)** *δ*= 162.4, 162.3, 159.1, 142.6, 142.1, 134.9, 134.7, 134.0, 133.8, 133.5, 133.2, 133.1, 133.0, 132.6, 129.1, 128.7, 128.3, 128.1, 128.0, 127.9, 126.7, 125.2, 125.0, 124.4, 123.8, 122.4, 121.4, 121.0, 120.5, 118.5, 117.6, 117.2, 24.6, 24.2.

IR (FTIR): 3280, 3075, 2972, 2892, 1690, 1668, 1579, 1516, 1445, 1297, 1279, 909, 729.

HRMS: calculated for $C_{10}H_{11}NO+Na^+$: 184.0738 [*M*+Na]$^+$; found: 184.0737.

The spectroscopic data closely match the ones previously reported in the literature. [63]

N-(naphthalen-1-yl) formamide (**3a$_{15}$**)

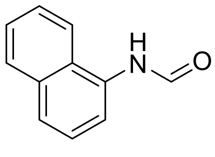

The title compound was synthesized according to the general procedure **C**. **2a$_{15}$** (143.2 mg, 1.0 mmol) and **1a** (232.3 mg, 1.1 mmol) were used to afford **3a$_{15}$** as a pink solid (164.4 mg, 0.96 mmol, 96%).

^{1}**H NMR (600 MHz, CDCl$_3$, mixture of rotamers *trans/cis:* 100/0)** *δ* = 8.61 (d, *J* = 11.3 Hz, 1H), 8.18 (bs, 1H), 8.04 – 7.32 (m, 7H).

^{13}**C NMR (151 MHz, CDCl$_3$, mixture of rotamers)** *δ*= 163.9, 159.6, 134.4, 134.2, 132.1, 129.0, 128.8, 127.9, 127.3, 127.2, 127.0, 126.8, 126.7, 126.4, 126.3, 125.9, 125.7, 121.3, 121.0, 120.4, 119.4.
IR (FTIR): 3221, 2985, 2891, 1654, 1600, 1533, 1506, 1502, 1390, 1270, 1149, 1011, 921, 769.

HRMS: calculated for $C_{11}H_9NO+Na^+$: 194.0582 [*M*+Na]$^+$; found: 194.0586.

N-(quinolin-3-yl) formamide (**3a**$_{16}$)

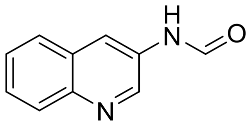

The title compound was synthesized according to the general procedure **C**. **2a**$_{16}$ (144.2 mg, 1.0 mmol) and **1a** (232.3 mg, 1.1 mmol) were used to afford **3a**$_{16}$ as a pale-yellow solid (165.3 mg, 0.96 mmol, 96%).

^{1}H NMR (600 MHz, $CDCl_3$, mixture of rotamers *trans/cis:* 20/80) δ= 10.61 – 10.43 (m, 1H), 8.90 (d, *J* = 10.8 Hz, 0.20H), 8.85 – 8.84 (s, 0.80H), 8.75 – 8.63 (m, 1H), 8.40 – 8.06 (m, 1H), 7.90 – 7.78 (m, 2H), 7.58 – 7.48 (m, 2H).

^{13}C NMR (151 MHz, $CDCl_3$, mixture of rotamers) δ= 163.0, 160.6, 144.7, 144.4, 144.2, 143.7, 132.2, 131.9, 128.7, 128.6, 128.0, 127.9, 127.8, 127.7, 127.3, 127.2, 122.4, 120.0.

IR (FTIR): 3200, 3025, 2877, 1649, 1538, 1493, 1444, 1381, 1238, 1024, 926, 752, 702, 609.

HRMS: calculated for $C_{10}H_9N_2O+H^+$: 173.0715 [*M*+H]$^+$; found: 173.0713.

Indoline-1-carbaldehyde (**3a**$_{17}$)

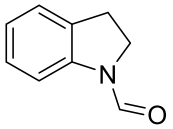

The title compound was synthesized according to the general procedure **C**. **2a**$_{17}$ (119.2 mg, 1.0 mmol) and **1a** (232.3 mg, 1.1 mmol) were used to afford **3a**$_{17}$ as a brownish solid (135.4 mg, 0.92 mmol, 92%).

^{1}H NMR (600 MHz, $CDCl_3$, mixture of rotamers *endo/eso:* 19/81) δ= 8.91 (s, 0.19H), 8.49 (s, 0.81H), 8.09 – 6.99 (m, 4H), 4.09 – 4.02 (m, 2H), 3.18 – 3.11 (m, 2H).

^{13}C NMR (151 MHz, $CDCl_3$, mixture of rotamers) δ= 159.4, 157.6, 141.2, 141.0, 134.8, 134.0, 132.1, 131.9, 127.6, 127.5, 126.0, 125.1, 124.8, 124.6, 124.3, 120.9, 116.6, 109.4, 47.0, 44.6, 27.7, 27.1.

IR (FTIR): 2962, 2931, 2895, 2855, 1640, 1591, 1488, 1413, 1368, 1337, 1287, 1220, 1176, 1033, 743.

HRMS: calculated for $C_9H_9NO+Na^+$: 170.0582 [*M*+Na]$^+$; found: 170.0578.

The spectroscopic data closely match the ones previously reported in the literature. [57]

N-benzyl formamide (**3a**$_{18}$)

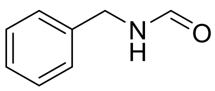

The title compound was synthesized according to the general procedure **C**. **2a**$_{18}$ (107.2 mg, 1.0 mmol) and **1a** (232.3 mg, 1.1 mmol) were used to afford **3a**$_{18}$ as a white solid (132.5 mg, 0.98 mmol, 98%).

^{1}H NMR (600 MHz, $CDCl_3$, mixture of rotamers *trans/cis:* 23/77) δ= 8.29 (s, 0.77H), 8.22 (d, *J* = 12.0 Hz, 0.23H), 7.38 – 7.26 (m, 5H), 5.77 (bs, 1H), 4.51-4.43 (m, 2H).

^{13}C NMR (151 MHz, $CDCl_3$, mixture of rotamers) δ= 164.7, 161.1, 137.7, 129.1, 129.0, 128.1, 128.0, 127.9, 127.1, 45.8, 42.4.

The spectroscopic data closely match the ones previously reported in the literature. [64]

N-(2-phenylethyl) formamide (**3a19**)

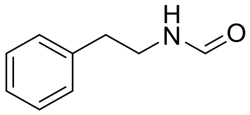

The title compound was synthesized according to the general procedure **C**. **2a19** (121.2 mg, 1.0 mmol) and **1a** (232.3 mg, 1.1 mmol) were used to afford **3a19** as a brownish oil (144.7 mg, 0.97 mmol, 97%).

^{1}H NMR (600 MHz, $CDCl_3$, mixture of rotamers *trans/cis:* 17/83) δ = 7.99 (s, 0.83H), 7.76 (d, J = 12.0 Hz, 0.83H), 7.28 – 7.10 (m, 5H), 6.13 – 6.00 (m, 1H), 3.49 – 3.36 (m, 2H), 2.78 – 2.73 (m, 2H).

^{13}C NMR (151 MHz, $CDCl_3$, mixture of rotamers) δ = 164.6, 161.6, 161.5, 138.6, 138.5, 137.6, 128.7, 128.6, 128.5, 128.4, 126.6, 126.3, 43.1, 39.1, 37.4, 35.3.

The spectroscopic data closely match the ones previously reported in the literature. [65]

N-benzhydrylformamide (**3a20**)

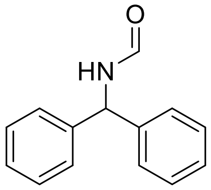

The title compound was synthesized according to the general procedure **C**. **2a20** (183.3 mg, 1.0 mmol) and **1a** (232.3 mg, 1.1 mmol) were used to afford **3a20** as a pale-yellow solid (202.8 mg, 0.96 mmol, 96%).

^{1}H NMR (600 MHz, $CDCl_3$, mixture of rotamers *trans/cis:* 17/83) δ = 8.19 (s, 0.83H), 8.14 (d, J = 12.1 Hz, 0.17H), 7.35 – 7.20 (m, 10H), 6.52 – 6.43 (m, 1H), 6.30 – 5.71 (m, 1H).

^{13}C NMR (151 MHz, $CDCl_3$, mixture of rotamers) δ = 164.5, 160.4, 145.3, 145.0, 141.1, 141.0, 129.1, 128.8, 128.6, 128.5, 128.4, 128.1, 127.8, 127.7, 127.6, 127.5, 127.4, 127.1, 127.0, 60.03, 59.8, 55.8.

IR (FTIR): 3119, 2967, 2913, 1801, 1770, 1721, 1502, 1435, 1368, 1301, 1176, 1042, 805, 586.

HRMS: calculated for $C_{14}H_{13}NO+Na^{+}$: 234.0895 [*M*+Na]$^{+}$; found: 234.0894.

The spectroscopic data closely match the ones previously reported in the literature. [66]

N-(furan-2-ylmethyl) formamide (**3a21**)

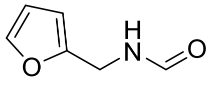

The title compound was synthesized according to the general procedure **C**. **2a21** (97.1 mg, 1.0 mmol) and **1a** (232.3 mg, 1.1 mmol) were used to afford **3a21** as a brownish oil (122.6 mg, 0.98 mmol, 98%).

^{1}H NMR (600 MHz, $CDCl_3$, mixture of rotamers *trans/cis:* 15/85) δ = 8.15 – 8.14 (s, 0.85H), 8.10 (d, J = 11.9 Hz, 0.15H), 7.35 – 7.31 (m, 1H), 6.51 (bs, 1H), 6.31 – 6.20 (m, 2H), 4.43 – 4.32 (m, 2H).

^{13}C NMR (151 MHz, $CDCl_3$, mixture of rotamers) δ = 165.0, 161.3, 150.7, 142.9, 142.4, 110.6, 110.6, 107.7, 107.6, 39.0, 35.1.

The spectroscopic data closely match the ones previously reported in the literature. [67]

N-methyl-*N*-phenylformamide (**3a22**)

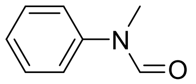

The title compound was synthesized according to the general procedure **C**. **2a22** (107.2 mg, 1.0 mmol) and **1a** (232.3 mg, 1.1 mmol) were used to afford **3a22** as a white solid (128.4 mg, 0.95 mmol, 95%).

^{1}H NMR (600 MHz, $CDCl_3$, mixture of rotamers) δ = 8.48 (s, 0.95H), 8.36 (s, 0.05H), 7.41 (t, *J* = 6Hz, 2H), 7.27 (t, *J* = 6Hz, 1H), 7.18 (d, *J* = 6Hz, 2H), 3.35 – 3.32 (m, 3H).

^{13}C NMR (151 MHz, $CDCl_3$, mixture of rotamers) δ = 162.4,162.2, 142.2, 140.2, 129.7, 129.1, 126.4, 126.3, 123.6, 122.4, 36.9, 32.1.

The spectroscopic data closely match the ones previously reported in the literature. [57]

N-benzyl-*N*-methylformamide (**3a23**)

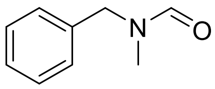

The title compound was synthesized according to the general procedure **C**. **2a23** (121.2 mg, 1.0 mmol) and **1a** (232.3 mg, 1.1 mmol) were used to afford **3a23** as a yellowish oil (144.7 mg, 0.97 mmol, 97%).

^{1}H NMR (600 MHz, $CDCl_3$, mixture of rotamers) δ = 8.27 (s, 0.58H), 8.14 (s, 0.42H), 7.36 – 7.19 (m, 5H), 4.51 – 4.38 (m, 2H), 2.83 – 2.77 (m, 3H).

^{13}C NMR (151 MHz, $CDCl_3$, mixture of rotamers) δ = 162.7, 162.6, 136.0, 135.7, 128.8, 128.6, 128.2, 128.0, 127.6, 127.4, 53.4, 47.7, 34.0, 29.4.

The spectroscopic data closely match the ones previously reported in the literature. [68]

N-methyl-*N*-phenethylformamide (**3a24**)

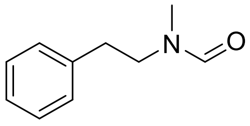

The title compound was synthesized according to the general procedure **C**. **2a24** (135.2 mg, 1.0 mmol) and **1a** (232.3 mg, 1.1 mmol) were used to afford **3a24** as a yellowish oil (153.4 mg, 0.94 mmol, 94%).

^{1}H NMR (600 MHz, $CDCl_3$, mixture of rotamers) δ = 7.98 (s, 0.37H), 7.77 (s, 0.63H), 7.30 – 7.12 (m, 5H), 3.56 – 3.43 (m, 2H), 2.87 – 2.80 (m, 5H).

^{13}C NMR (151 MHz, $CDCl_3$, mixture of rotamers) δ = 162.6, 162.4, 138.5, 137.7, 128.8, 128.7, 128.6, 128.5, 126.7, 126.4, 51.1, 45.9, 34.9, 34.7, 33.1, 29.6.

The spectroscopic data closely match the ones previously reported in the literature. [69]

3,4-dihydroisoquinoline-2(1H)-carbaldehyde (**3a25**)

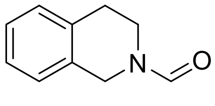

The title compound was synthesized according to the general procedure **C**. **2a25** (133.2 mg, 1.0 mmol) and **1a** (232.3 mg, 1.1 mmol) were used to afford **3a25** as a yellow oil (148.3 mg, 0.92 mmol, 92%).

^{1}H NMR (600 MHz, $CDCl_3$, mixture of rotamers *endo/eso:* 63/37) δ = 8.23 (s, 0.63 H), 8.18 (s, 0.37H), 7.21 – 7.08 (m, 4H), 4.67 –4.52 (m, 2H), 3.78 – 3.62 (m, 2H), 2.90 – 2.85 (m, 2H).

^{13}C NMR (151 MHz, $CDCl_3$, mixture of rotamers) δ = 161.8, 161.2, 134.5, 133.6, 132.3, 131.8, 129.2, 129.0, 127.2, 126.8, 126.7, 126.6, 125.9, 47.4, 43.3, 42.4, 38.1, 29.8, 28.0.

The spectroscopic data closely match the ones previously reported in the literature. [70]

N-benzyl-*N*-phenethylformamide (**3a$_{26}$**)

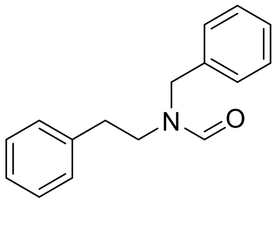

The title compound was synthesized according to the general procedure **C**. **2a$_{26}$** (211.3 mg, 1.0 mmol) and **1a** (232.3 mg, 1.1 mmol) were used to afford **3a$_{26}$** as an orange oil (222.5 mg, 0.93 mmol, 93%).

^{1}H NMR (600 MHz, $CDCl_3$, mixture of rotamers 50/50) δ = 8.26 (s, 0.50H), 7.77 (s, 0.50H), 7.87 – 7.06 (m, 10H), 4.54 – 4.22 (m, 2H), 3.45 – 3.23 (m, 2H), 3.06 – 2.75 (m, 2H).

^{13}C NMR (151 MHz, $CDCl_3$, mixture of rotamers) δ= 166.7, 163.2, 163.1, 142.3, 138.7, 137.8, 136.5, 136.3, 135.9, 133.4, 133.3, 133.2, 131.2, 130.6, 129.4, 129.1, 128.9, 128.8, 128.7, 128.6, 128.5, 128.3, 128.2, 127.8, 127.7, 127.0, 126.8, 126.5, 124.2, 120.6, 52.0, 51.3, 48.5, 48.3, 45.6, 44.0, 35.1, 33.5, 32.3.

IR (FTIR): 3069, 3029, 2931, 1663, 1578, 1457, 1430, 1283, 1149, 1118, 953, 747, 698, 600.

HRMS: calculated for $C_6H_{17}NO+H^+$: 240.1388 [*M*+H]$^+$; found: 240.1389.

The spectroscopic data closely match the ones previously reported in the literature. [71]

N-heptylformamide (**3a$_{27}$**)

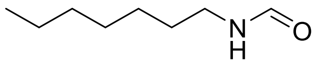

The title compound was synthesized according to the general procedure **C**. **2a$_{27}$** (115.2 mg, 1.0 mmol) and **1a** (232.3 mg, 1.1 mmol) were used to afford **3a$_{27}$** as a yellowish oil (137.5 mg, 0.96 mmol, 96%).

^{1}H NMR (600 MHz, $CDCl_3$, mixture of rotamers *trans/cis:* 21/79) δ = 8.10 (s, 0.79H), 7.96 (d, *J* = 12.0 Hz, 0.21), 6.38 – 6.18 (m, 1H), 3.23 – 3.13 (m, 2H), 1.49 – 1.44 (m, 2H), 1.28 – 1.24 (m, 8H), 0.84 – 0.82 (t, 3H).

^{13}C NMR (151 MHz, $CDCl_3$, mixture of rotamers) δ= 165.0, 161.6, 42.0, 40.1, 38.2, 31.8, 31.7, 31.5, 31.2, 29.5, 28.9, 28.8, 28.7, 26.8, 26.5, 26.4, 22.7, 22.6, 22.5, 14.2, 14.1, 14.0.

The spectroscopic data closely match the ones previously reported in the literature. [72]

N-octyl formamide (**3a$_{28}$**)

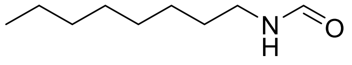

The title compound was synthesized according to the general procedure **C**. **2a$_{28}$** (129.2 mg, 1.0 mmol) and **1a** (232.3 mg, 1.1 mmol) were used to afford **3a$_{28}$** as a yellowish oil (150.9 mg, 0.96 mmol, 96%).

^{1}**H NMR (600 MHz, CDCl$_3$, mixture of rotamers *trans*/*cis:* 27/73)** δ= 8.14 (s, 0.73H), 8.02 (d, *J* = 12.0 Hz, 0.27H), 5.70 (s, 1H), 3.29 – 3.17 (m, 2H), 1.53 – 1.23 (m, 12H), 0.88 – 0.85 (m, 3H).

^{13}**C NMR (151 MHz, CDCl$_3$, mixture of rotamers)** δ = 164.7, 161.3, 41.9, 38.3, 31.9, 31.8, 31.4, 29.6, 29.4, 29.3, 29.2, 29.1, 27.0, 26.0, 22.7, 22.6, 14.2.

The spectroscopic data closely match the ones previously reported in the literature. [73]

(±)-*N*-(heptan-2-yl) formamide (**3a**29)

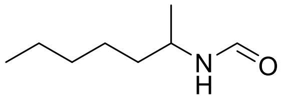

The title compound was synthesized according to the general procedure **C**. **2a**29 (115.2 mg, 1.0 mmol) and **1a** (232.3 mg, 1.1 mmol) were used to afford **3a**29 as a yellowish oil (134.6 mg, 0.94 mmol, 94%).

^{1}**H NMR (600 MHz, CDCl$_3$, mixture of rotamers *trans*/*cis:* 28/72)** δ = 8.13 (s, 0.28H), 8.08 – 8.06 (m, J = 12.1 Hz, 0.72H), 5.30 – 5.29 (m, 1H), 4.08 – 3.47 (m, 1H), 1.46 – 1.15 (m, 11H), 0.90 – 0.87 (m, 3H).

^{13}**C NMR (151 MHz, CDCl$_3$, mixture of rotamers)** δ = 160.6, 48.5, 44.3, 38.0, 37.0, 31.8, 31.6, 25.7, 22.8, 22.7, 22.6, 21.1, 14.1, 14.0.

IR (FTIR): 3271,3062, 2963, 2932, 2861, 1655, 1588, 1530, 1463, 1382, 1275, 1244, 1150, 1123, 819, 699.

N-allyl-*N*-methylformamide (**3a**30)

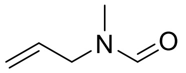

The title compound was synthesized according to the general procedure **C**. **2a**30 (71.1 mg, 1.0 mmol) and **1a** (232.3 mg, 1.1 mmol) were used to afford **3a**30 as a yellowish oil (96.1 mg, 0.97 mmol, 97%).

^{1}**H NMR (600 MHz, CDCl$_3$, mixture of rotamers *trans*/*cis:* 10/90)** δ = 8.06 (s, 0.90H), 7.68 (d, *J* = 12.0 Hz, 0.10H), 5.77 – 5.67 (m, 1H), 5.25 – 5.16 (m, 2H), 3.94 – 3.81 (m, 2H), 2.89 – 2.82 (m, 3H).

^{13}**C NMR (151 MHz, CDCl$_3$, mixture of rotamers)** δ = 162.9, 162.4, 133.0, 132.0, 118.6, 118.2, 52.1, 46.8, 34.1, 29.6.

IR (FTIR): 3100, 2949, 2913, 1761, 1631, 1493, 1457, 1359, 1296, 1238, 1162, 1042, 792, 752, 680.

HRMS: calculated for C_5H_9NO+Na^+: 122.0582 [*M*+Na]$^+$; found: 122.0582.

N-cyclohexylformamide (**3a**31)

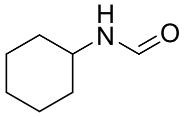

The title compound was synthesized according to the general procedure **C**. **2a**31 (99.2 mg, 1.0 mmol) and **1a** (232.3 mg, 1.1 mmol) were used to afford **3a**31 as a pinkish solid (120.8 mg, 0.95 mmol, 95%).

^{1}**H NMR (600 MHz, CDCl$_3$, mixture of rotamers *trans*/*cis:* 32/68)** δ = 8.11 (d, *J* = 12.1 Hz, 0.32H), 8.09 (s, 0.68H), 5.75 – 5.54 (m, 1H), 3.88 – 3.27 (m, 1H), 1.94 – 1.13 (m, 10H).

^{13}C NMR (151 MHz, $CDCl_3$, mixture of rotamers) δ = 163.6, 160.4, 51.1, 47.2, 34.8, 33.2, 25.6, 25.2, 24.9, 24.8.

The spectroscopic data closely match the ones previously reported in the literature. [68]

Pyrrolidine-1-carbaldehyde (**3a**$_{32}$)

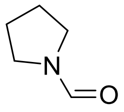

The title compound was synthesized according to the general procedure **C**. **2a**$_{32}$ (71.1 mg, 1.0 mmol) and **1a** (232.3 mg, 1.1 mmol) were used to afford **3a**$_{32}$ as a yellowish oil (96.1 mg, 0.97 mmol, 97%).

^{1}H NMR (600 MHz, $CDCl_3$) δ = 8.24 – 8.08 (m, 1H), 3.49 – 3.40 (m, 4H), 1.92 – 1.88 (m, 4H).

^{13}C NMR (151 MHz, $CDCl_3$) δ = 161.0, 46.2, 43.3, 25.0, 24.4.

The spectroscopic data closely match the ones previously reported in the literature.[57]

Piperidine-1-carbaldehyde (**3a**$_{33}$)

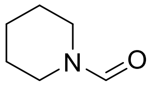

The title compound was synthesized according to the general procedure **C**. **2a**$_{33}$ (85.2 mg, 1.0 mmol) and **1a** (232.3 mg, 1.1 mmol) were used to afford **3a**$_{33}$ as a brownish oil (107.5 mg, 0.95 mmol, 95%).

^{1}H NMR (600 MHz, $CDCl_3$) δ = 7.97 (s, 1H), 3.46 – 3.27 (m, 4H), 1.67 – 1.50 (m, 6H).

^{13}C NMR (151 MHz, $CDCl_3$) δ = 160.9, 46.9, 40.7, 26.7, 25.2, 24.8.

The spectroscopic data closely match the ones previously reported in the literature.[57]

Morpholine-4-carbaldehyde (**3a**$_{34}$)

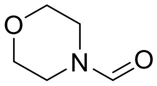

The title compound was synthesized according to the general procedure **C**. **2a**$_{34}$ (87.1 mg, 1.0 mmol) and **1a** (232.3 mg, 1.1 mmol) were used to afford **3a**$_{34}$ as a brownish oil (109.3 mg, 0.95 mmol, 95%).

^{1}H NMR (600 MHz, $CDCl_3$) δ = 8.06 (s, 1H), 3.70 – 3.65 (m, 4H), 3.58 – 3.57 (t, 2H), 3.40 – 3.39 (t, 2H).

^{13}C NMR (151 MHz, $CDCl_3$) δ = 161.0, 67.4, 66.6, 46.0, 40.8.

The spectroscopic data closely match the ones previously reported in the literature.[58]

N-phenylacetamide (**4a**$_{1}$)

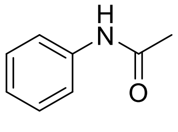

The title compound was synthesized according to the general procedure **D**. **2a**$_{1}$ (93.1 mg, 1.0 mmol) and **1b** (247.7 mg, 1.1 mmol) were used to afford **4a**$_{1}$ as a white solid (131.0 mg, 0.97 mmol, 97%).

^{1}H NMR (600 MHz, $CDCl_3$) δ = 7.57 (bs, 1H), 7.51 – 7.49 (d, 2H), 7.32 – 7.29 (t, 2H), 7.11 – 7.08 (t, 1H), 2.16 (s, 3H).

^{13}C NMR (151 MHz, $CDCl_3$) δ = 168.7, 138.1, 129.1, 124.4, 120.1, 24.7.

The spectroscopic data closely match the ones previously reported in the literature. [74]

N-(4-fluorophenyl) acetamide (**4a**$_3$)

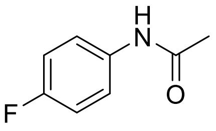

The title compound was synthesized according to the general procedure **D**. **2a**$_3$ (111.1 mg, 1.0 mmol) and **1b** (247.7 mg, 1.1 mmol) were used to afford **4a**$_3$ as a white solid (144.0 mg, 0.94 mmol, 94%).

^{1}H NMR (600 MHz, $CDCl_3$) δ = 7.44 (dt, 3H), 7.00 (t, 2H), 2.16 (s, 3H).

^{13}C NMR (151 MHz, $CDCl_3$) δ = 168.5, 160.3, 158.7, 134.1, 134.0, 122.0, 121.9, 115.8, 115.7, 24.5.

The spectroscopic data closely match the ones previously reported in the literature.[74]

N-(4-bromophenyl) acetamide (**4a**$_5$)

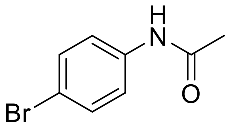

The title compound was synthesized according to the general procedure **D**. **2a**$_5$ (172.1 mg, 1.0 mmol) and **1b** (247.7 mg, 1.1 mmol) were used to afford **4a**$_5$ as a white solid (205.5 mg, 0.96 mmol, 96%).

^{1}H NMR (600 MHz, $CDCl_3$) δ = 8.09 – 7.41 (m, 4H), 7.18 (bs, 1H), 2.17 (s, 3H).

^{13}C NMR (151 MHz, $CDCl_3$) δ = 168.3, 132.1, 121.5, 29.9, 24.8.

The spectroscopic data closely match the ones previously reported in the literature. [75]

N-(2-iodophenyl) acetamide (**4a**$_6$)

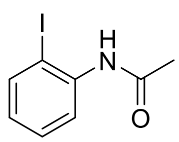

The title compound was synthesized according to the general procedure **D**. **2a**$_6$ (219.0 mg, 1.0 mmol) and **1b** (247.7 mg, 1.1 mmol) were used to afford **4a**$_6$ as a white solid (242.8 mg, 0.93 mmol, 93%).

^{1}H NMR (600 MHz, $CDCl_3$, mixture of rotamers) δ = 8.18 – 7.10 (m, 1H), 7.77 – 7.61 (m, 1H), 7.44 (bs, 1H), 7.34 – 6.44 (m, 1H), 6.85 – 6.72 (m, 1H), 4.09 – 2.23 (s, 3H).

^{13}C NMR (151 MHz, $CDCl_3$, mixture of rotamers) δ = 168.4, 146.9, 139.1, 138.9, 138.3, 129.5, 129.4, 126.1, 122.3, 120.0, 114.8, 90.2, 84.3, 29.8, 24.9.

IR (FTIR): 3364, 2976, 2922, 1765, 1613, 1502, 1439, 1363, 1301, 1265, 1180, 1042, 1002, 752.

The spectroscopic data closely match the ones previously reported in the literature. [76]

N-(4-hydroxyphenyl) acetamide (**4a11**)

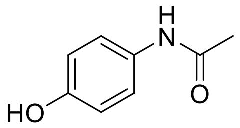

The title compound was synthesized according to the general procedure **D**. **2a11** (109.1 mg, 1.0 mmol) and **1b** (247.7 mg, 1.1 mmol) were used to afford **4a11** as a white solid (140.5 mg, 0.93 mmol, 93%).

^{1}H NMR (600 MHz, DMSO-*d6*) δ = 9.60 (s, 1H), 9.12 (s, 1H), 7.30 – 7.28 (d, 2H), 6.64 – 6.62 (d, 2H), 1.93 (s, 3H).

^{13}C NMR (151 MHz, DMSO-*d6*) δ = 167.5, 153.2, 131.0, 120.8, 115.0, 23.7.

The spectroscopic data closely match the ones previously reported in the literature. [77]

N-benzylacetamide (**4a18**)

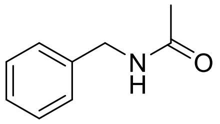

The title compound was synthesized according to the general procedure **D**. **2a18** (107.2 mg, 1.0 mmol) and **1b** (247.7 mg, 1.1 mmol) were used to afford **4a18** as a white solid (146.2 mg, 0.98 mmol, 98%).

^{1}H NMR (600 MHz, CDCl3) δ = 7.35 – 7.32 (m, 2H), 7.29 – 7.26 (m, 3H), 5.80 (bs, 1H), 4.43 – 4.42 (d, 2H), 2.02 (s, 3H).

^{13}C NMR (151 MHz, CDCl3) δ = 170.0, 138.4, 128.9, 128.0, 127.7, 43.9, 23.4.

The spectroscopic data closely match the ones previously reported in the literature. [78]

N-(furan-2-ylmethyl) acetamide (**4a21**)

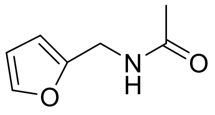

The title compound was synthesized according to the general procedure **D**. **2a21** (97.1 mg, 1.0 mmol) and **1b** (247.7 mg, 1.1 mmol) were used to afford **4a21** as a colourless solid (135.0 mg, 0.97 mmol, 97%).

^{1}H NMR (600 MHz, CDCl3) δ = 7.34 (dd, 1H), 6.31 – 6.30 (dd, 1H), 6.22 – 6.21 (m, 1H), 5.92 (s, 1H), 4.41 (d, 2H), 1.99 (s, 3H).

^{13}C NMR (151 MHz, CDCl3) δ = 170.0, 151.4, 142.3, 110.6, 107.6, 36.7, 23.3.

IR (FTIR): 3275, 3078, 1636, 1551, 1497, 1435, 1368, 1287, 1252, 1149, 1064, 1024, 810, 752, 725.

HRMS: calculated for $C_7H_9NO_2$+H^+: 140.0712 [*M*+H]$^+$; found: 140.0708.

The spectroscopic data closely match the ones previously reported in the literature. [79]

N-benzyl-*N*-methylacetamide (**4a**$_{23}$)

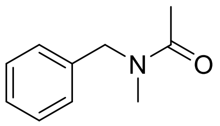

The title compound was synthesized according to the general procedure **D**. **2a**$_{23}$ (121.2 mg, 1.0 mmol) and **1b** (247.7 mg, 1.1 mmol) were used to afford **4a**$_{23}$ as a white solid (153.4 mg, 0.94 mmol, 94%).

^{1}H NMR (600 MHz, CDCl$_3$, mixture of rotamers 55/45) δ = 7.42 – 7.08 (m, 5H), 4.64 – 4.48 (m, 2H), 3.06 – 2.82 (m, 3H), 2.18 (s, 3H).

^{13}C NMR (151 MHz, CDCl$_3$, mixture of rotamers) δ = 171.5, 171.2, 129.1, 128.7, 128.2, 128.0, 127.5, 126.4, 54.4, 50.8, 35.7, 34.0, 21.9, 21.5.

The spectroscopic data closely match the ones previously reported in the literature.[68]

N-octylacetamide (**4a**$_{28}$)

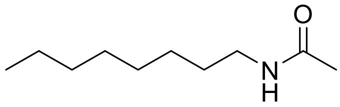

The title compound was synthesized according to the general procedure **D**. **2a**$_{28}$ (129.2 mg, 1.0 mmol) and **1b** (247.7 mg, 1.1 mmol) were used to afford **4a**$_{28}$ as a yellow oil (162.7 mg, 0.95 mmol, 95%).

^{1}H NMR (600 MHz, CDCl$_3$) δ = 5.71 (bs, 1H), 3.21 – 3.18 (m, 2H), 1.95 (s, 3H), 1.49 – 1.44 (m, 2H), 1.30 – 1.23 (m, 10H), 0.86 – 0.84 (m, 3H).

^{13}C NMR (151 MHz, CDCl$_3$) δ = 170.3, 39.8, 31.9, 29.7, 29.4, 29.3, 27.0, 23.4, 22.7, 14.2.

IR (FTIR): 3285, 3084, 2959, 2928, 2856, 1650, 1556, 1467, 1440, 1373, 1293, 1145, 726, 605.

The spectroscopic data closely match the ones previously reported in the literature. [80]

N-phenylpropionamide (**5a**$_1$)

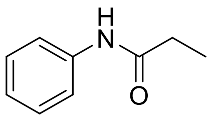

The title compound was synthesized according to the general procedure **E**. **2a**$_1$ (93.1 mg, 1.0 mmol) and **1c** (263.1 mg, 1.1 mmol) were used to afford **5a**$_1$ as a white solid (111.8 mg, 0.75 mmol, 75%).

^{1}H NMR (600 MHz, CDCl$_3$) δ = 8.00 (bs, 1H), 7.52 – 7.51 (d, 2H), 7.28 – 7.25 (t, 2H), 7.08 – 7.05 (t, 1H), 2.37 – 2.34 (q, 2H), 1.21 – 1.18 (t, 3H).

^{13}C NMR (151 MHz, CDCl$_3$) δ = 172.8, 138.2, 128.9, 124.2, 120.2, 30.7, 9.8.

The spectroscopic data closely match the ones previously reported in the literature.[76]

N-(2,4-dimethylphenyl) propionamide (**5a**$_7$)

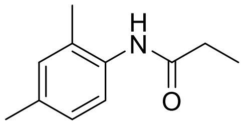

The title compound was synthesized according to the general procedure **E**. **2a**$_7$ (121.2 mg, 1.0 mmol) and **1c** (263.1 mg, 1.1 mmol) were used to afford **5a**$_7$ as a white solid (141.8 mg, 0.80 mmol, 80%).

^{1}H NMR (600 MHz, CDCl$_3$, mixture of rotamers 86/14) δ = 7.57 – 6.98 (m, 4H), 3.08 – 2.38 (m, 2H), 2.28 (s, 3H), 2.20 (s, 3H), 1.28 – 1.24 (m, 3H).

^{13}C NMR (151 MHz, CDCl$_3$, mixture of rotamers) δ = 172.2, 157.8, 136.5, 135.1, 135.0, 133.2, 131.2, 129.7, 127.3, 126.4, 123.8, 121.3, 32.0, 30.6, 21.0, 17.8, 10.1, 7.7.

IR (FTIR): 3271, 2968, 2919, 2861, 1650, 1525, 1431, 1364, 1270, 1212, 815, 699.

The spectroscopic data closely match the ones previously reported in the literature. [81]

N-(4-methoxyphenyl) propionamide (**5a12**)

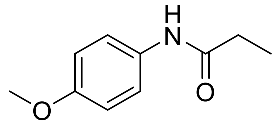

The title compound was synthesized according to the general procedure **E**. **2a12** (123.2 mg, 1.0 mmol) and **1c** (263.1 mg, 1.1 mmol) were used to afford **5a12** as a brownish solid (170.2 mg, 0.95 mmol, 95%).

^{1}H NMR (600 MHz, CDCl$_3$, mixture of rotamers 87/13) δ = 7.78 (bs, 1H), 7.40 – 7.38 (dd, 2H), 6.80 – 6.78 (dd, 2H), 3.75 – 3.74 (d, 3H), 3.06 – 2.30 (m, 2H), 1.26 – 1.17 (m, 1H).

^{13}C NMR (151 MHz, CDCl$_3$, mixture of rotamers) δ = 172.5, 172.2, 156.3, 136.6 135.0, 131.3, 126.3, 122.0, 121.2, 114.1, 55.5, 31.9, 30.4, 9.9, 7.6.

IR (FTIR): 3312, 3008, 2981, 2941, 2843, 1641, 1597, 1538, 1516, 1463, 1248, 1177, 1034, 824, 681, 525.

The spectroscopic data closely match the ones previously reported in the literature.[76]

N-(naphthalen-1-yl) propionamide (**5a15**)

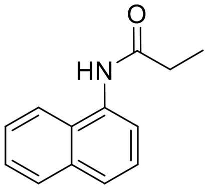

The title compound was synthesized according to the general procedure **E**. **2a15** (143.2 mg, 1.0 mmol) and **1c** (263.1 mg, 1.1 mmol) were used to afford **5a15** as a pink solid (163.4 mg, 0.82 mmol, 82%).

^{1}H NMR (600 MHz, CDCl$_3$) δ = 7.84 – 7.38 (m, 8H), 2.46 (m, 2H), 1.27 (m, 3H).

^{13}C NMR (151 MHz, CDCl$_3$) δ = 173.0, 134.2, 132.5, 128.7, 127.6, 126.2, 126.0, 125.9, 125.7, 121.4, 121.0, 30.5, 10.0.

IR (FTIR): 3263, 3066, 2977, 2936, 2883, 1655, 1538, 1498, 1275, 1248, 1217, 931, 801, 766, 717.

The spectroscopic data closely match the ones previously reported in the literature. [82]

N-benzylpropionamide (**5a18**)

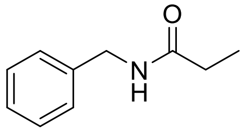

The title compound was synthesized according to the general procedure **E**. **2a18** (107.2 mg, 1.0 mmol) and **1c** (263.1 mg, 1.1 mmol) were used to afford **5a18** as a colourless solid (150.1 mg, 0.92 mmol, 92%).

^{1}H NMR (600 MHz, CDCl$_3$) δ = 7.33 – 7.25 (m, 5H), 6.07 (bs, 1H), 4.43 – 4.42 (d, 2H), 2.28 – 2.24 (q, 2H), 1.19 – 1.16 (t, 3H).

^{13}C NMR (151 MHz, CDCl$_3$) δ = 174.3, 138.3, 128.8, 127.9, 127.6, 43.7, 29.7, 10.0.

The spectroscopic data closely match the ones previously reported in the literature. [83]

N-methyl-*N*-phenylpropionamide (**5a$_{22}$**)

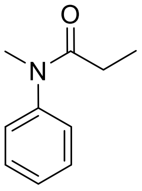

The title compound was synthesized according to the general procedure **E**. **2a$_{22}$** (107.2 mg, 1.0 mmol) and **1c** (263.1 mg, 1.1 mmol) were used to afford **5a$_{22}$** as an orange oil (132.4 mg, 0.81 mmol, 81%).

^{1}H NMR (600 MHz, CDCl$_3$) δ = 7.42 – 7.19 (m, 5H), 3.27 (s, 3H), 2.09 (m, 2H), 1.05 (m, 3H).

^{13}C NMR (151 MHz, CDCl$_3$) δ = 174.1, 173.6, 144.1, 130.1, 129.7, 129.0, 127.7, 127.2, 122.8, 37.3, 27.4, 20.8, 9.6.

IR (FTIR): 3490, 3062, 2981, 2945, 2875, 1659, 1592, 1494, 1378, 1279, 1123, 1092, 1038, 775, 708.

The spectroscopic data closely match the ones previously reported in the literature. [84]

N-allyl-*N*-methylpropionamide (**5a$_{30}$**)

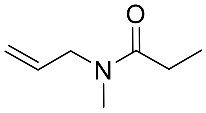

The title compound was synthesized according to the general procedure **E**. **2a$_{30}$** (71.1 mg, 1.0 mmol) and **1c** (263.1 mg, 1.1 mmol) were used to afford **5a$_{30}$** as a colourless solid (119.4 mg, 0.94 mmol, 94%).

^{1}H NMR (600 MHz, CDCl$_3$, mixture of rotamers 50/50) δ = 5.73 – 5.64 (m, 1H), 5.14 – 5.04 (m, 2H), 3.93 – 3.82 (m, 2H), 2.87 – 2.85 (d, 3H), 2.30 – 2.23 (m, 2H), 1.09 – 1.05 (dt, 3H).

^{13}C NMR (151 MHz, CDCl$_3$, mixture of rotamers) δ = 174.0, 173.5, 133.2, 132.6, 117.0, 116.4, 52.05, 49.9, 34.5, 33.6, 26.7, 26.0, 9.5, 9.2.

IR (FTIR): 3307, 3070, 2928, 1726, 1646, 1588, 1458, 1360, 1333, 1230, 1150, 1114, 967, 676.

HRMS: calculated for $C_7H_{13}NO+Na^+$: 150.0895 $[M+Na]^+$; found:150.0890.

The spectroscopic data closely match the ones previously reported in the literature. [85]

4. Green chemistry metrics calculations

Calculation of the Green Chemistry Metrics for the Mechanochemical Preparation of **3a19**

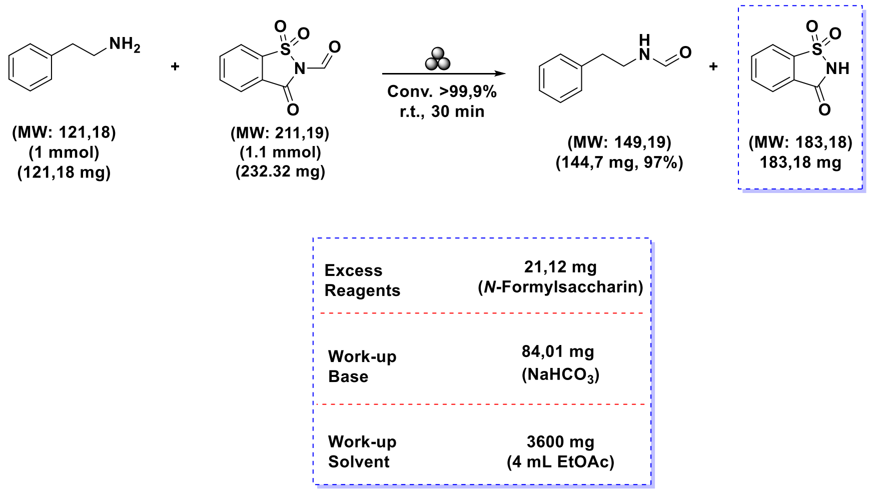

Scheme S1. Mechanochemical preparation of Formamide **3a19**.

Calculation of Green Chemistry Metrics

$$\text{Atom Economy} = \frac{\text{Mass of desired useful product}}{\text{Total Mass of all reactants}} \times 100 = \frac{149{,}19}{121{,}18 + 211{,}19} \times 100 = 45\%$$

$$\text{Environmental Factor} = \frac{\text{Mass of total waste}}{\text{Mass of desired product}} = \frac{183{,}18 + 21{,}12 + 3600{,}00 + 84{,}01}{144{,}7} = 26{,}9$$

$$\text{Reaction Mass Efficiency} = \frac{\text{actual mass of desired product}}{\text{mass of reactants}} \times 100 = \frac{144{,}7}{121{,}2 + 232{,}3} \times 100 = 41\%$$

The Eco-scale Score for the Mechanochemical Preparation of Formamide 3a19

Eco-Scale: 100 – sum of penalty points

Table S1. Calculation of Eco-scale score[a]

Reagents	MF	MW	G	mmol	Equiv.
Phenetylamine	$C_8H_{11}N$	121.18	0.1212	1.00	1.00
N-Formylsaccharin	$C_8H_5NO_4S$	211.19	0.2323	1.10	1.10
**Product**	**MF**	**MW**	**G**	**mmol**	**Yield**
Formamide, *N*-(2-phenylethyl)-	$C_9H_{11}NO$	149.19	0.1447	0.97	97%

Entry	Parameters	Penalty Points
1	Yield (97%)	-1.5
2	Price/availability	-5
3	Safety	0
4	Technical set-up (Common set-up)	0
5	Temperature/time (r.t.; < 1 h)	0
6	Work-up and purification (adding a solvent)	0
	**Eco-Scale Score**	93.5

[a]Values calculated using the eco scale calculator software available at the link: http://ecoscale.cheminfo.org/calculator

Calculation of the Green Chemistry Metrics for the in-solution preparation of **3a**[19]**.**

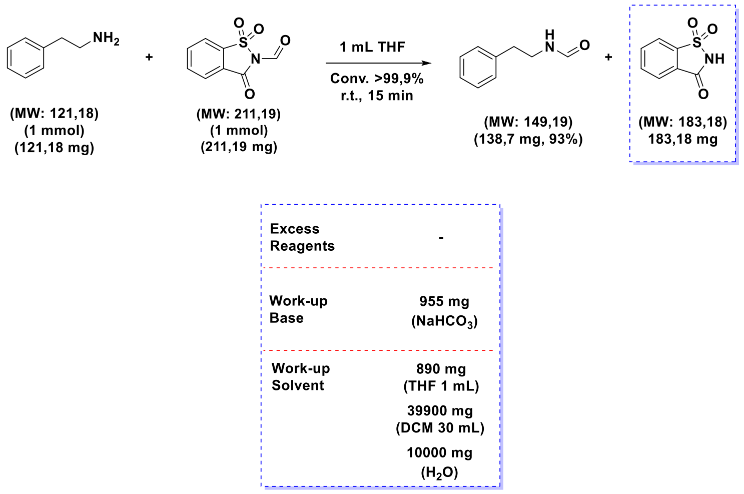

Scheme S2. In-solution* preparation of formamide **3a**[19].

* T. Cochet;V. Bellosta; A. Greiner; D. Roche; J. Cossy *Synlett* **2011**, *13*, 1920–1922.

Calculation of Green Chemistry Metrics

$$\text{Atom Economy} = \frac{\text{Mass of desired useful product}}{\text{Total Mass of all reactants}} \times 100 = \frac{149{,}19}{121{,}18 + 211{,}19} \times 100 = 45\%$$

$$\text{Environmental Factor} = \frac{\text{Mass of total waste}}{\text{Mass of desired product}} = \frac{183{,}2 + 955{,}0 + 890{,}0 + 39900{,}0 + 10000{,}0}{138{,}7} = 374{,}4$$

$$\text{Reaction Mass Efficiency} = \frac{\text{actual mass of desired product}}{\text{mass of reactants}} \times 100$$

$$\text{RME} = \frac{138{,}7}{121{,}18 + 211{,}19} \times 100 = 42\%$$

The Eco-scale Score for the In-solution Preparation of Formamide 3a[19]

Eco-Scale: 100 – sum of penalty points

Table S2. Calculation of Eco-scale score[a]

Reagents	MF	MW	G	mmol	Equiv.
Phenetylamine	$C_8H_{11}N$	121.18	0.1212	1.00	1.00
N-Formylsaccharin	$C_8H_5NO_4S$	211.19	0.2112	1.00	1.00
**Product**	**MF**	**MW**	**G**	**mmol**	**Yield**
Formamide, *N*-(2-phenylethyl)-	$C_9H_{11}NO$	149.19	0.1387	0.93	93%

Entry	Parameters	Penalty Points
1	Yield (93%)	-3.5
2	Price/availability	-3
3	Safety	0
4	Technical set-up (Common set-up)	0
5	Temperature/time (r.t.; < 1 h)	0
6	Work-up and purification (adding a solvent, liquid-liquid extraction or washing)	-3
	**Eco-Scale Score**	90.5

[a]Values calculated using the eco scale calculator software available at the link: http://ecoscale.cheminfo.org/calculator

5. Spectra

2-Acetylbenzo[d]isothiazol-3(2H)-one 1,1-dioxide (**1b**)

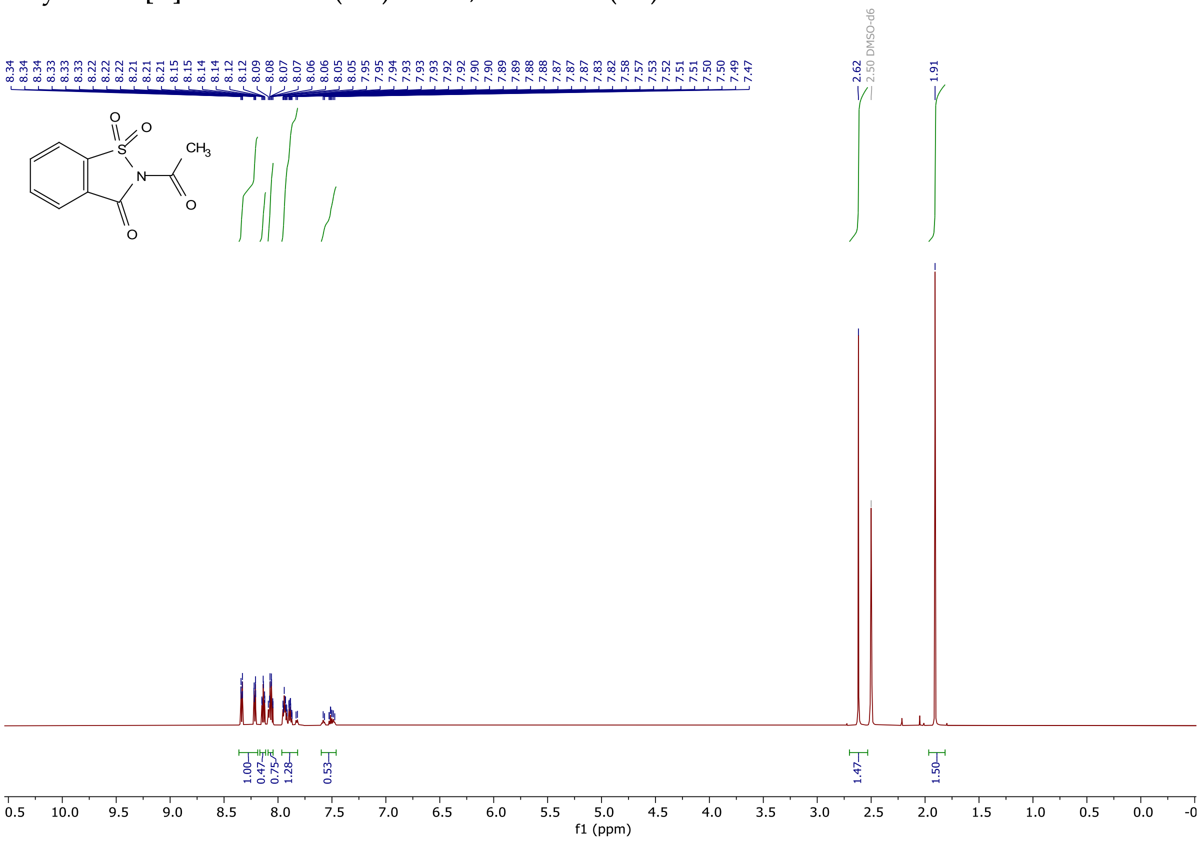

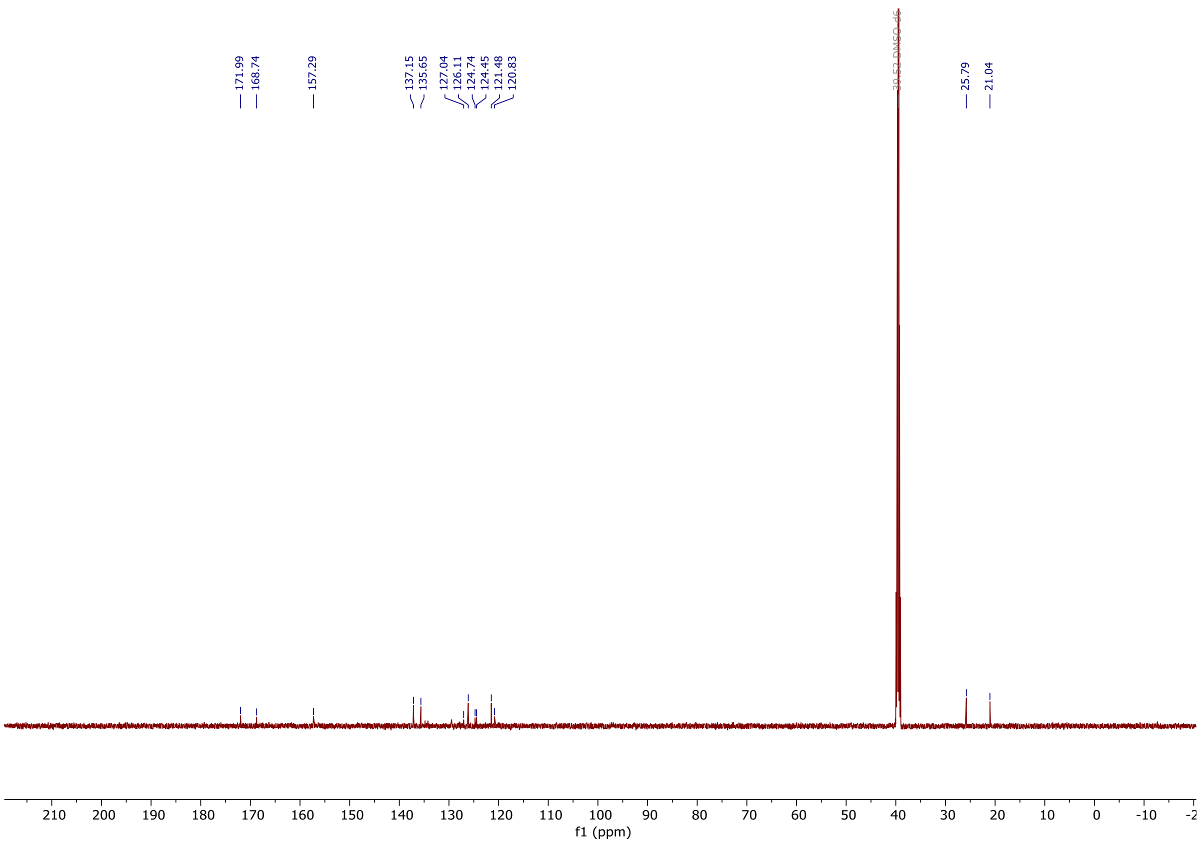

2-Propionylbenzo[d]isothiazol-3(2H)-one 1,1-dioxide (**1c**)

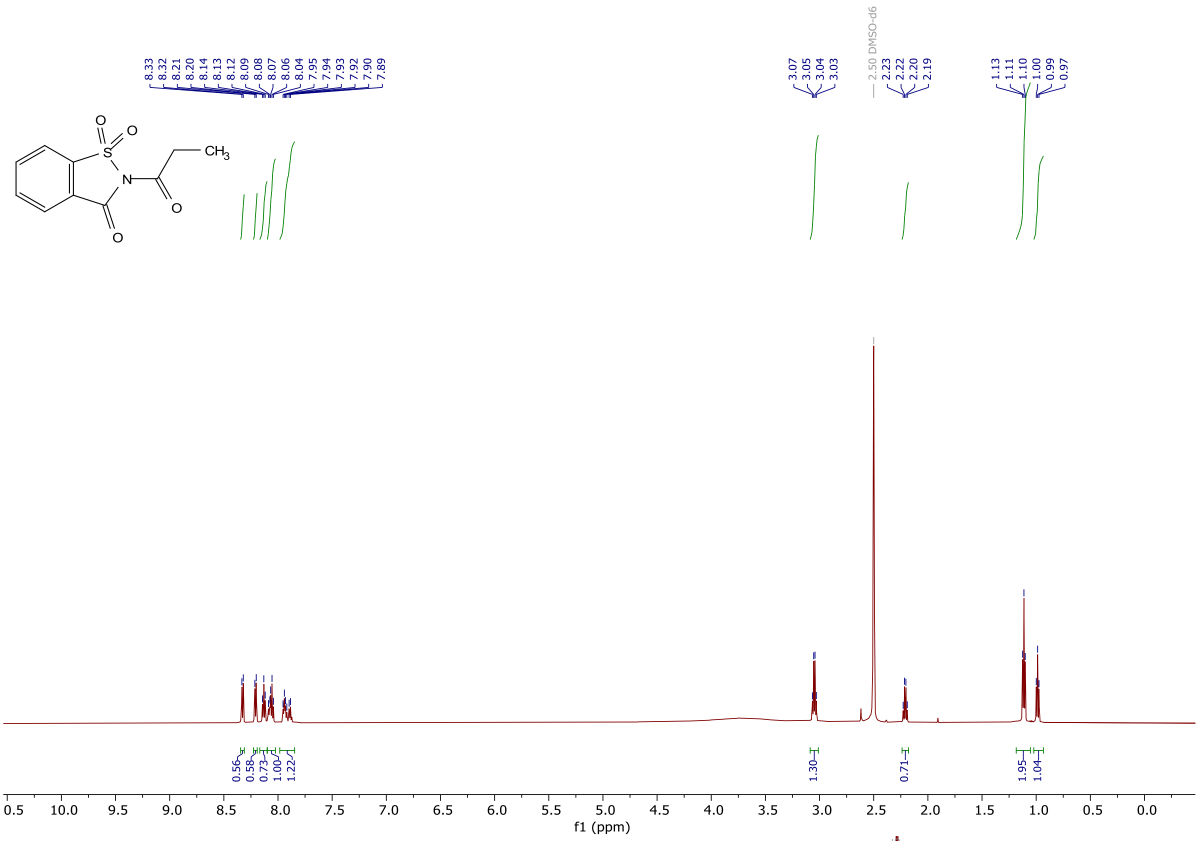

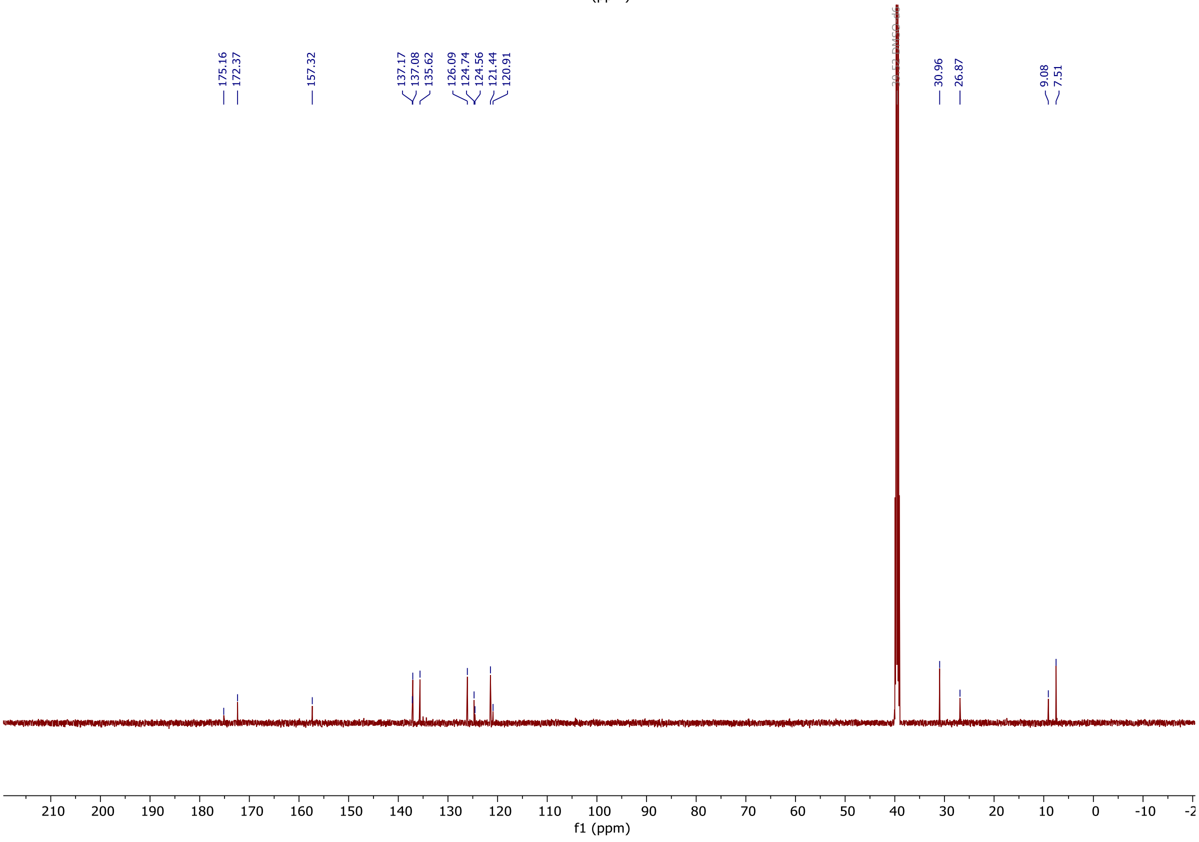

N-phenyl formamide (**3a₁**)

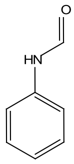

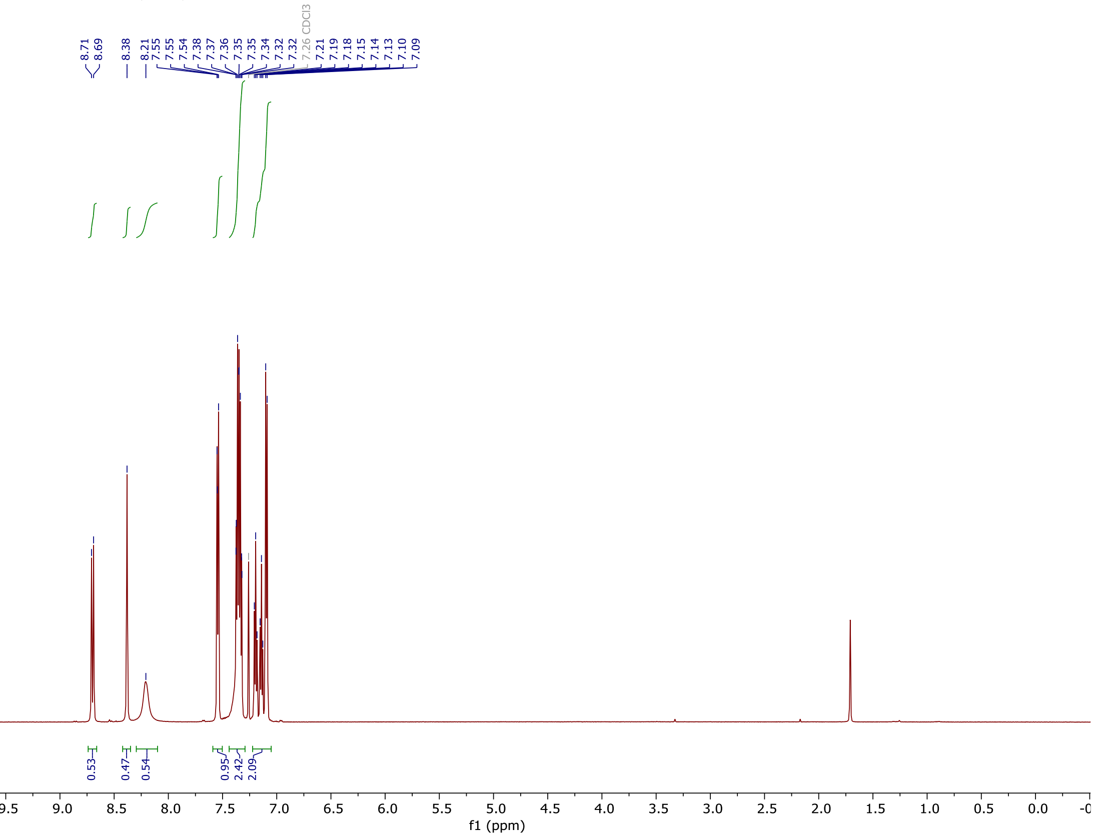

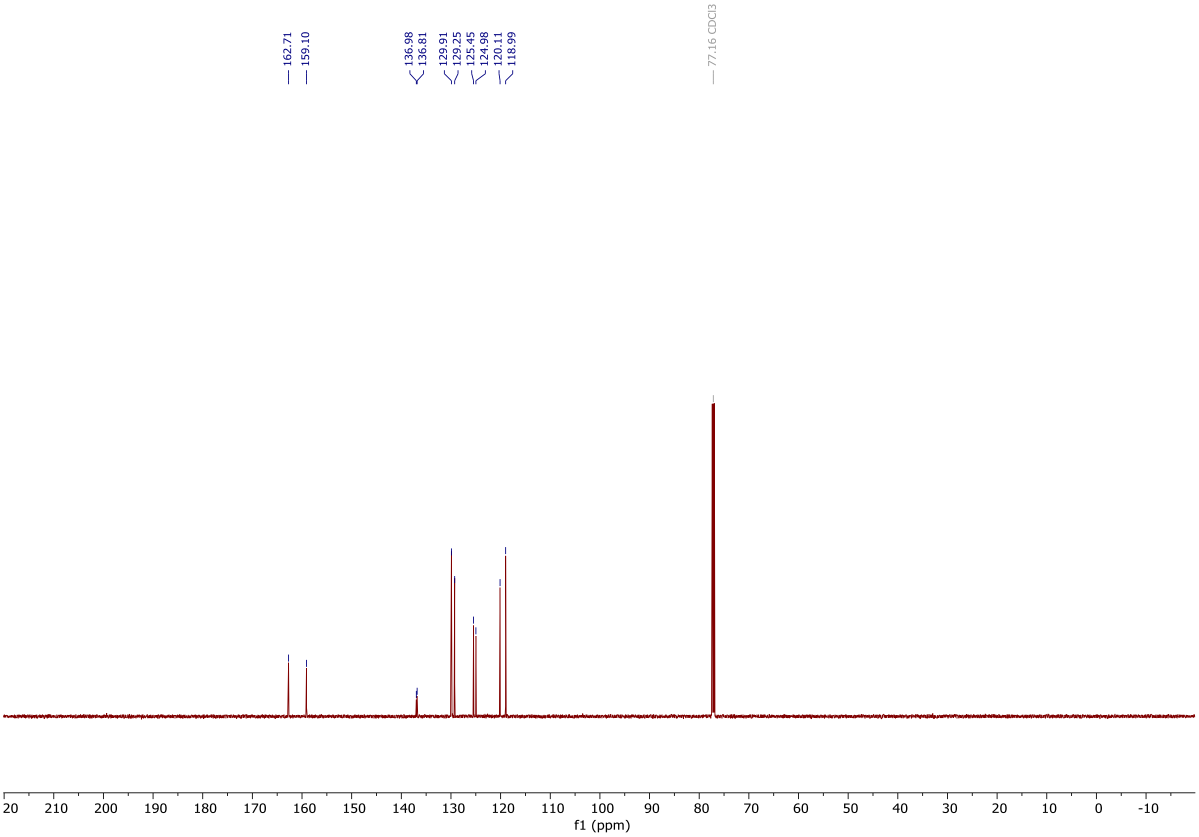

N-(2-fluorophenyl) formamide (**3a**2)

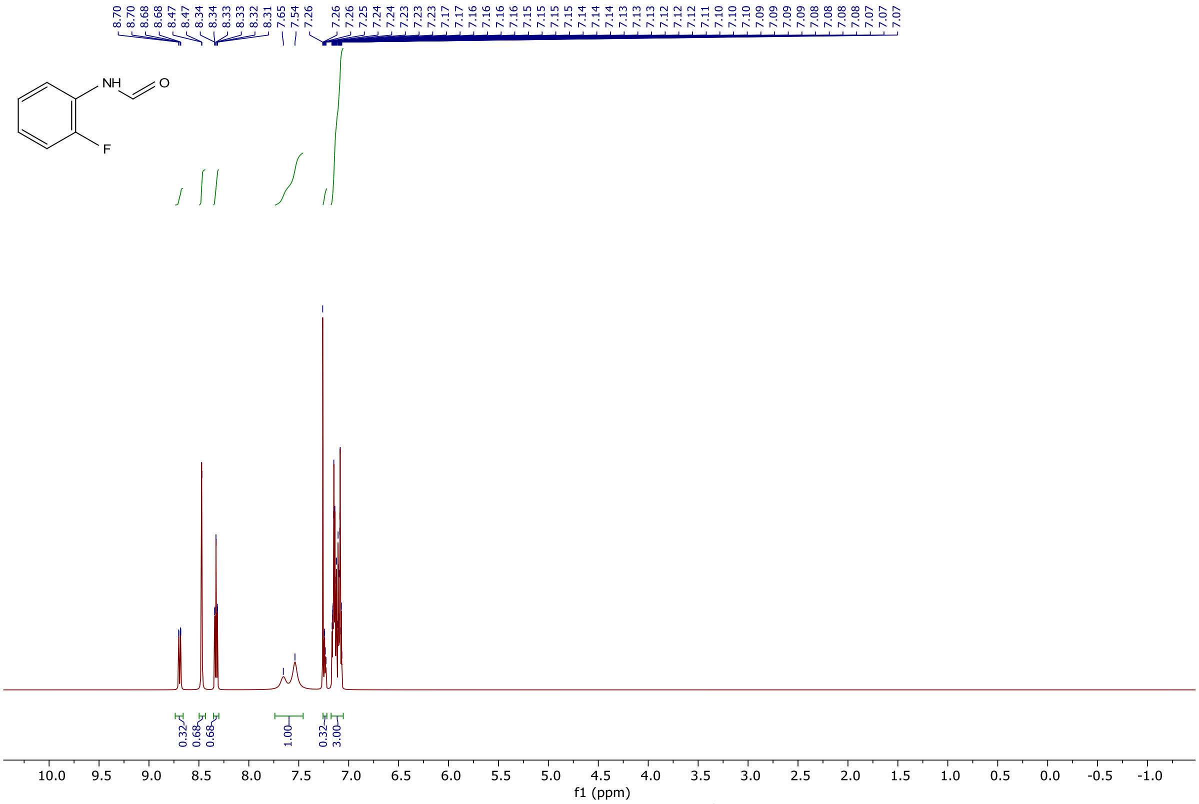

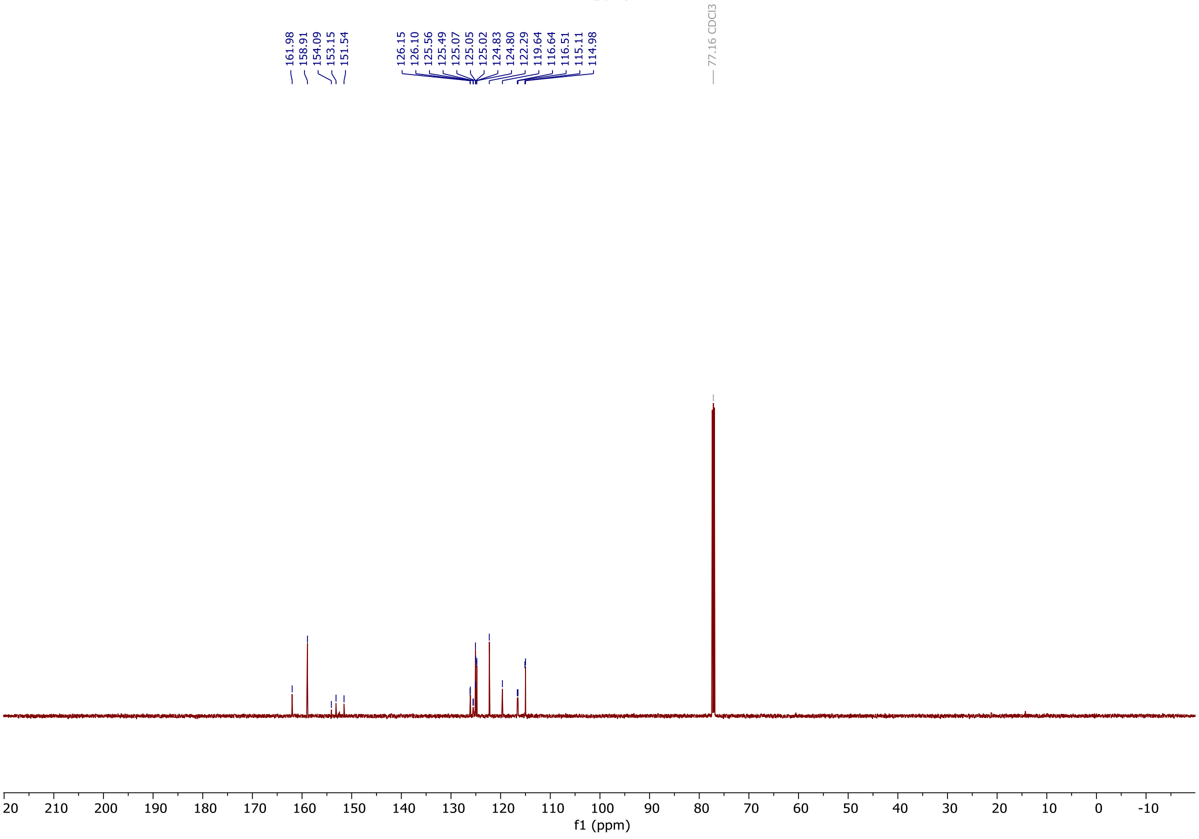

N-(4-fluorophenyl) formamide (**3a**3)

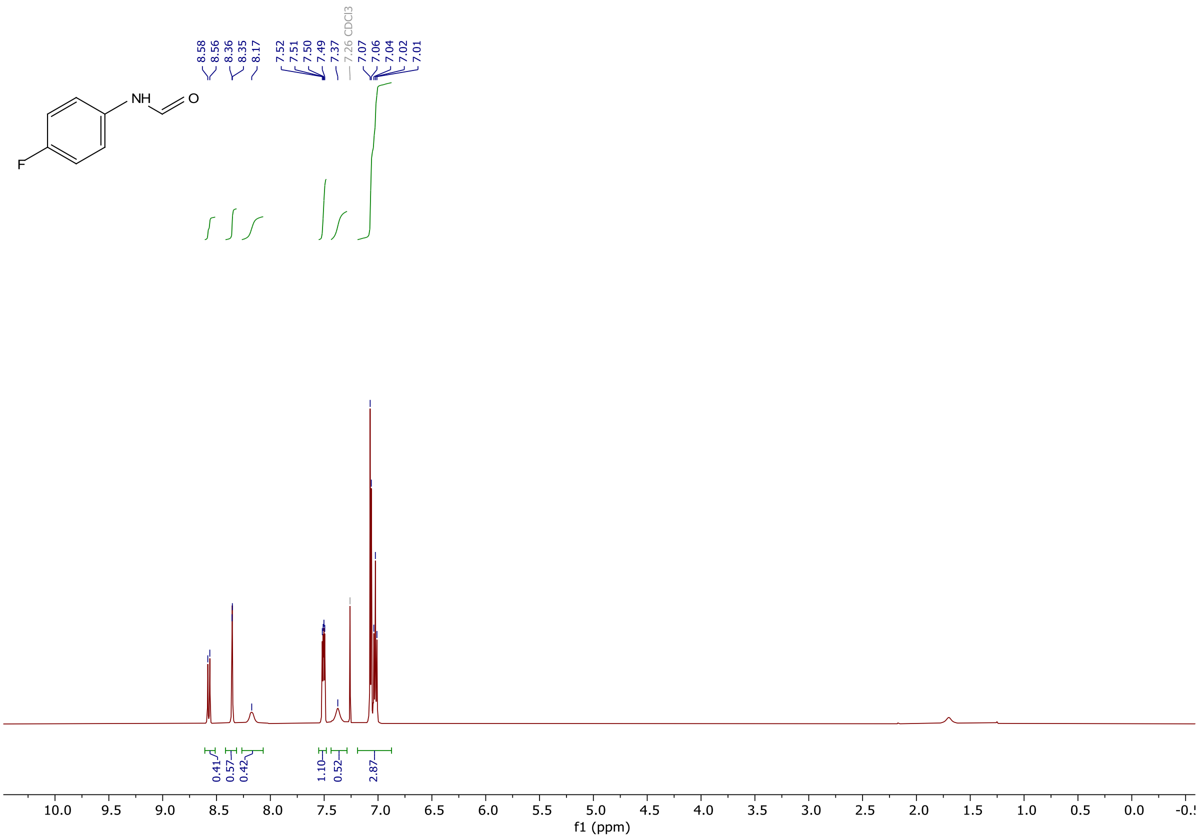

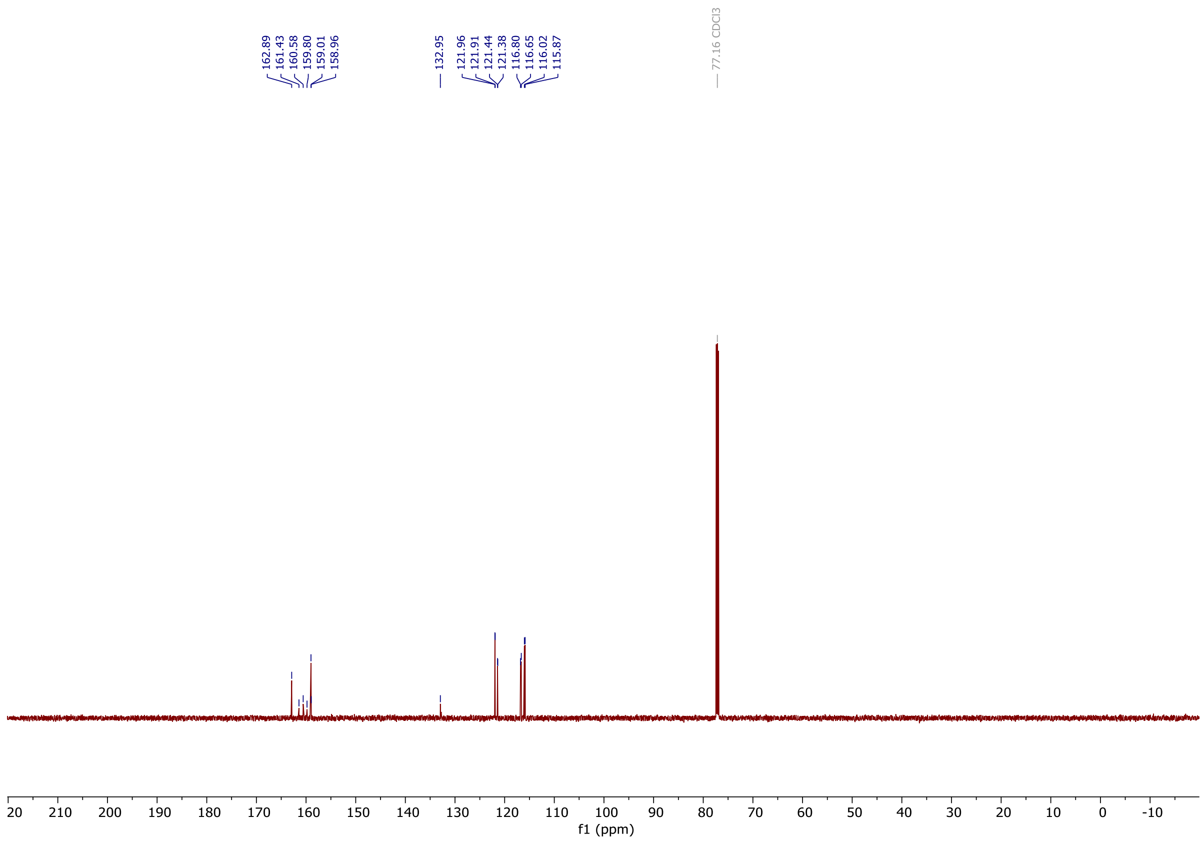

N-(4-chlorophenyl) formamide (**3a**4)

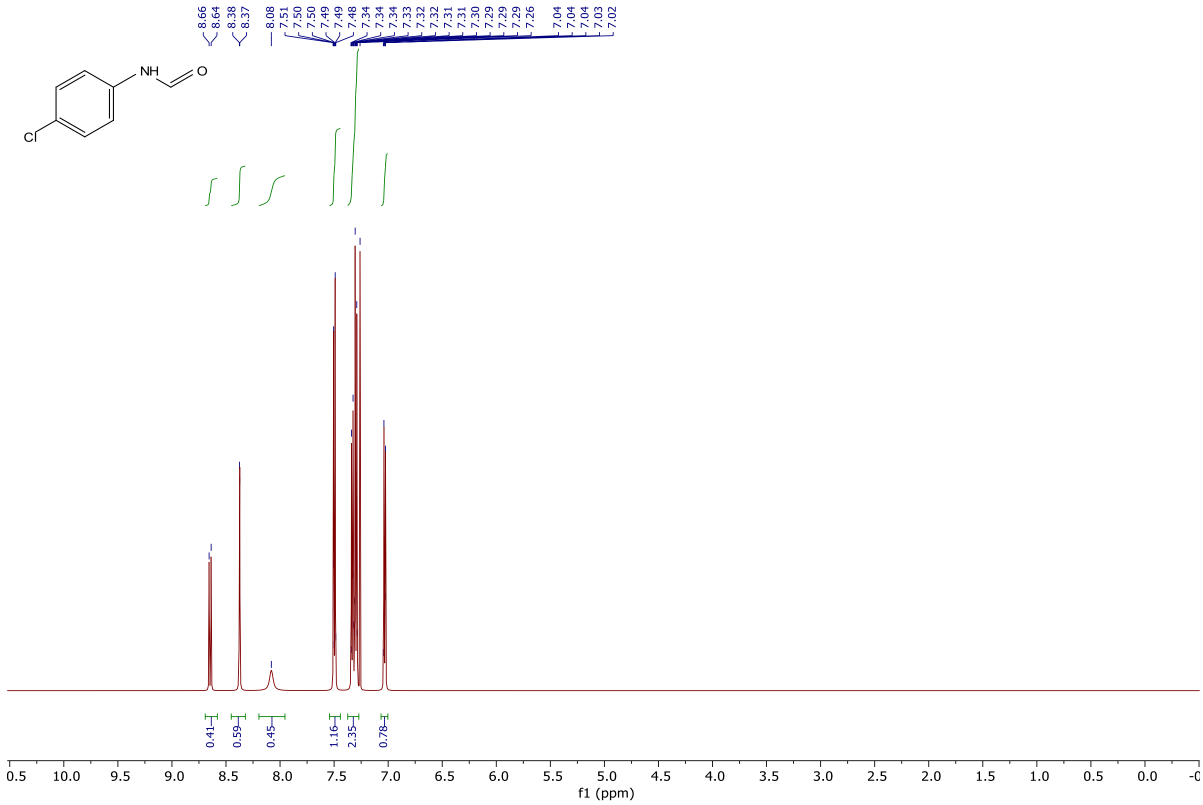

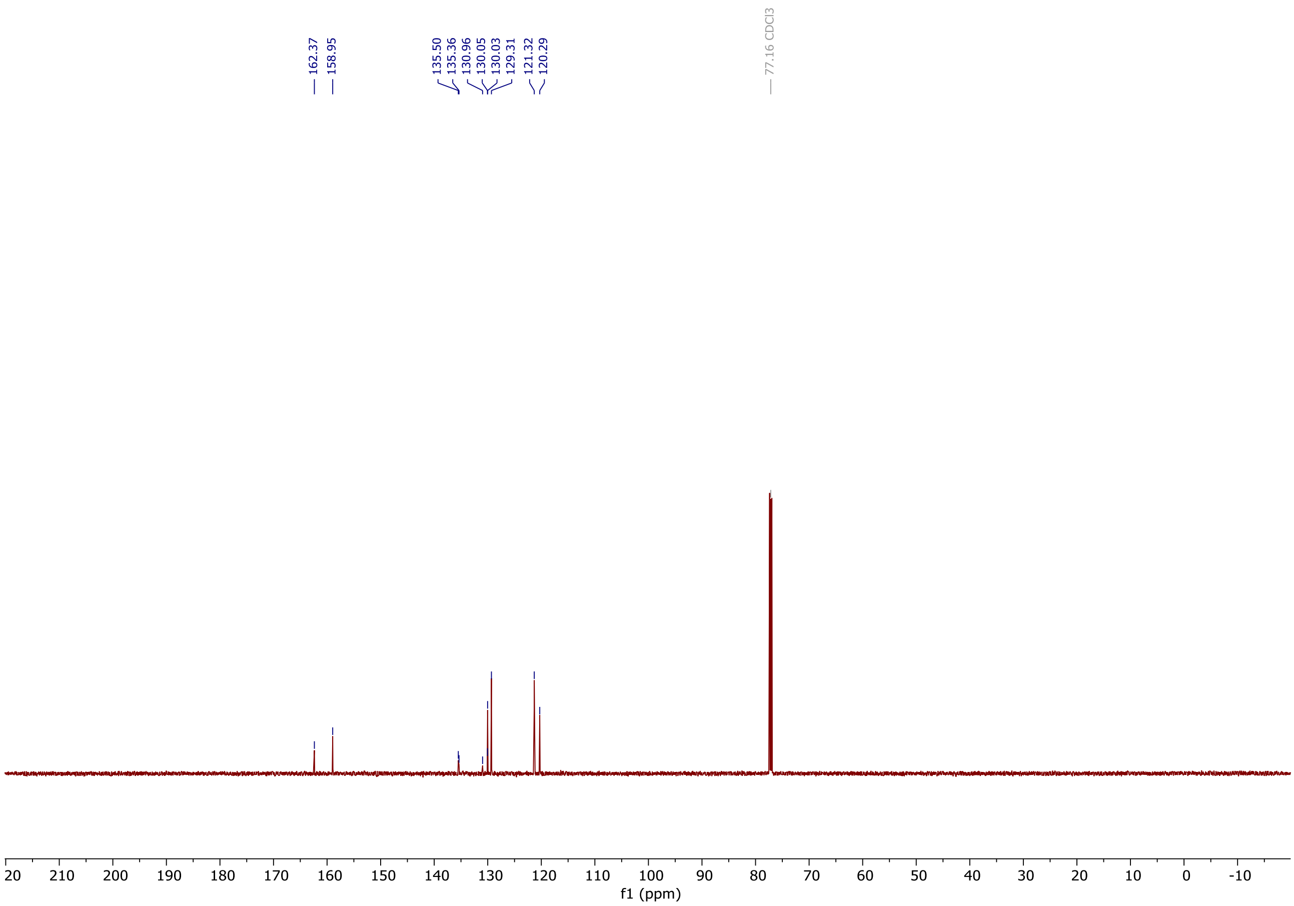

N-(4-bromophenyl) formamide (**3a5**)

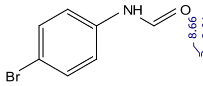

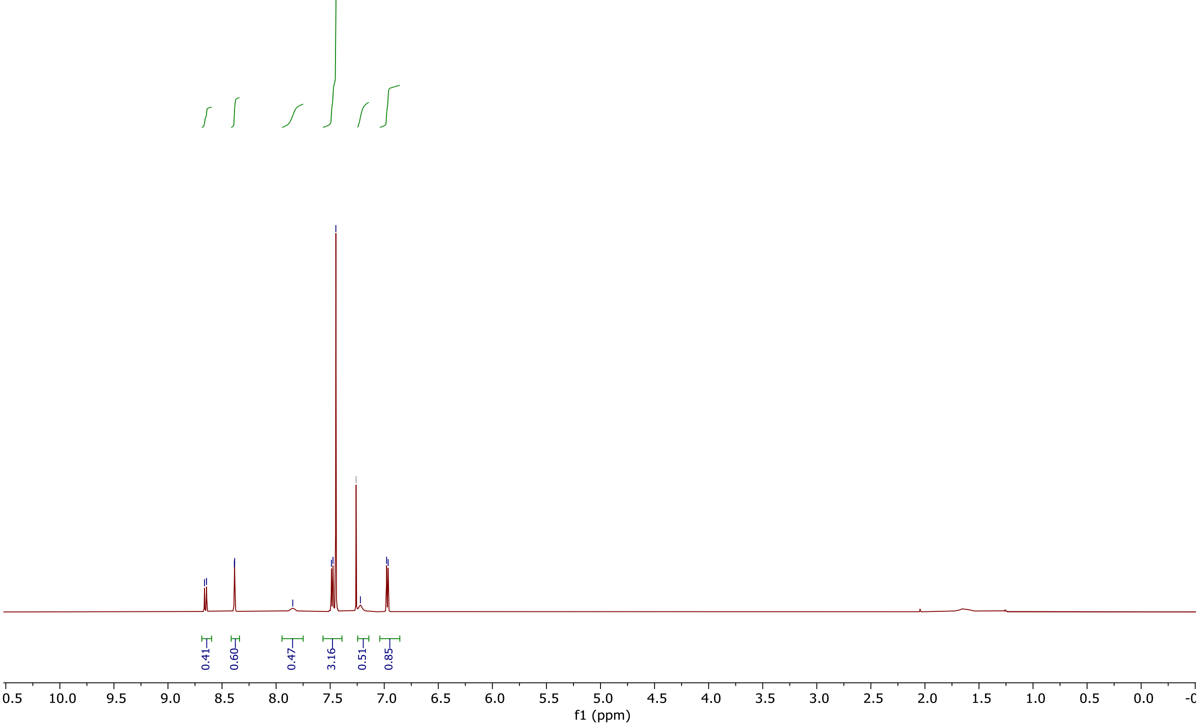

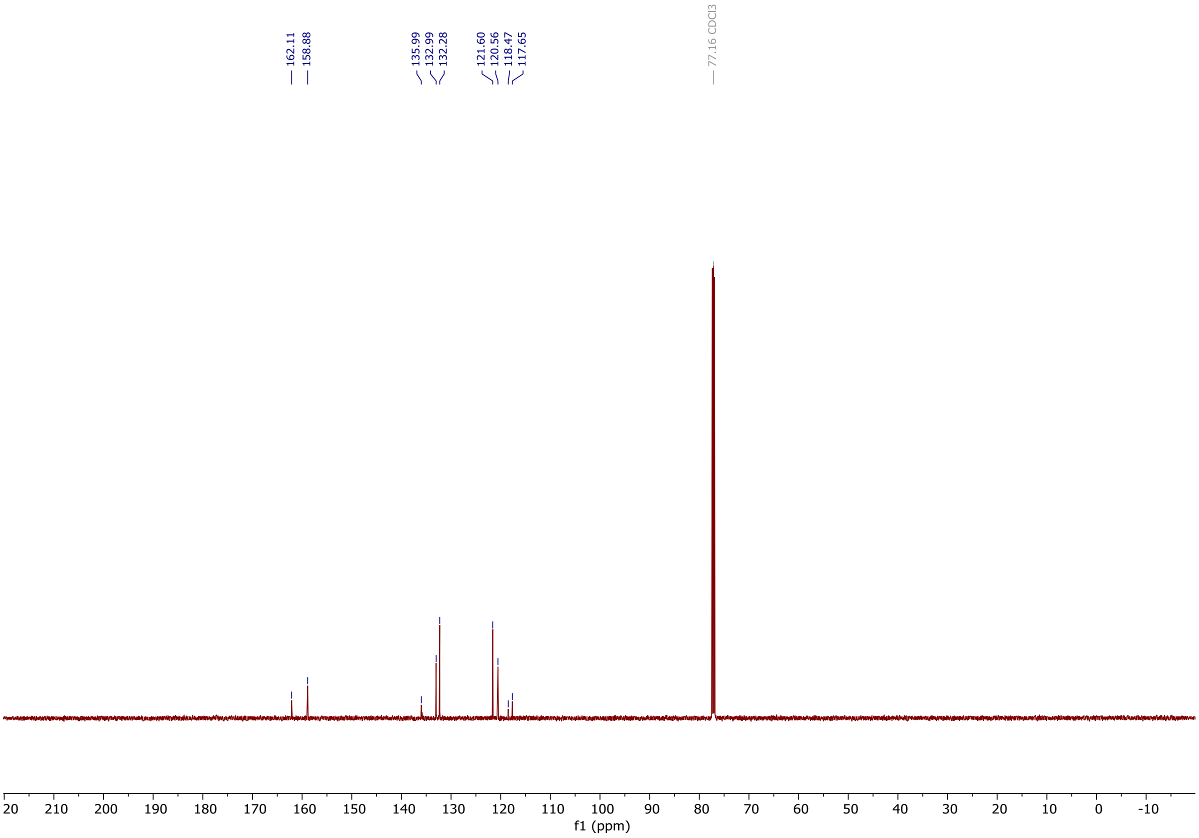

N-(2-iodophenyl) formamide (**3a**$_{6}$)

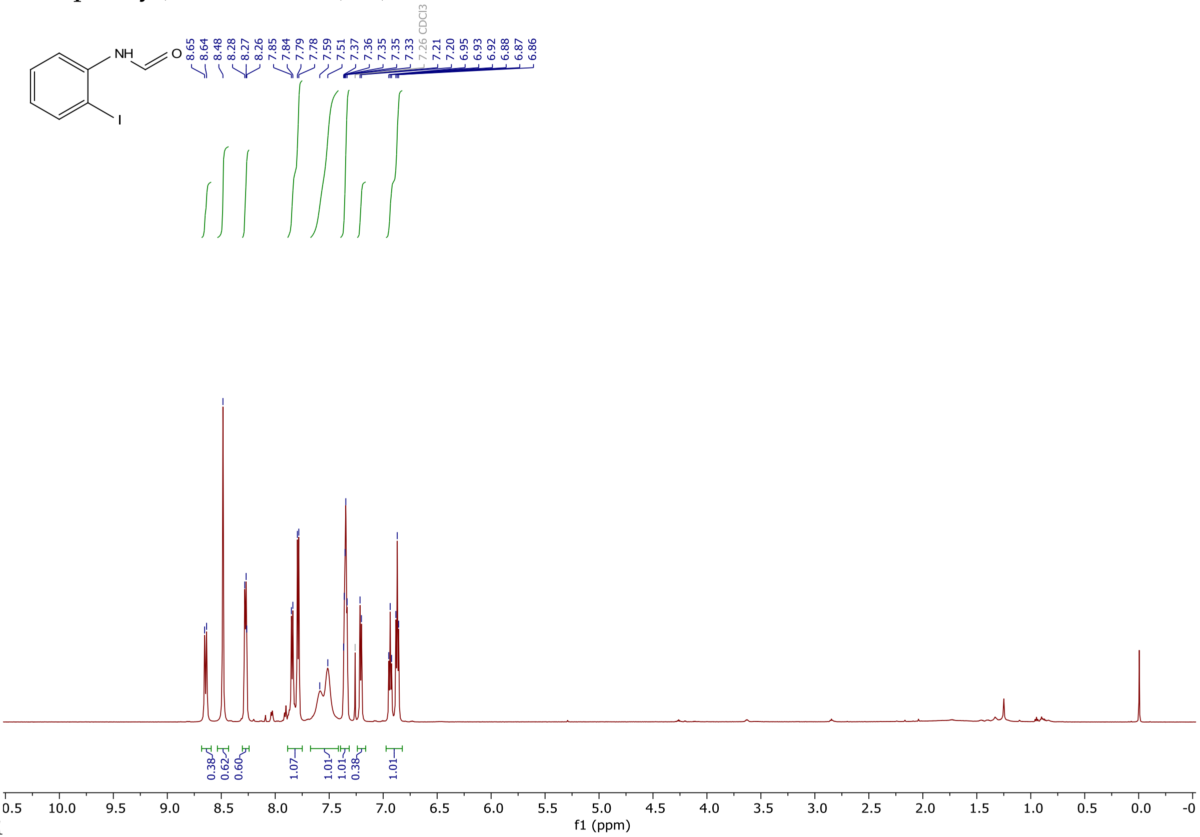

ù

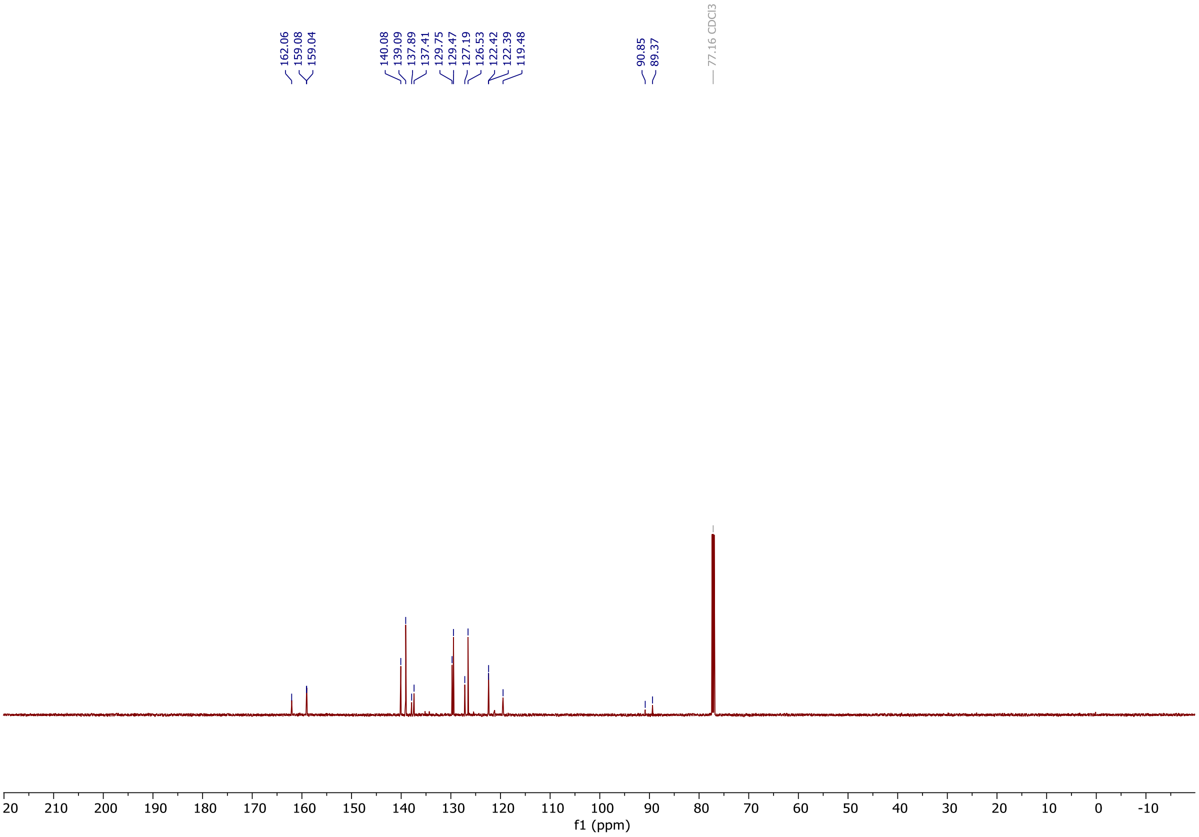

N-(2,4-dimethyl) phenyl formamide (**3a**7)

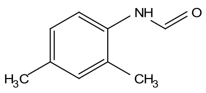

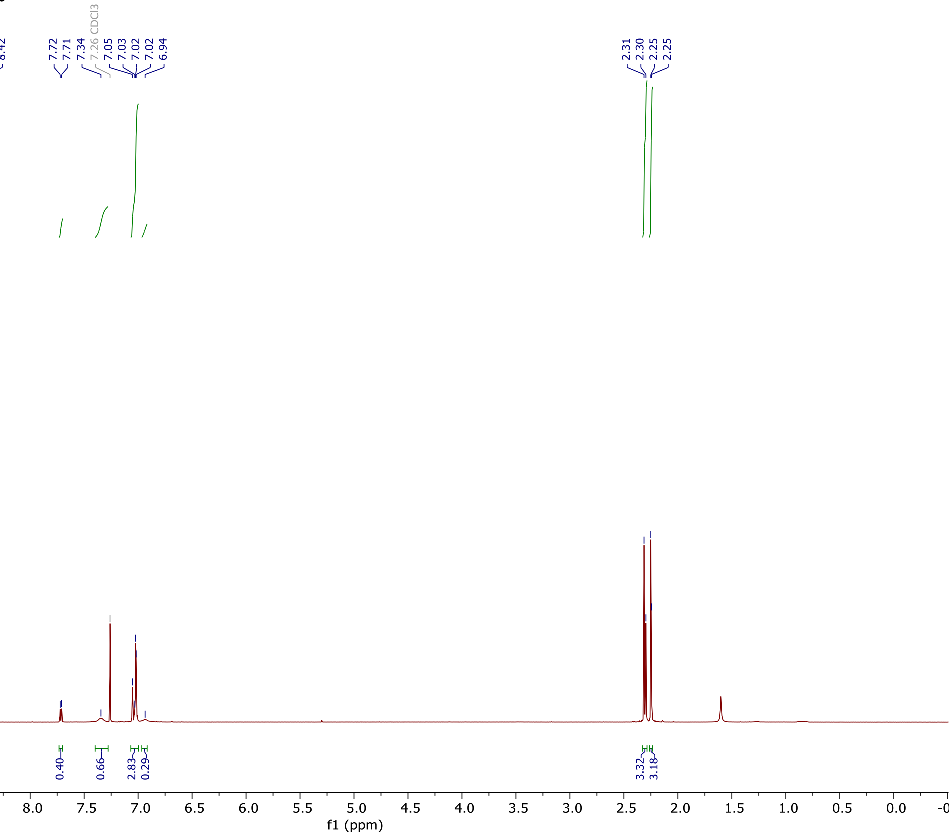

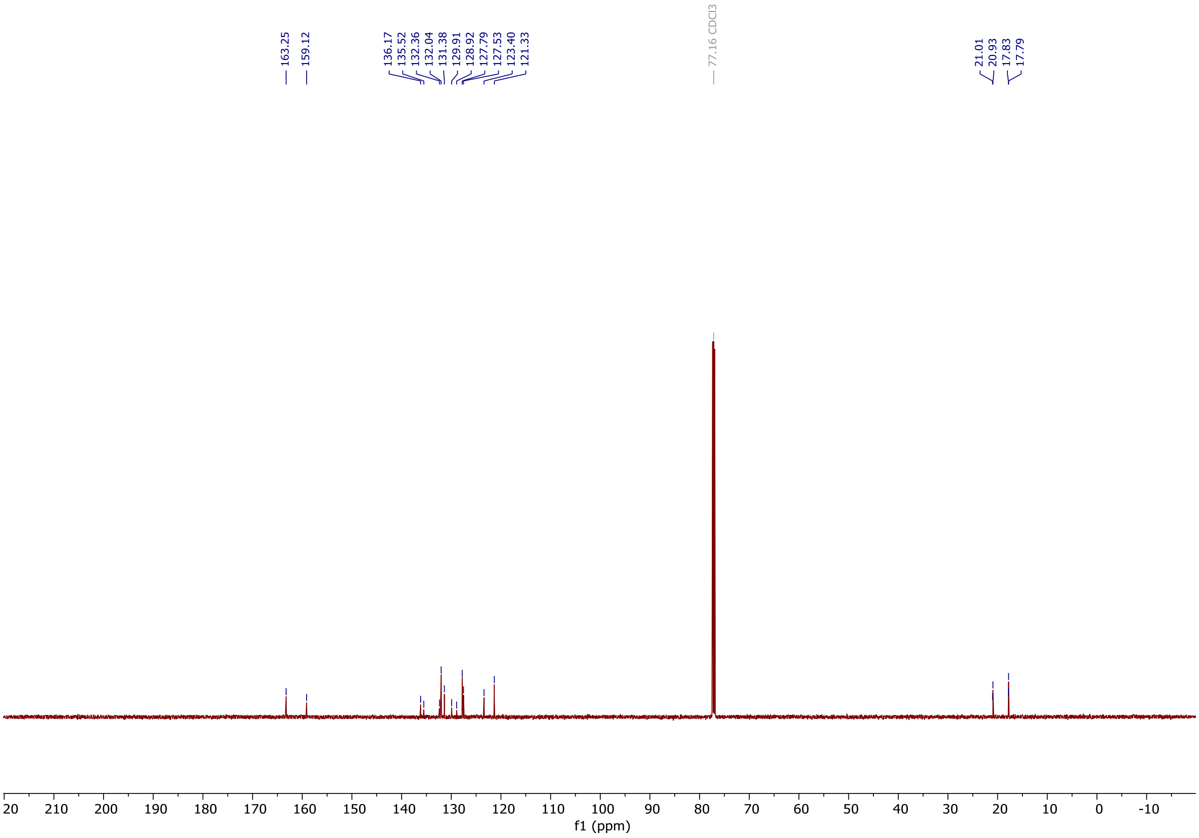

N-mesityl formamide (**3a8**)

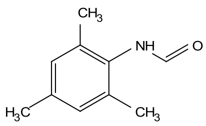

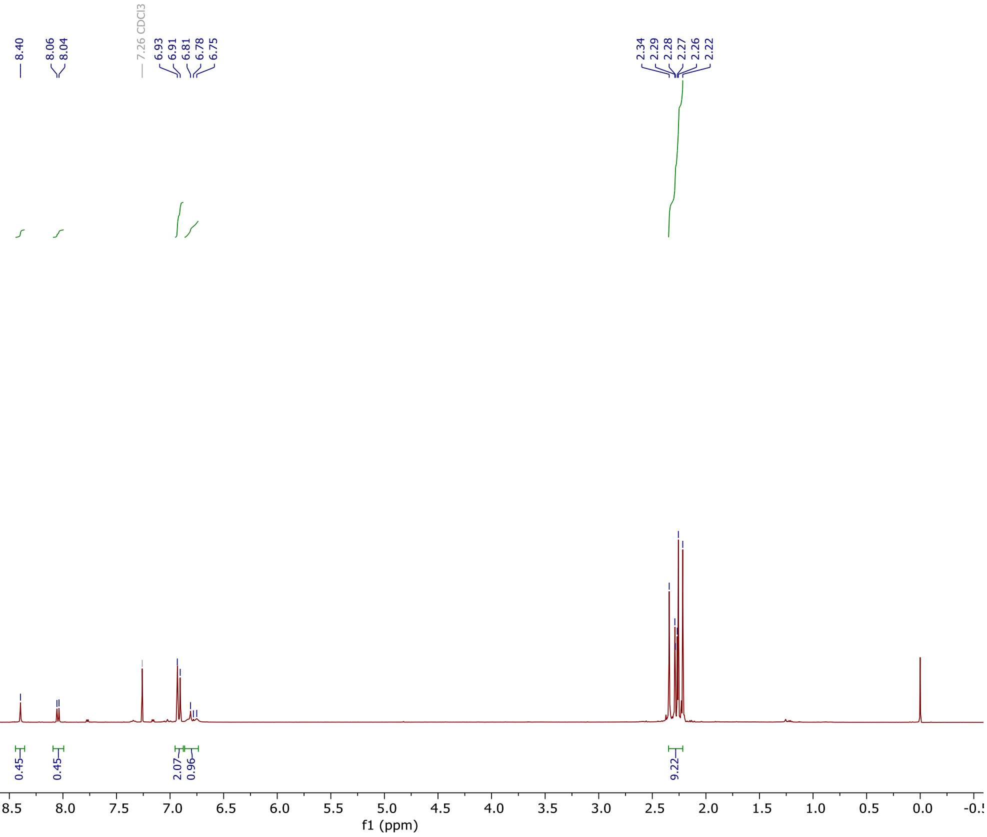

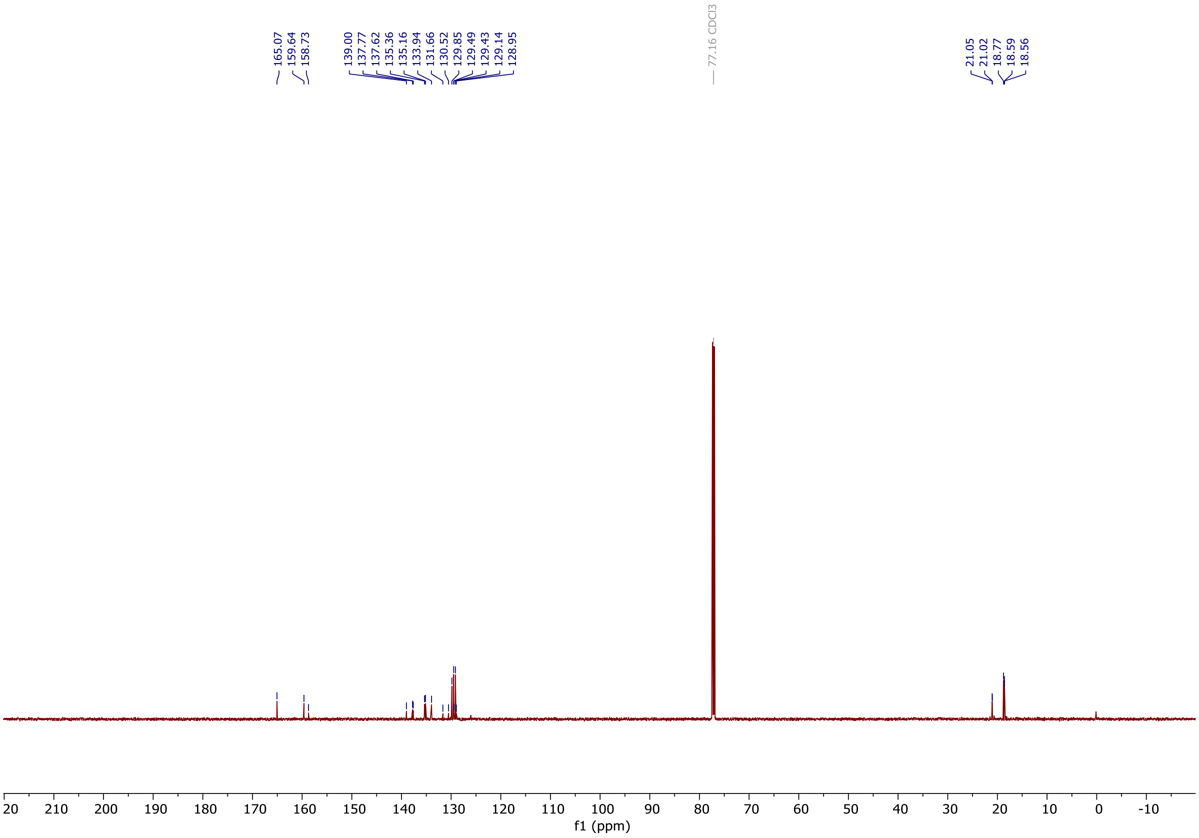

N-(4-(*tert*-butyl) phenyl) formamide (**3a₉**)

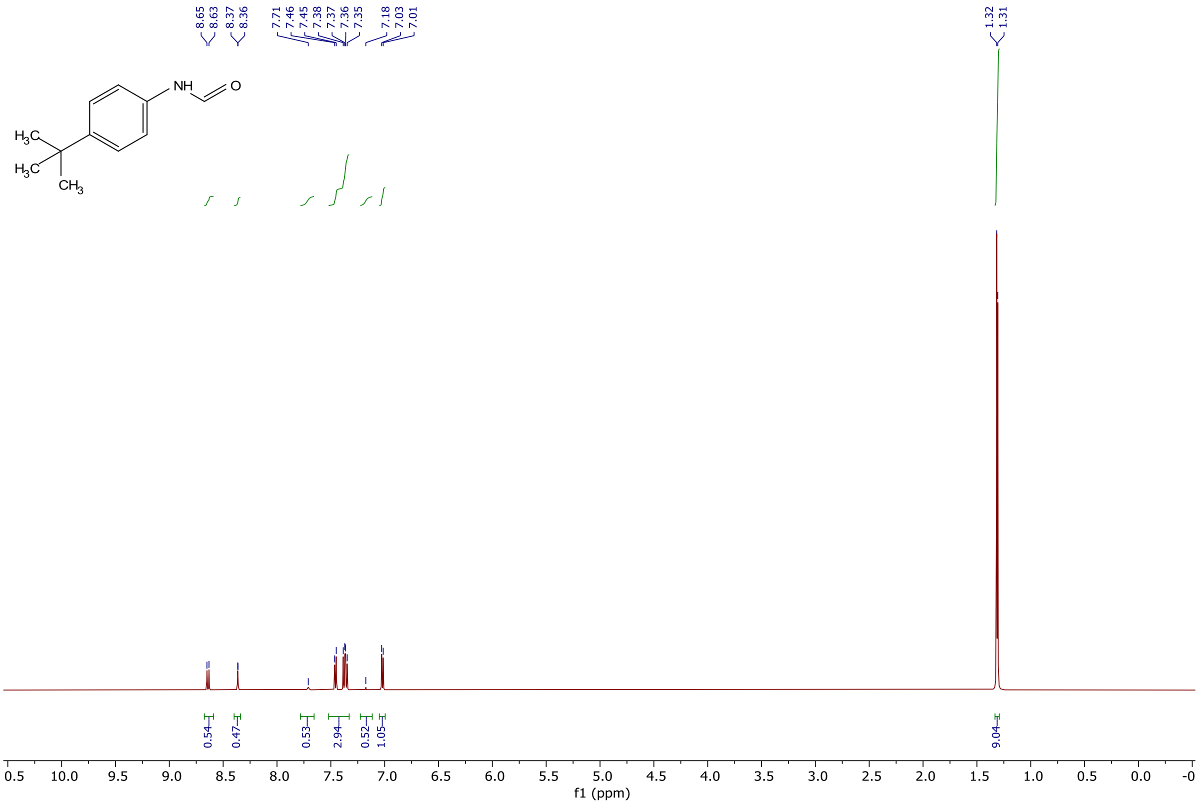

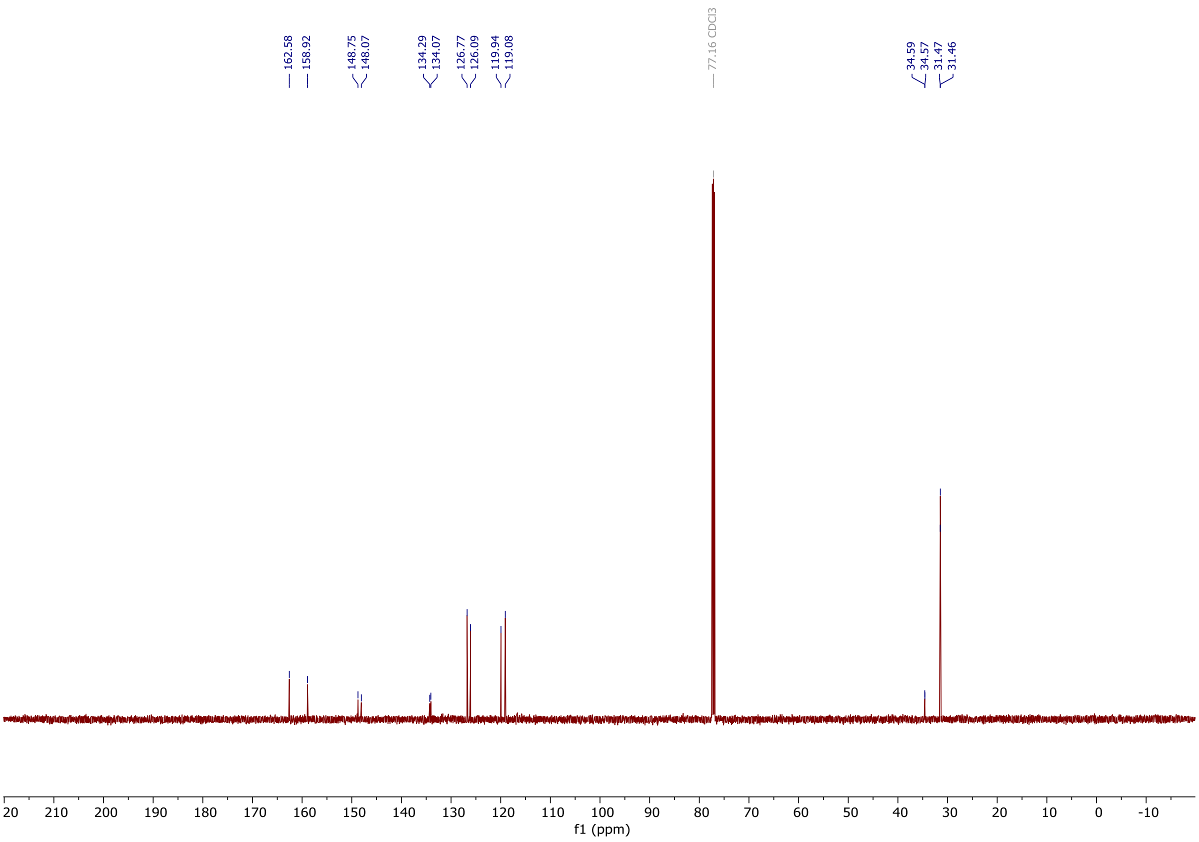

N-(2-(methylthio) phenyl) formamide (**3a10**)

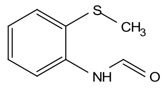

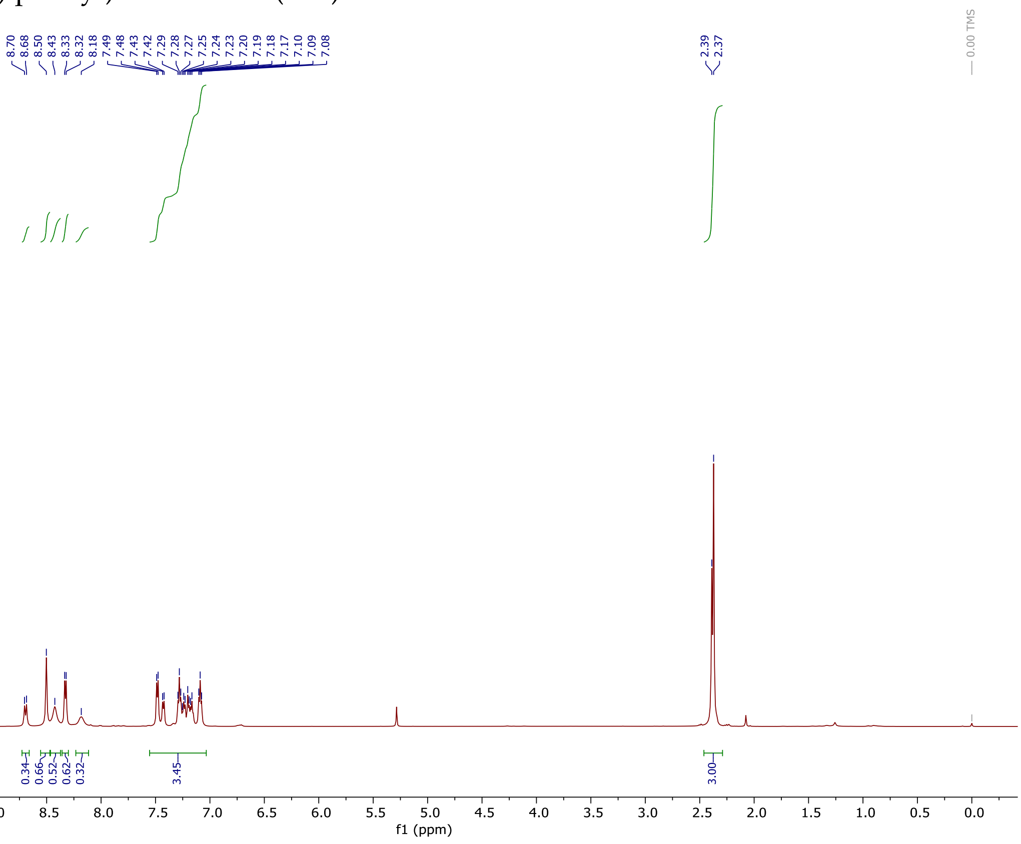

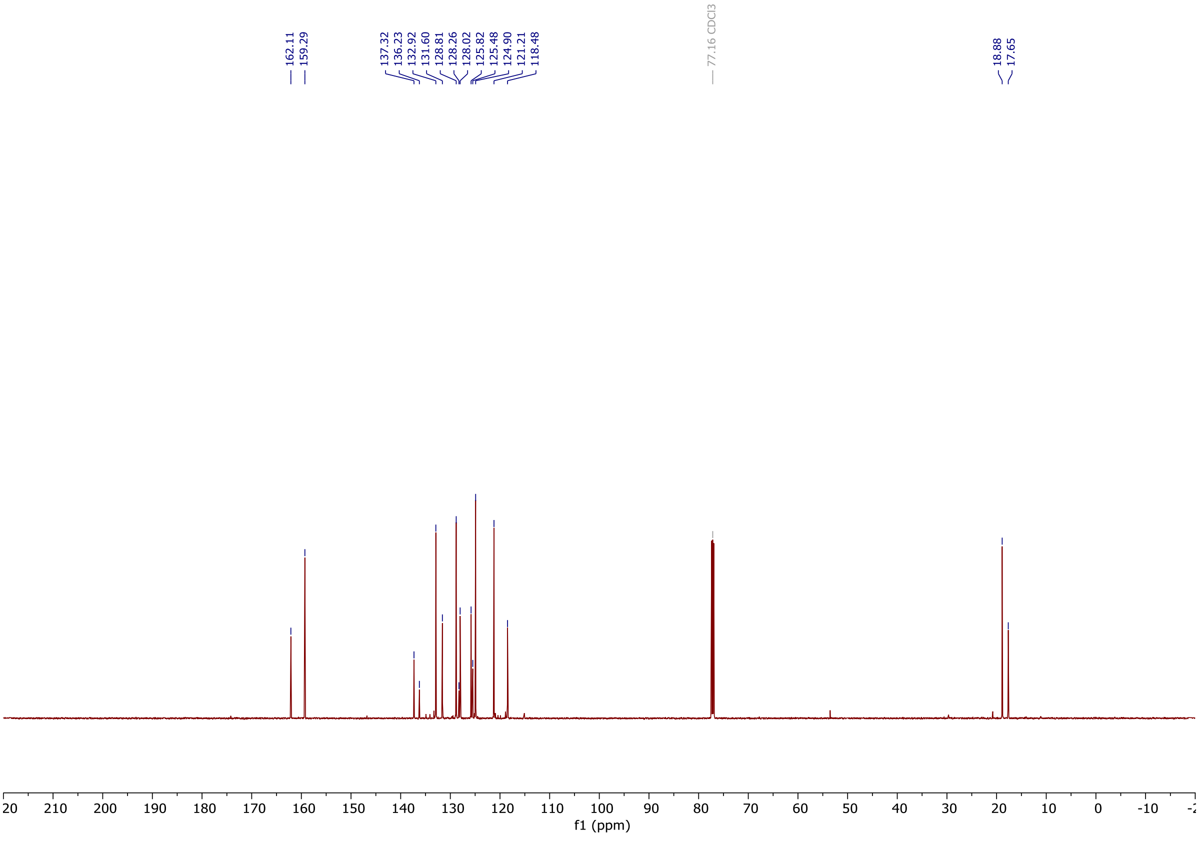

N-(4-hydroxyphenyl) formamide (**3a**11)

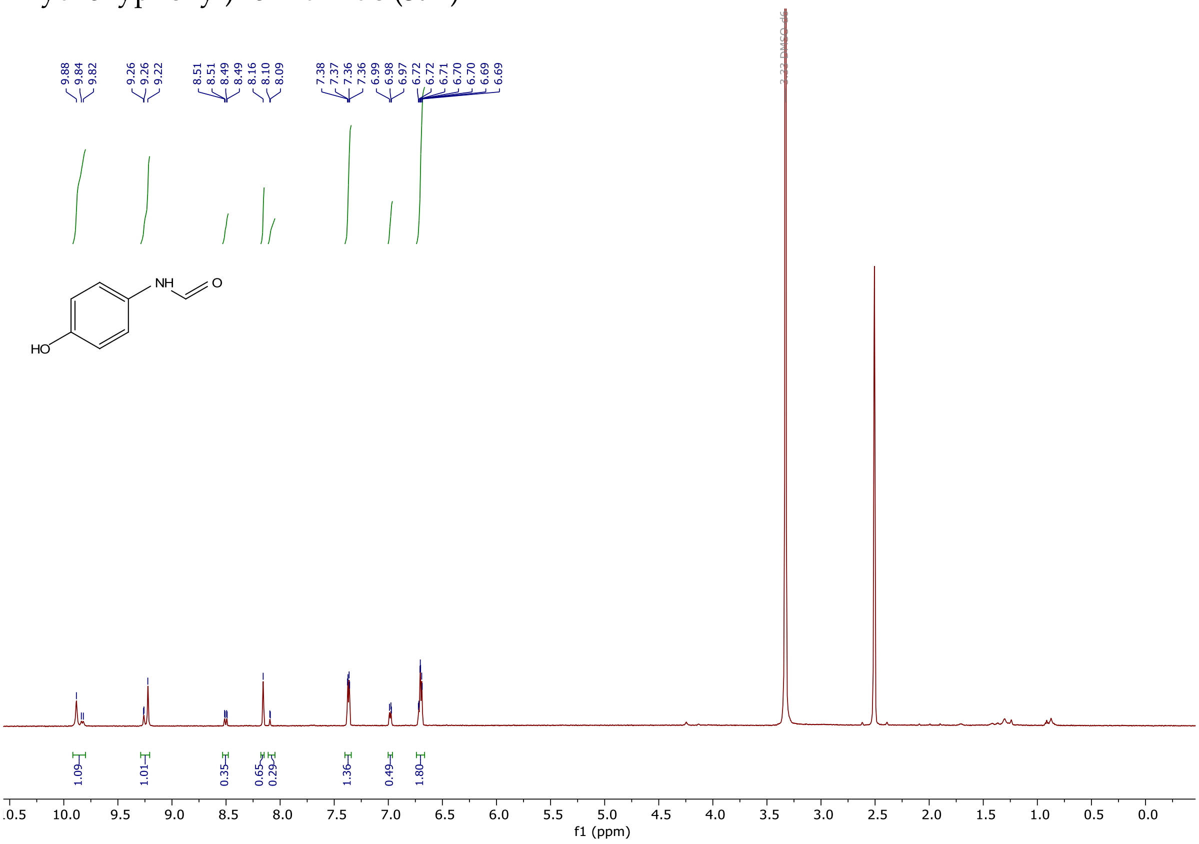

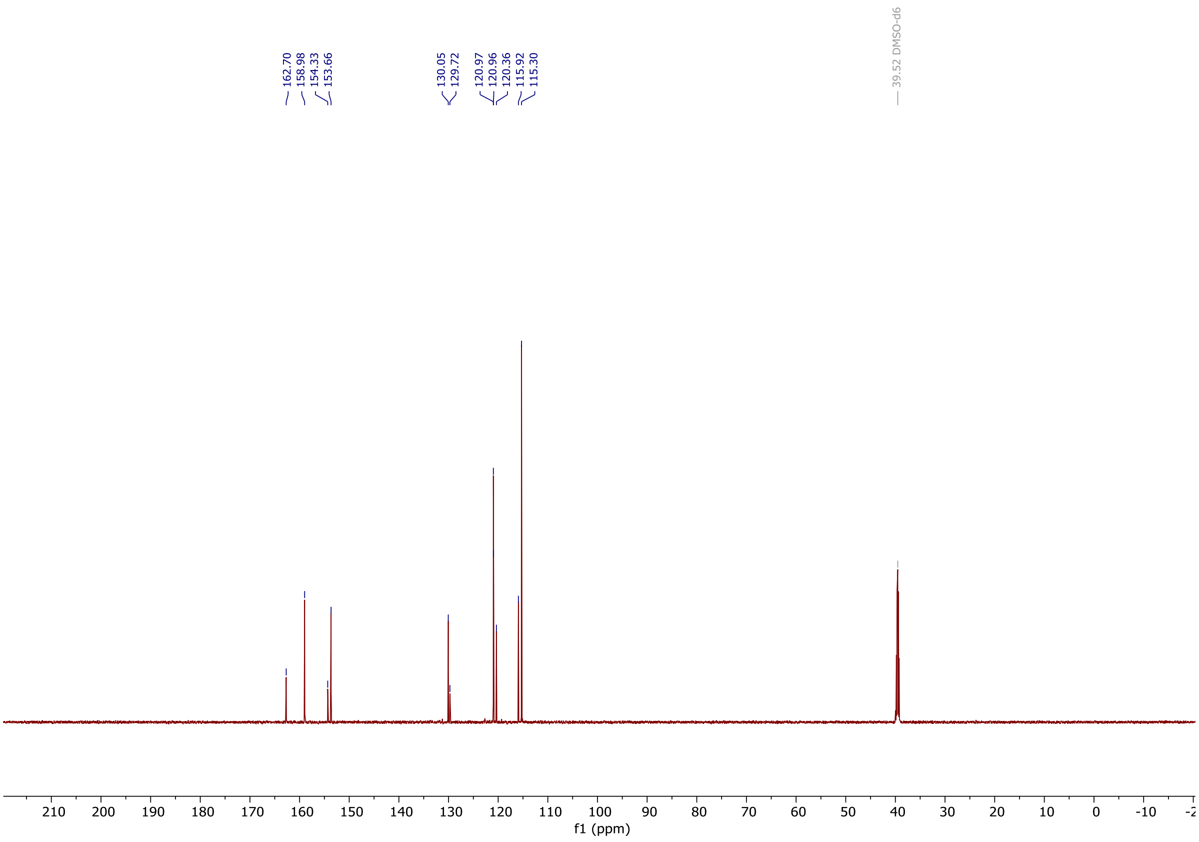

N-(4-methoxyphenyl) formamide (**3a12**)

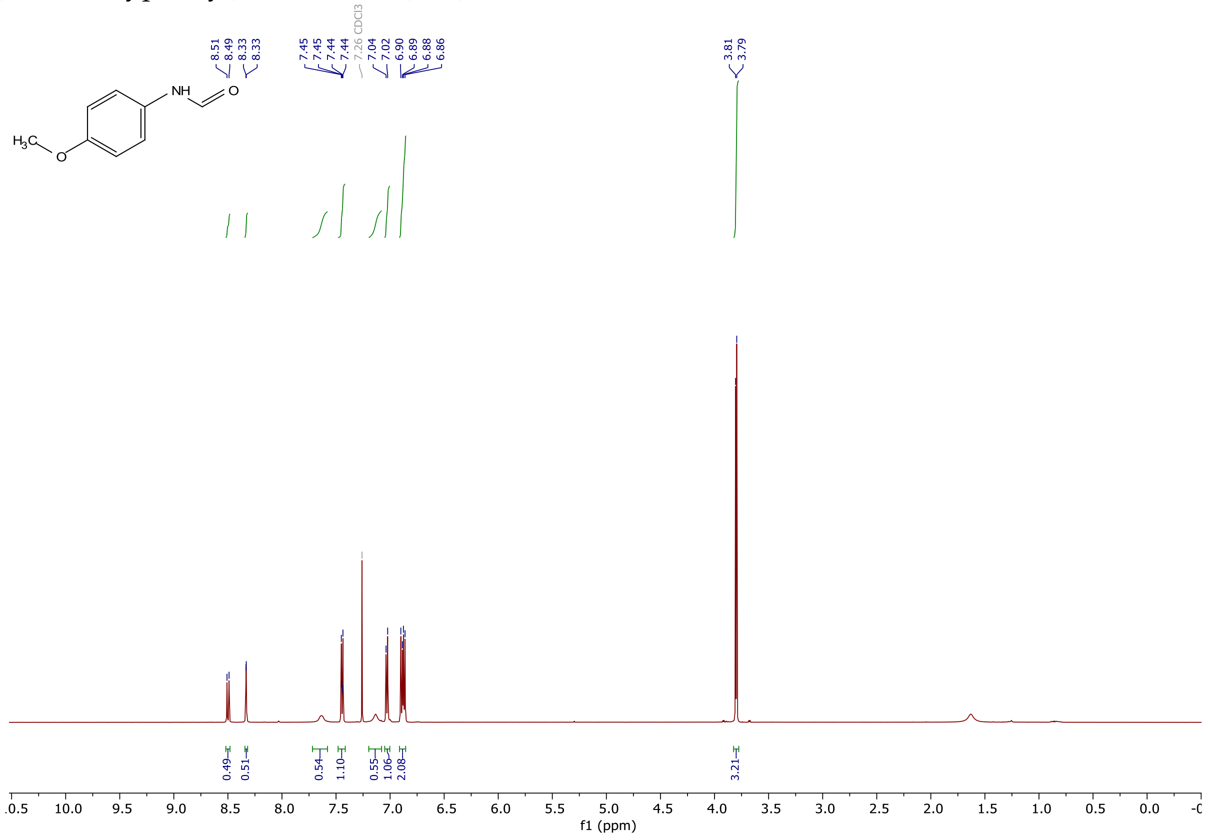

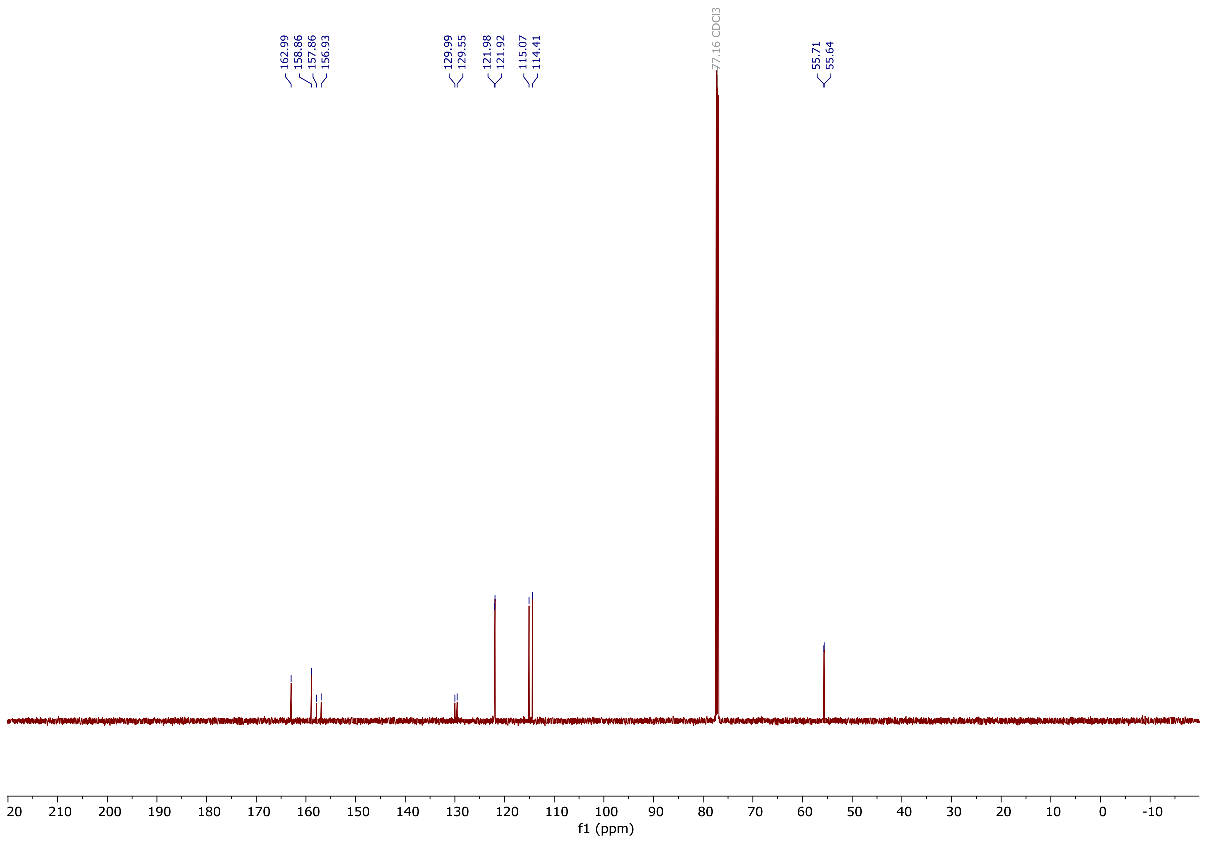

N-(2-(phenylamino) phenyl) formamide (**3a**13)

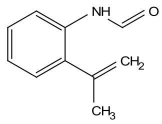

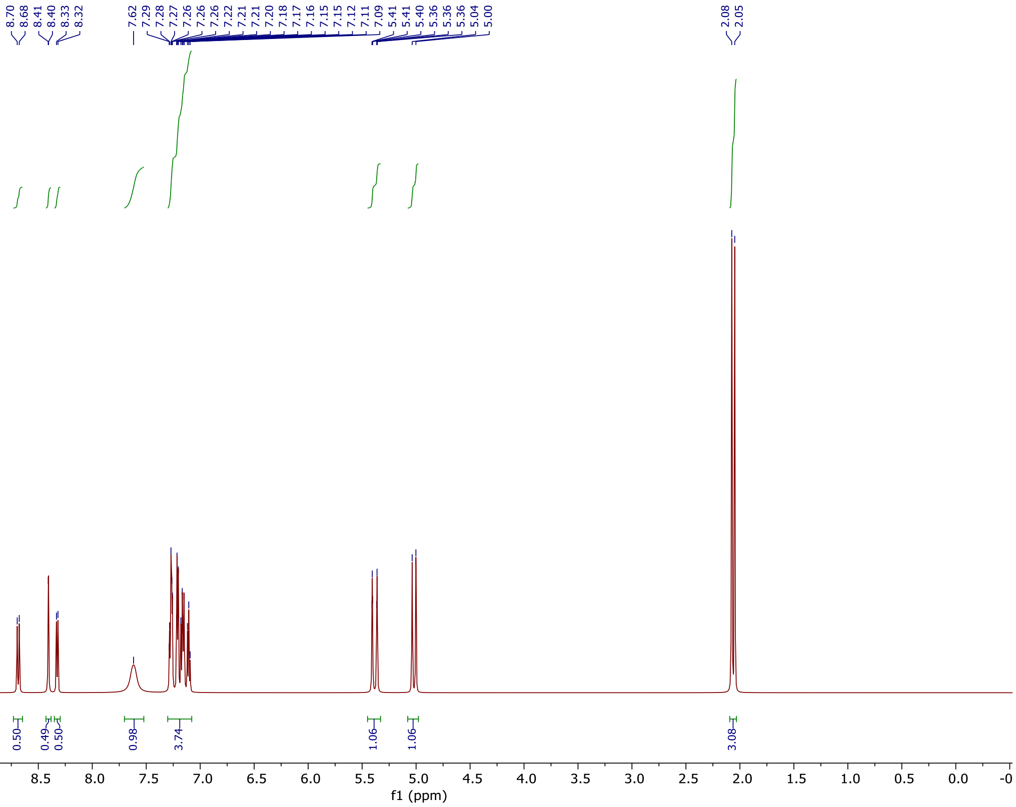

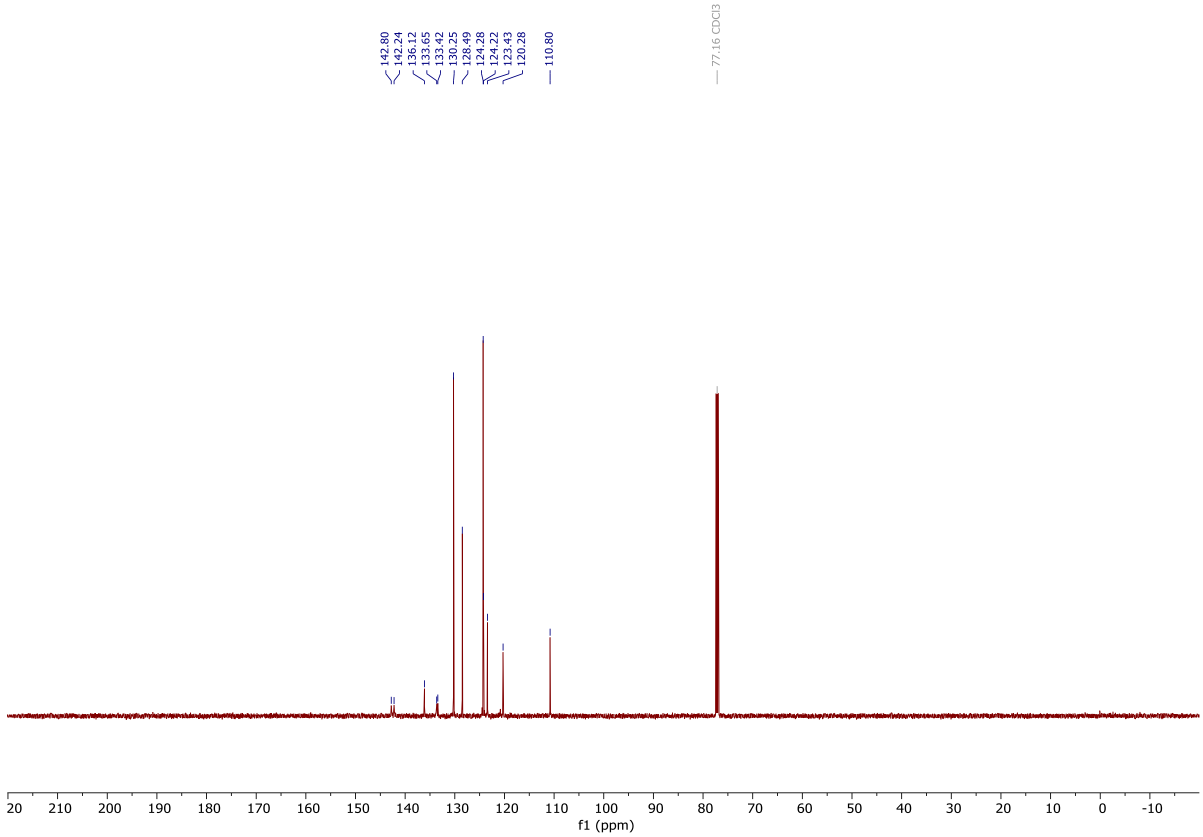

N-(2-(prop-1-en-2-yl) phenyl) formamide (**3a14**)

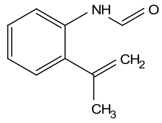

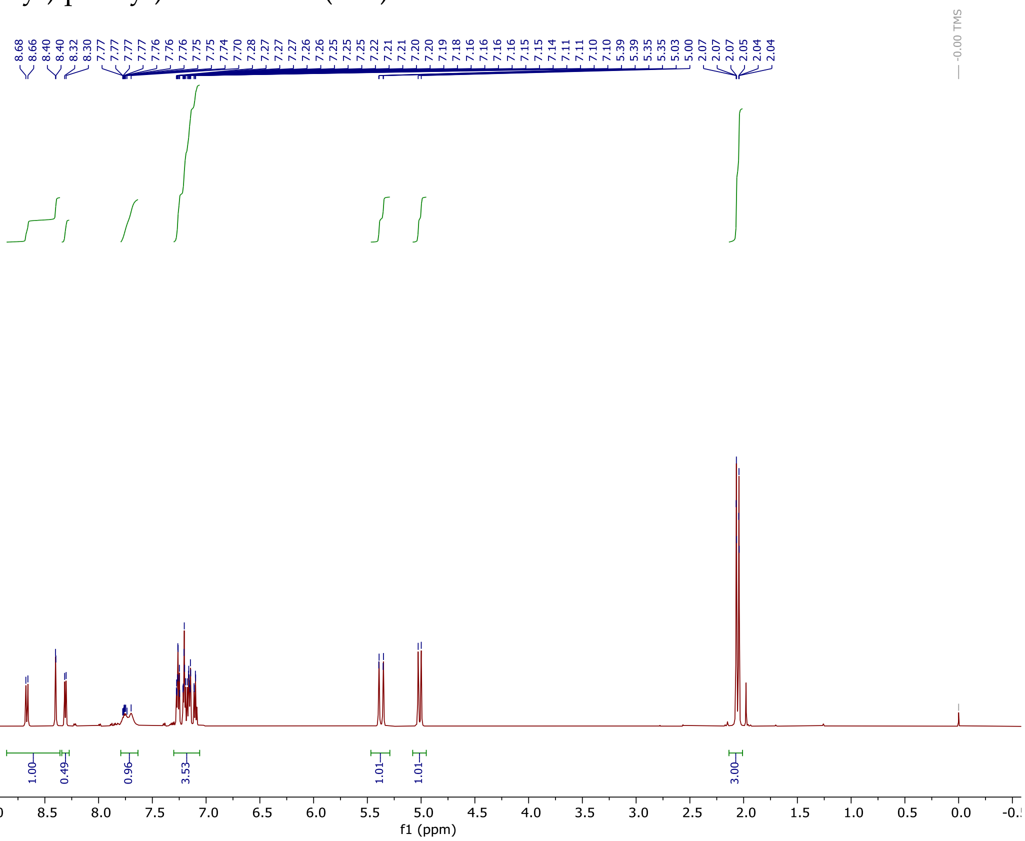

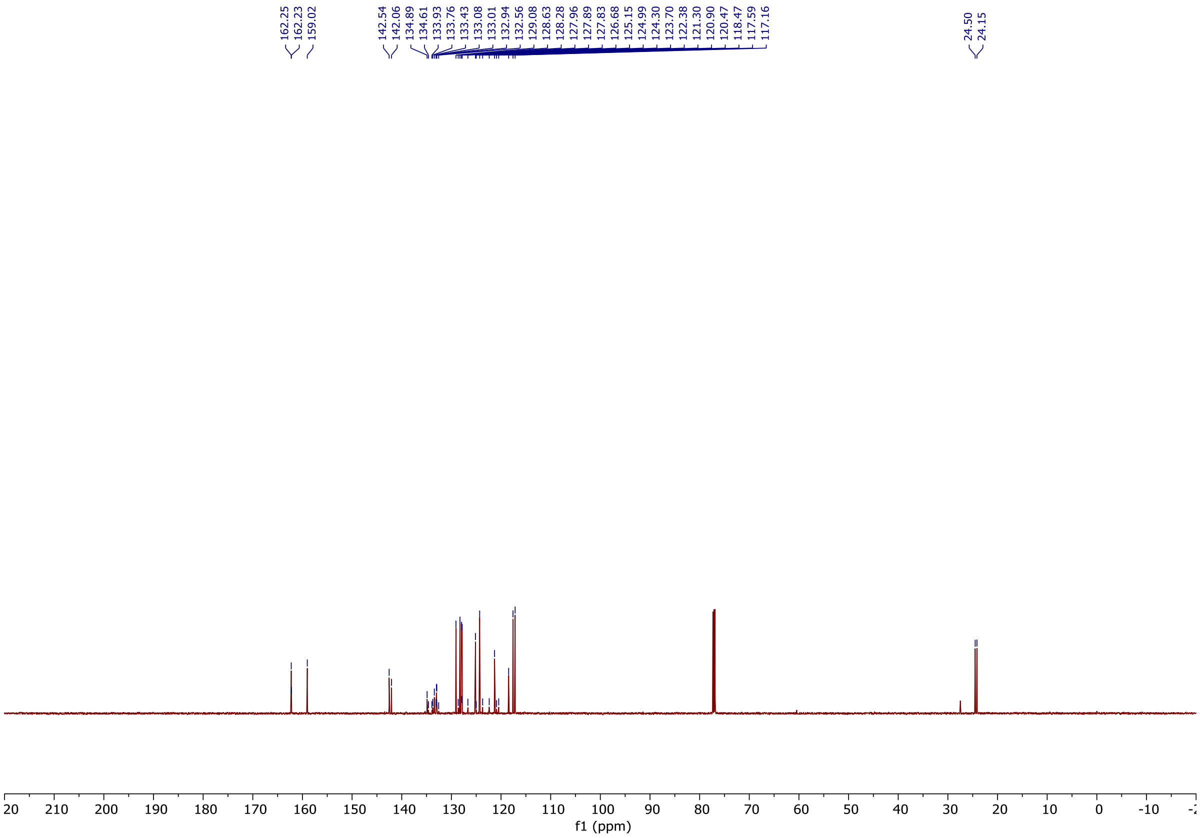

N-(naphthalen-1-yl) formamide (**3a**15)

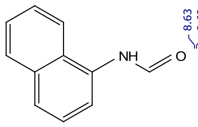

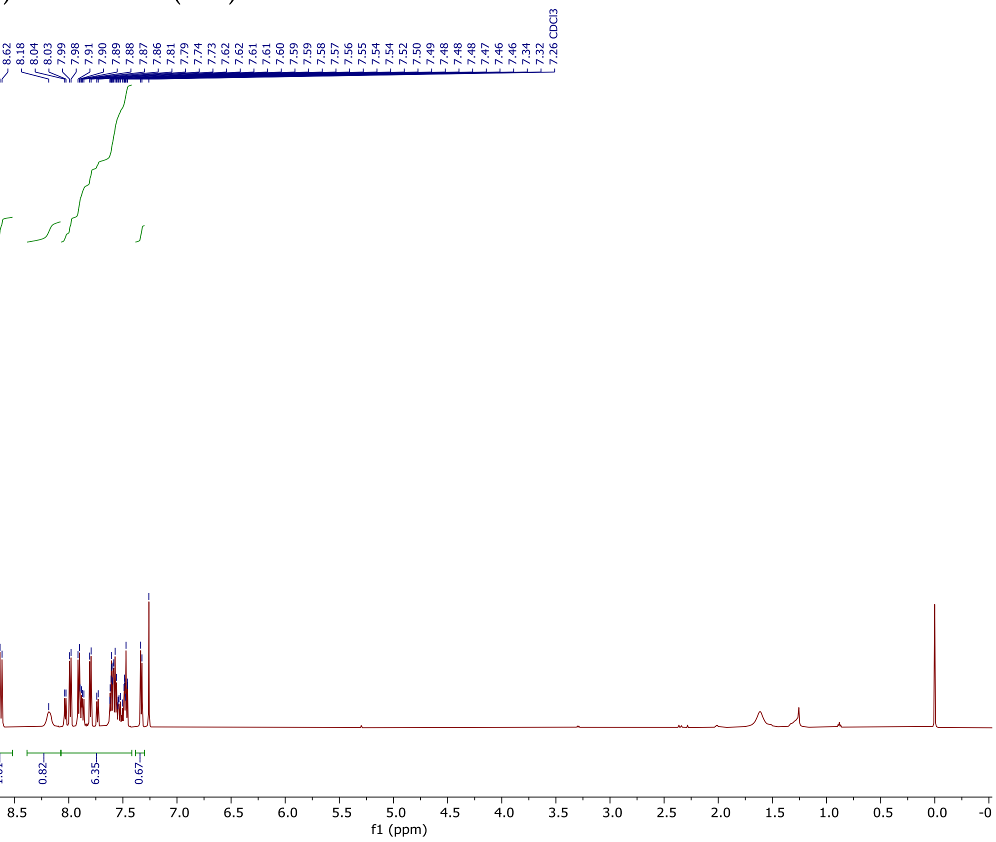

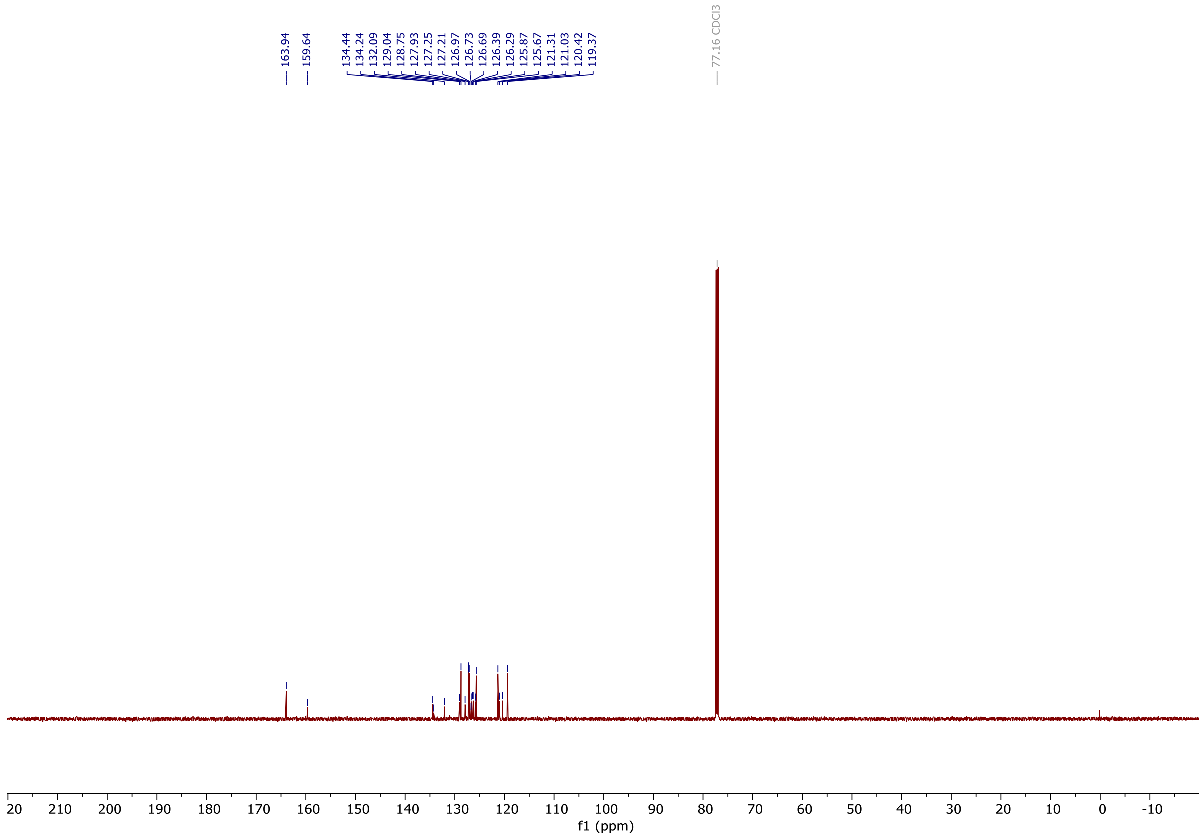

N-(quinolin-3-yl) formamide (**3a16**)

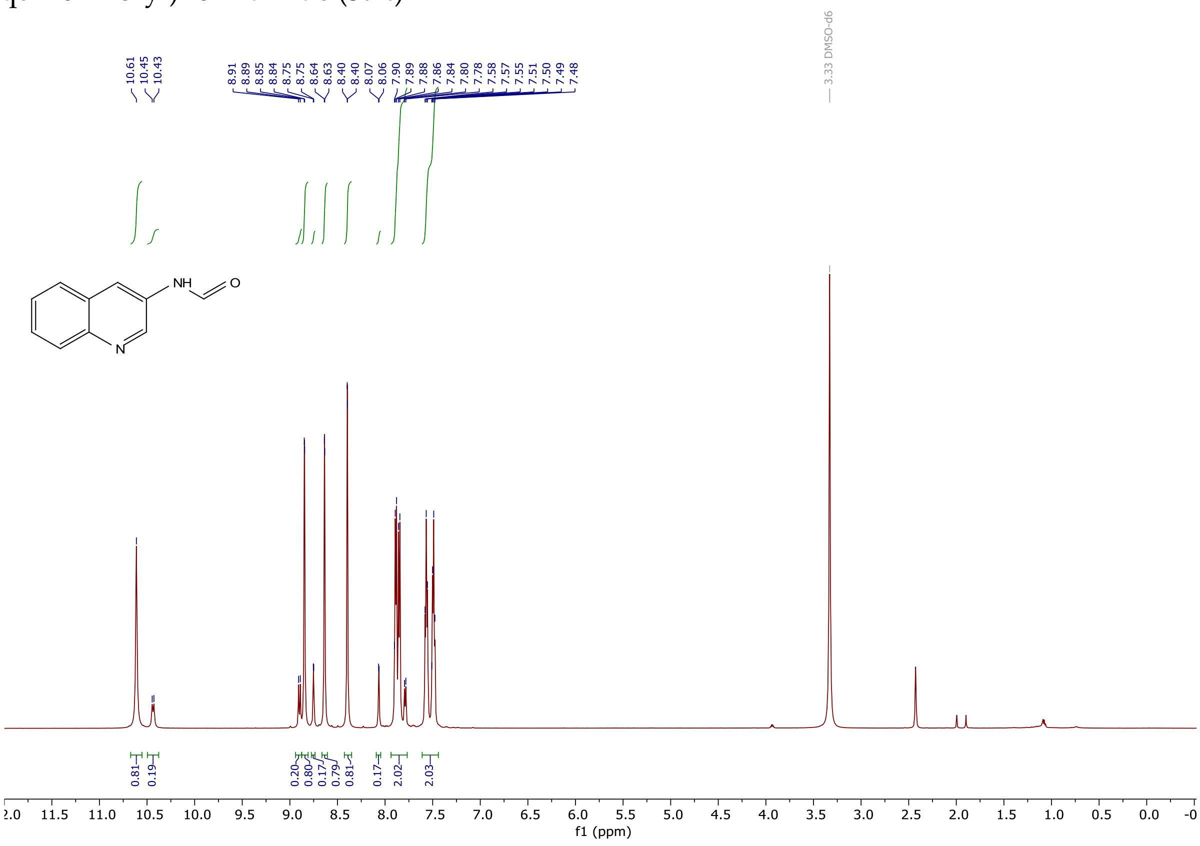

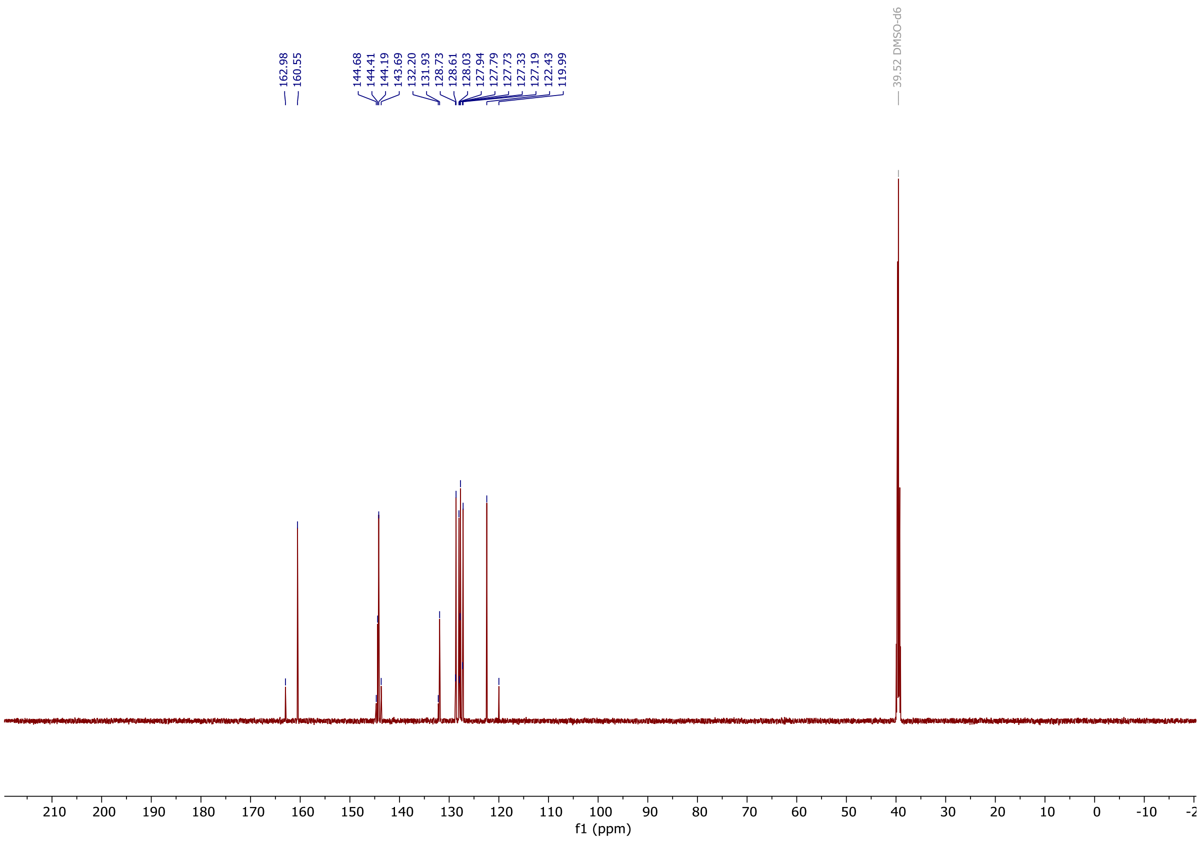

Indoline-1-carbaldehyde (**3a**17)

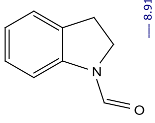

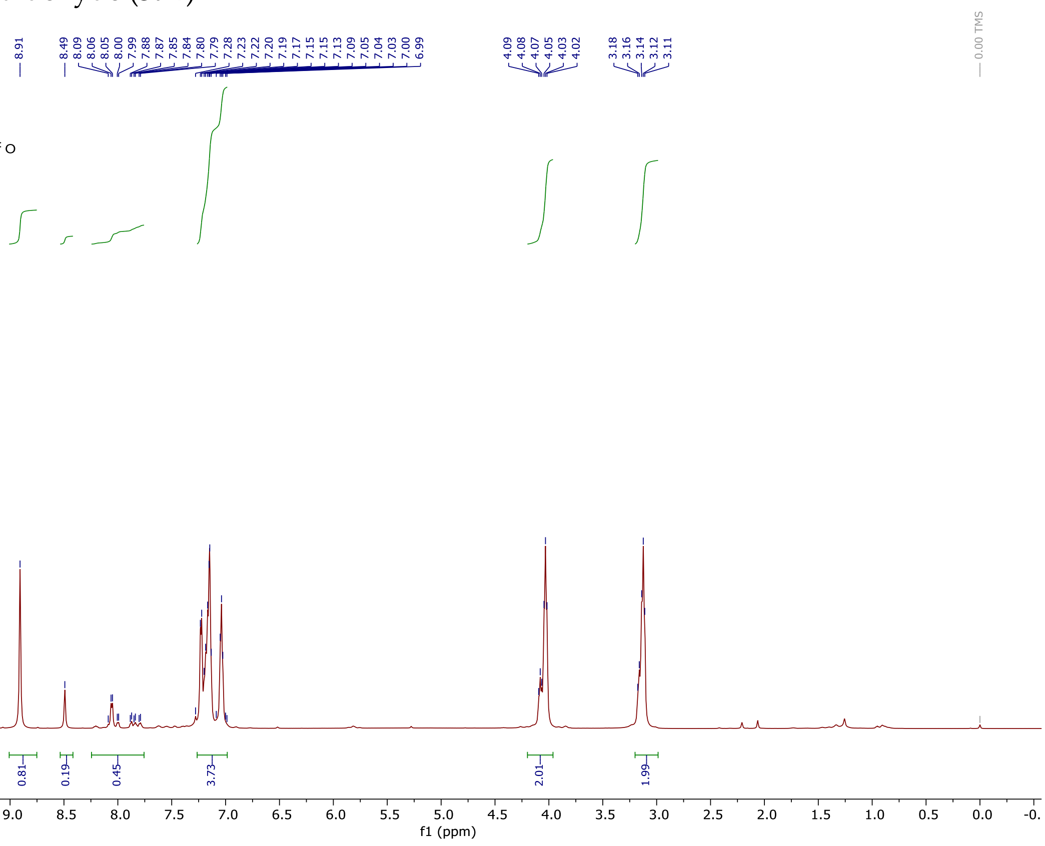

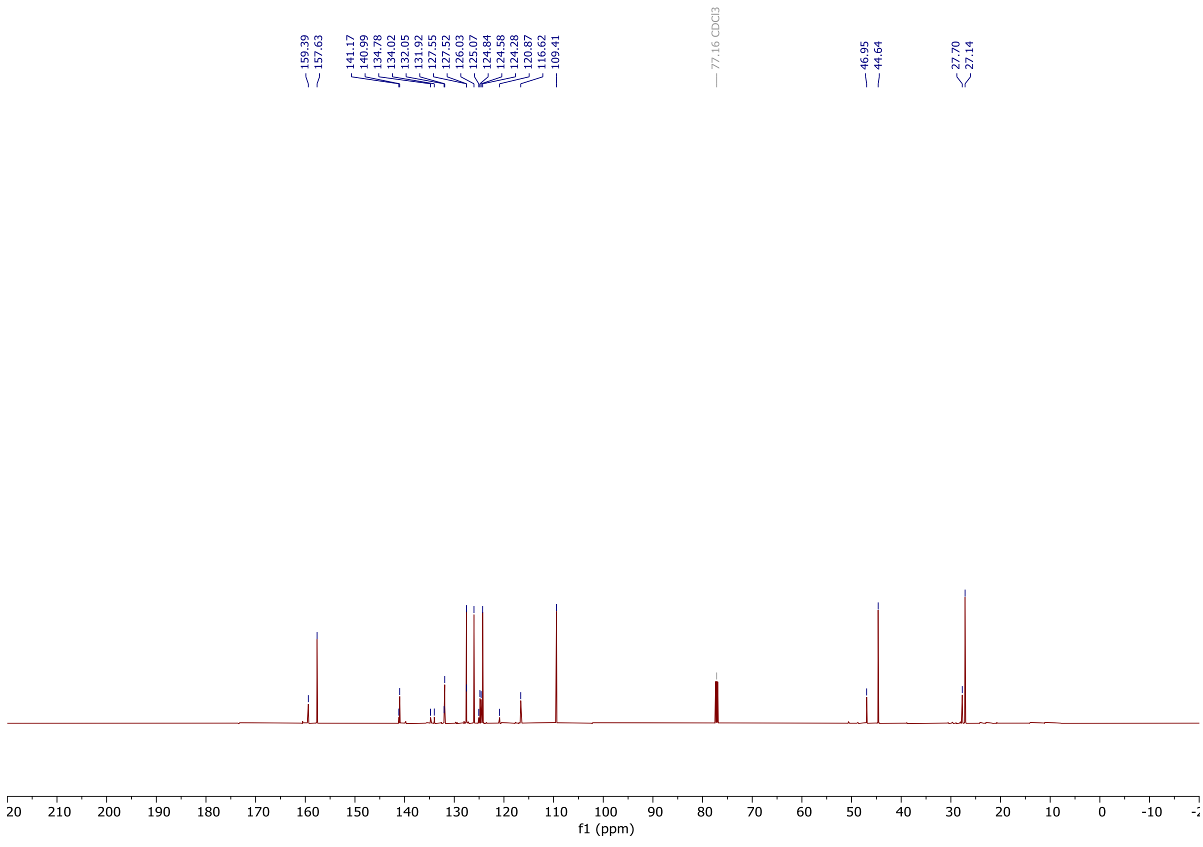

N-benzyl formamide (**3a**18)

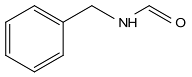

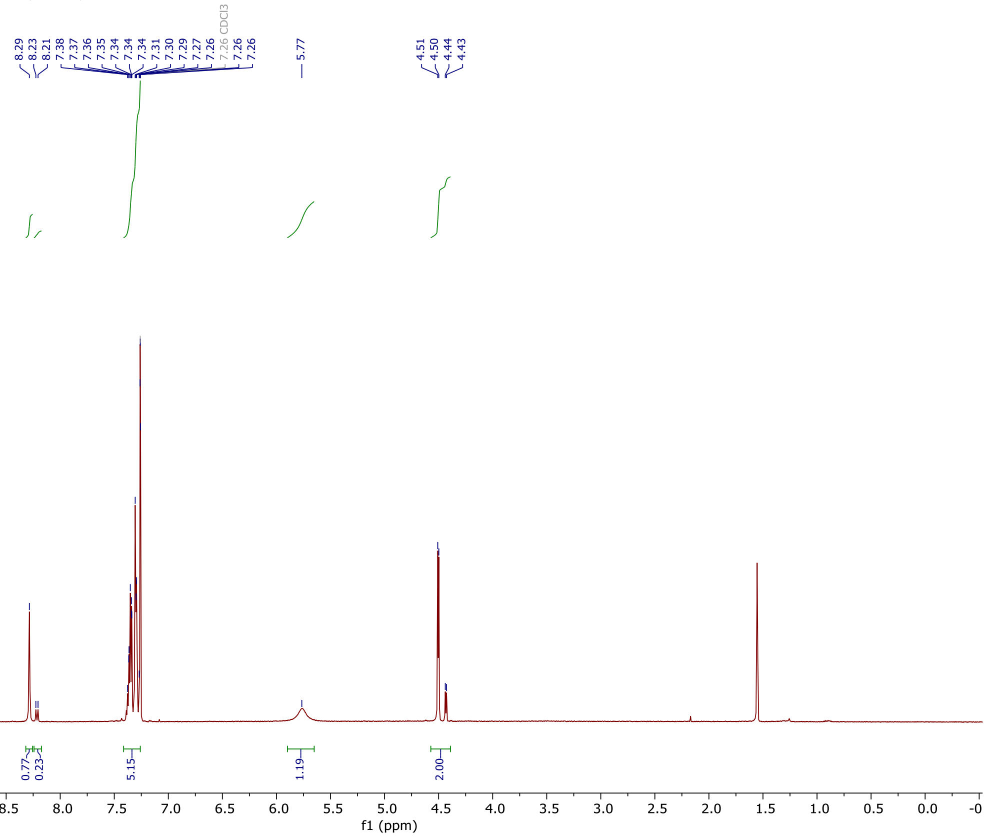

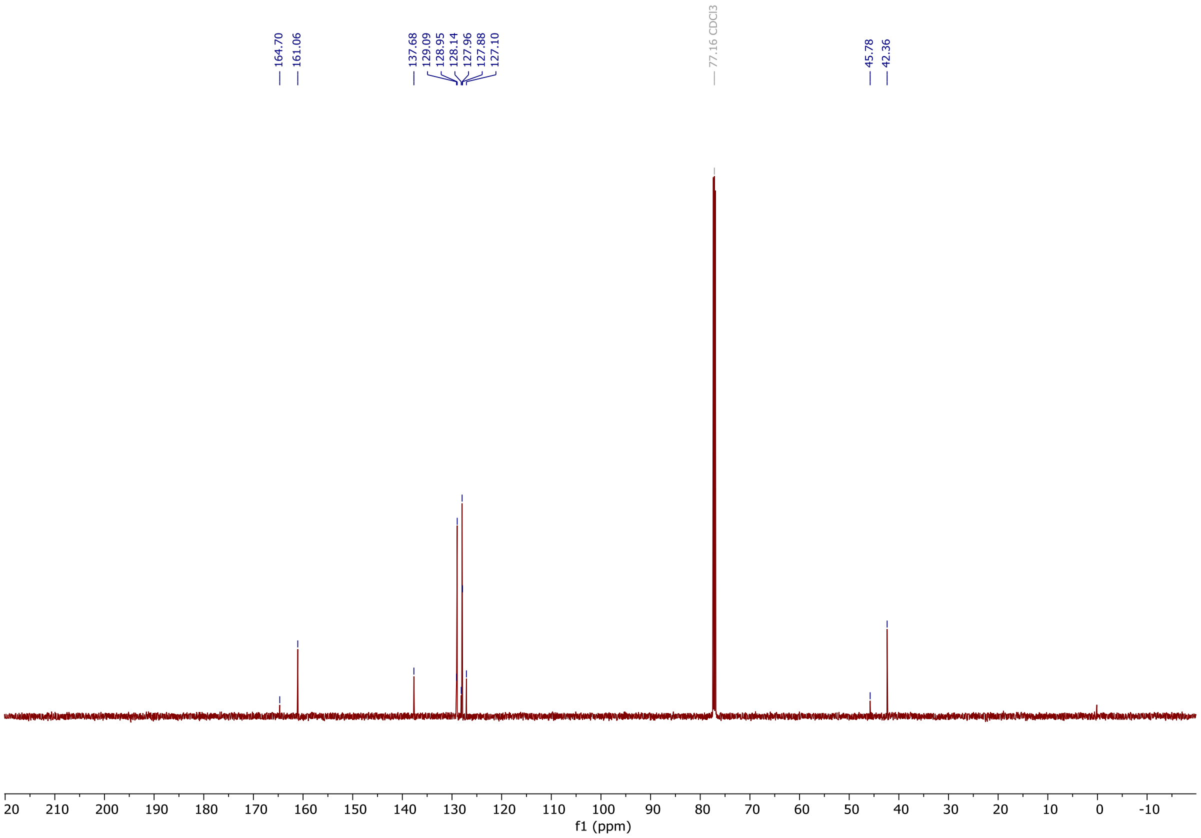

N-(2-phenylethyl) formamide (**3a**19)

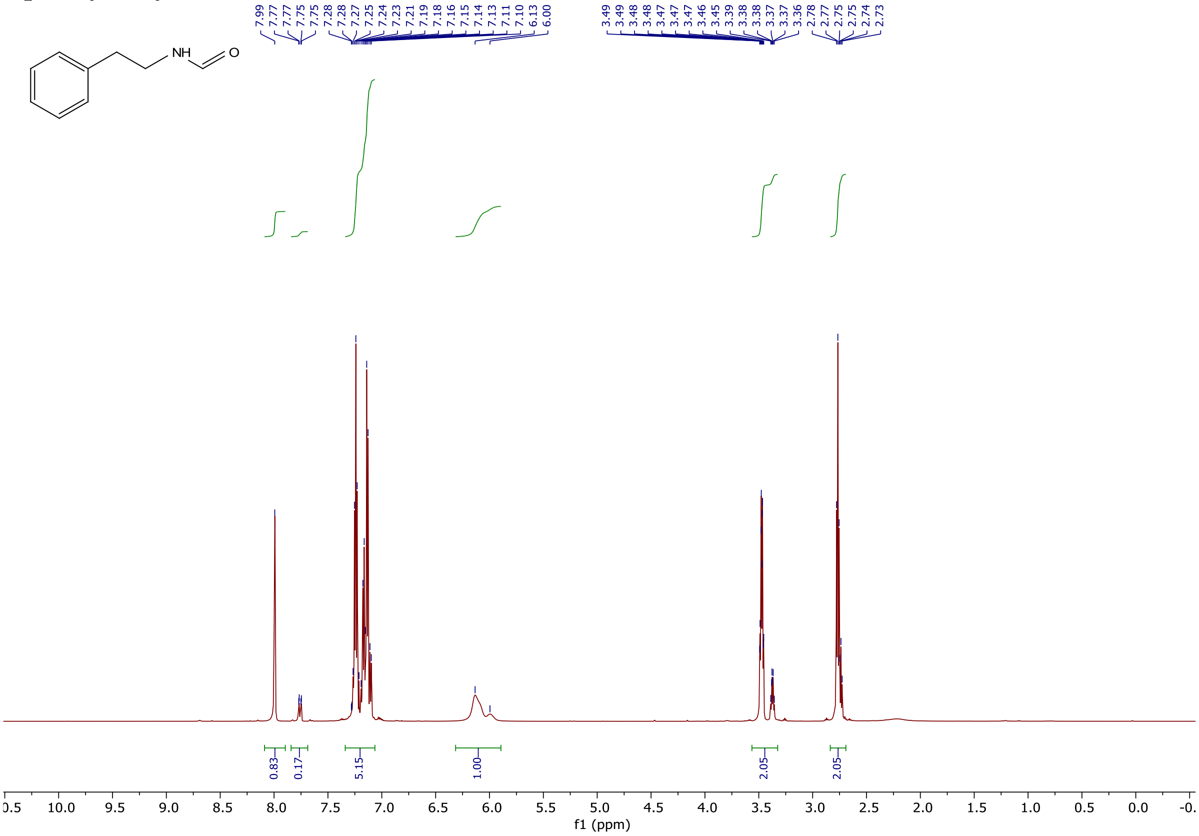

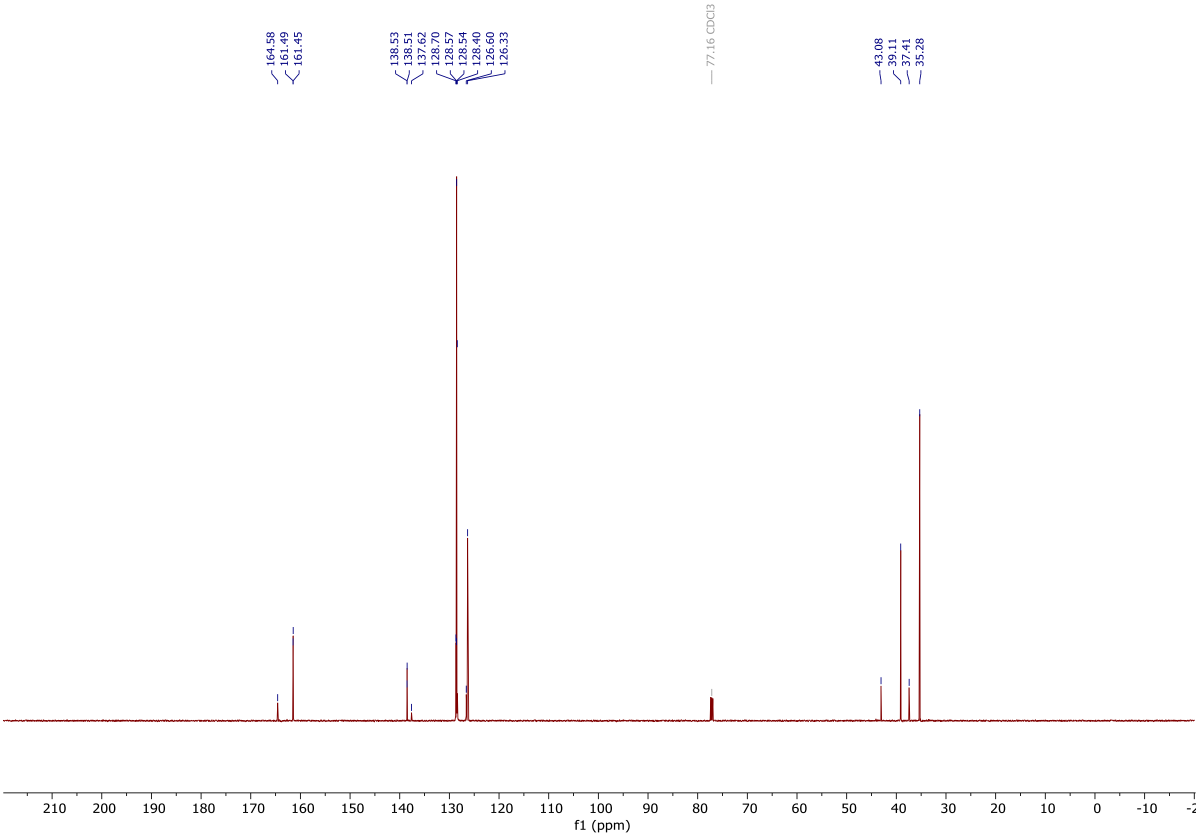

N-benzhydrylformamide (**3a**20)

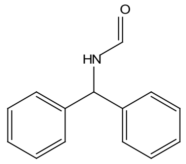

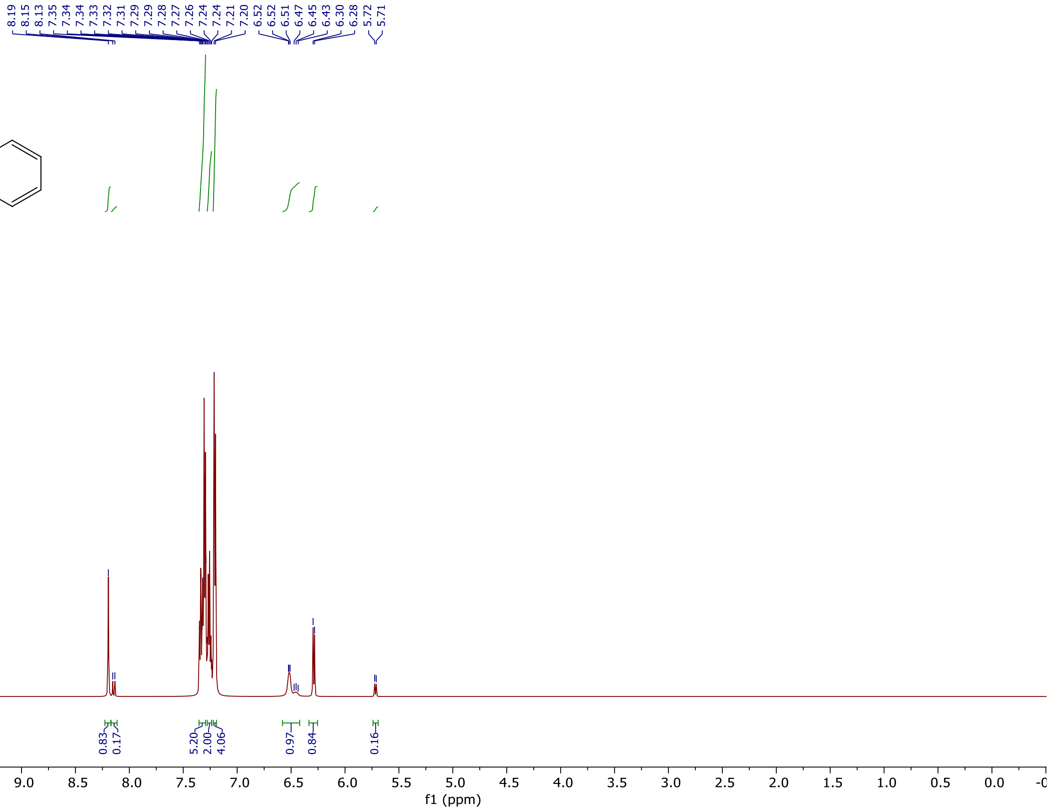

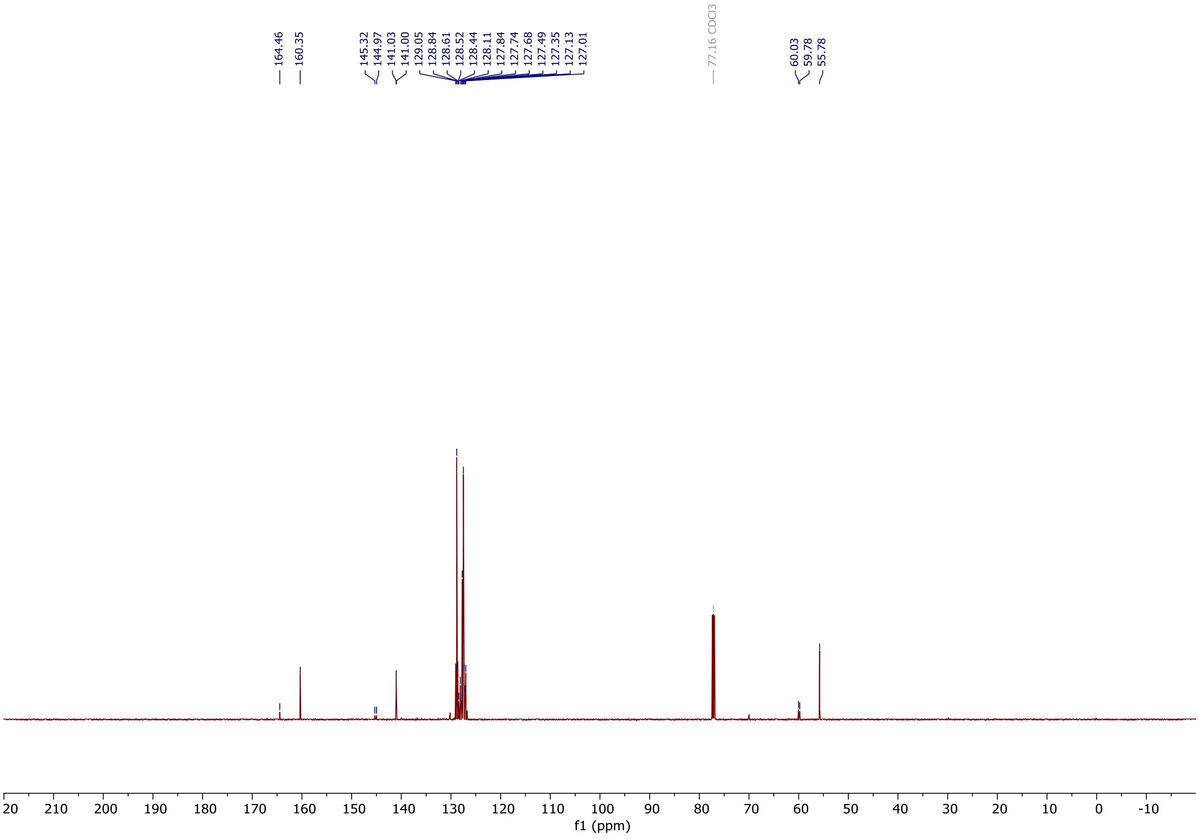

N-(furan-2-ylmethyl) formamide (**3a**21)

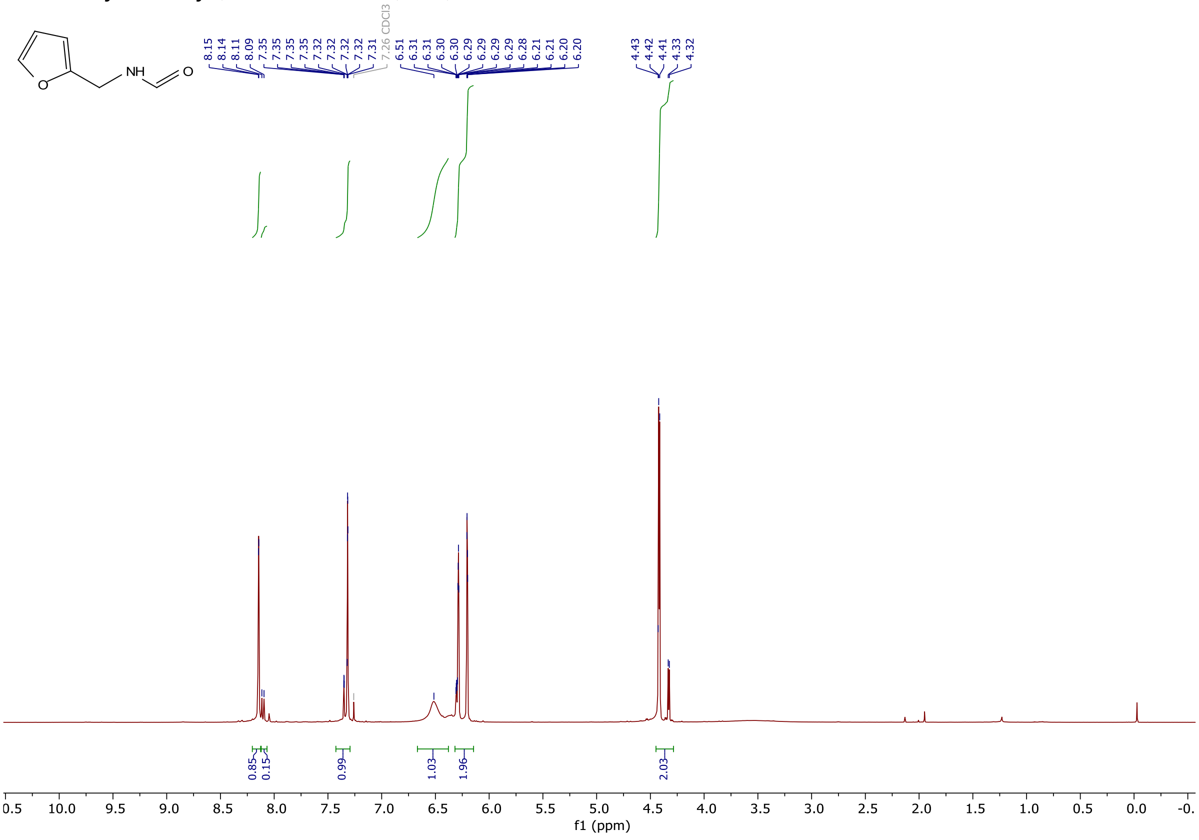

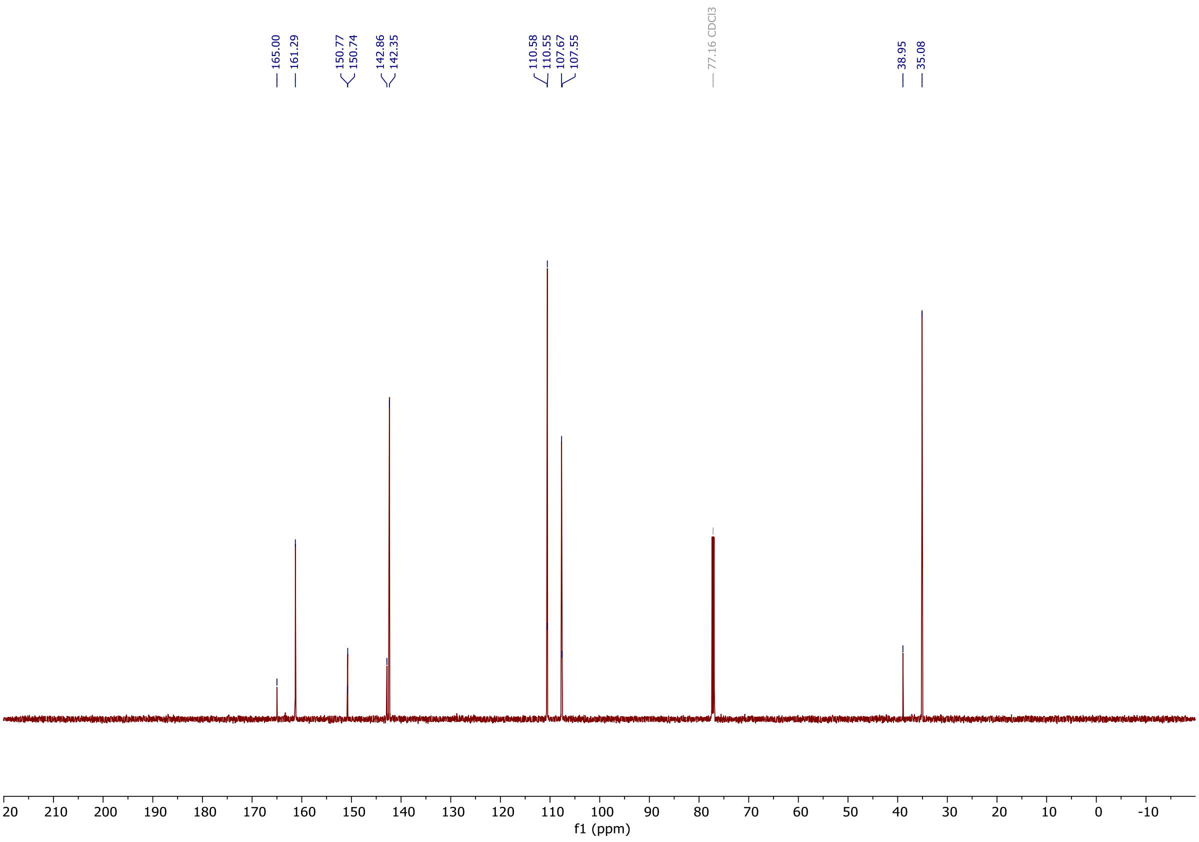

N-methyl-*N*-phenylformamide (**3a**22)

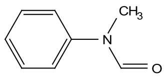

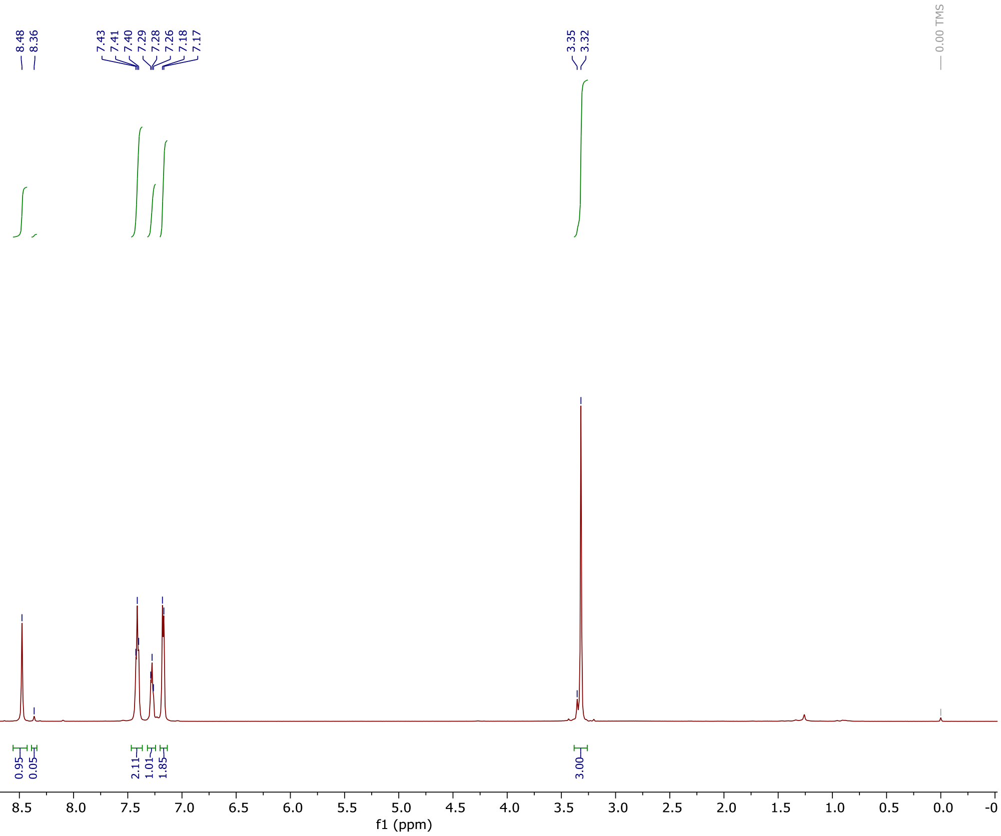

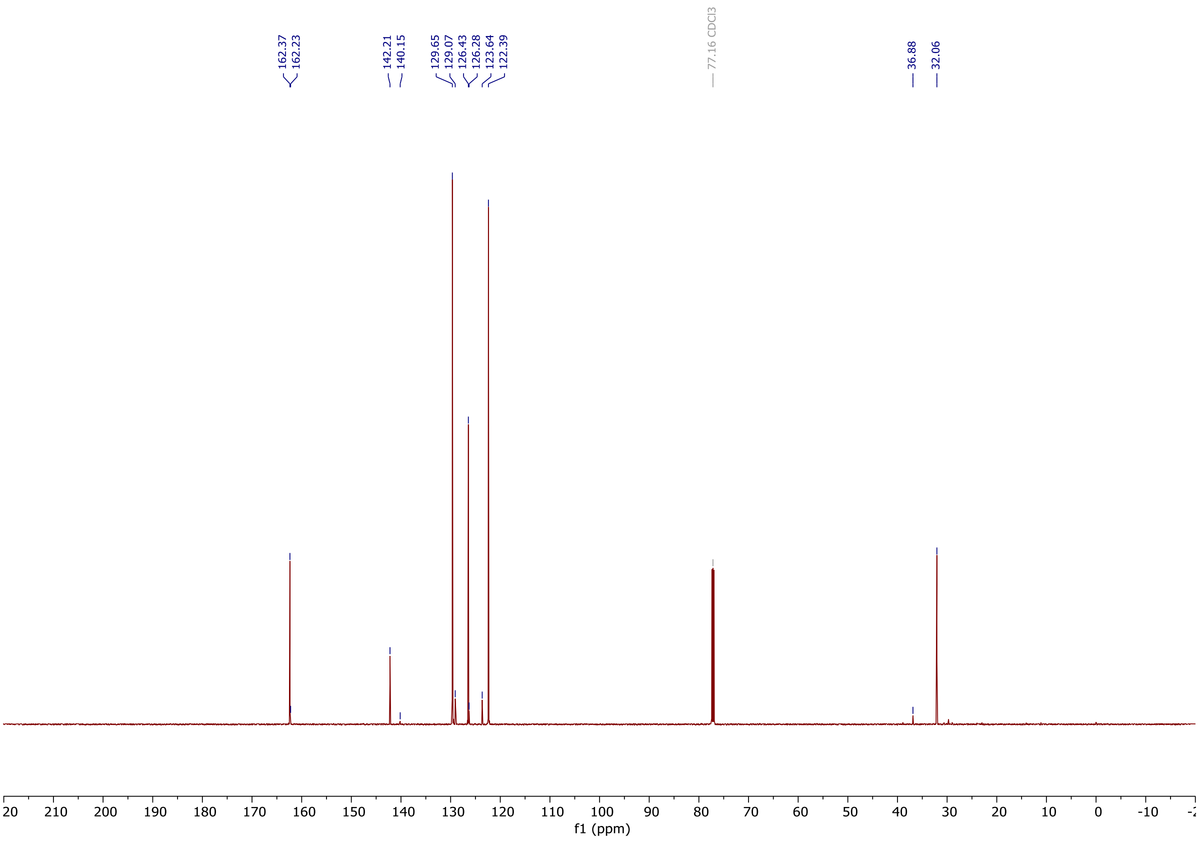

N-benzyl-*N*-methylformamide (**3a**23)

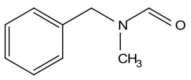

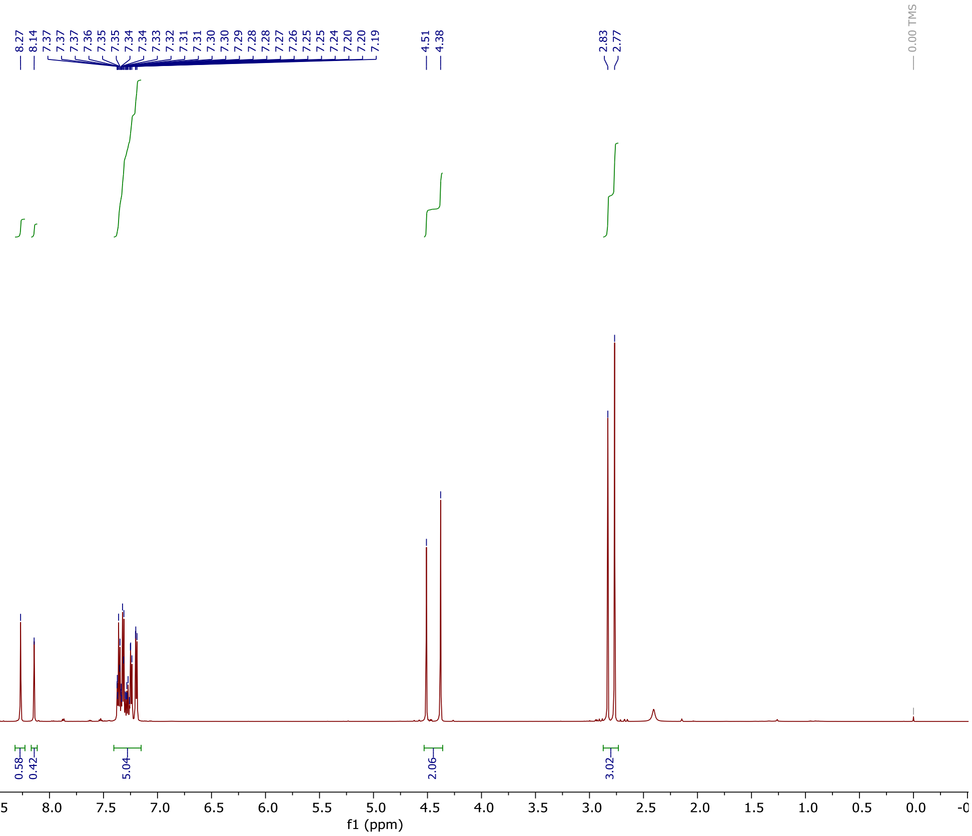

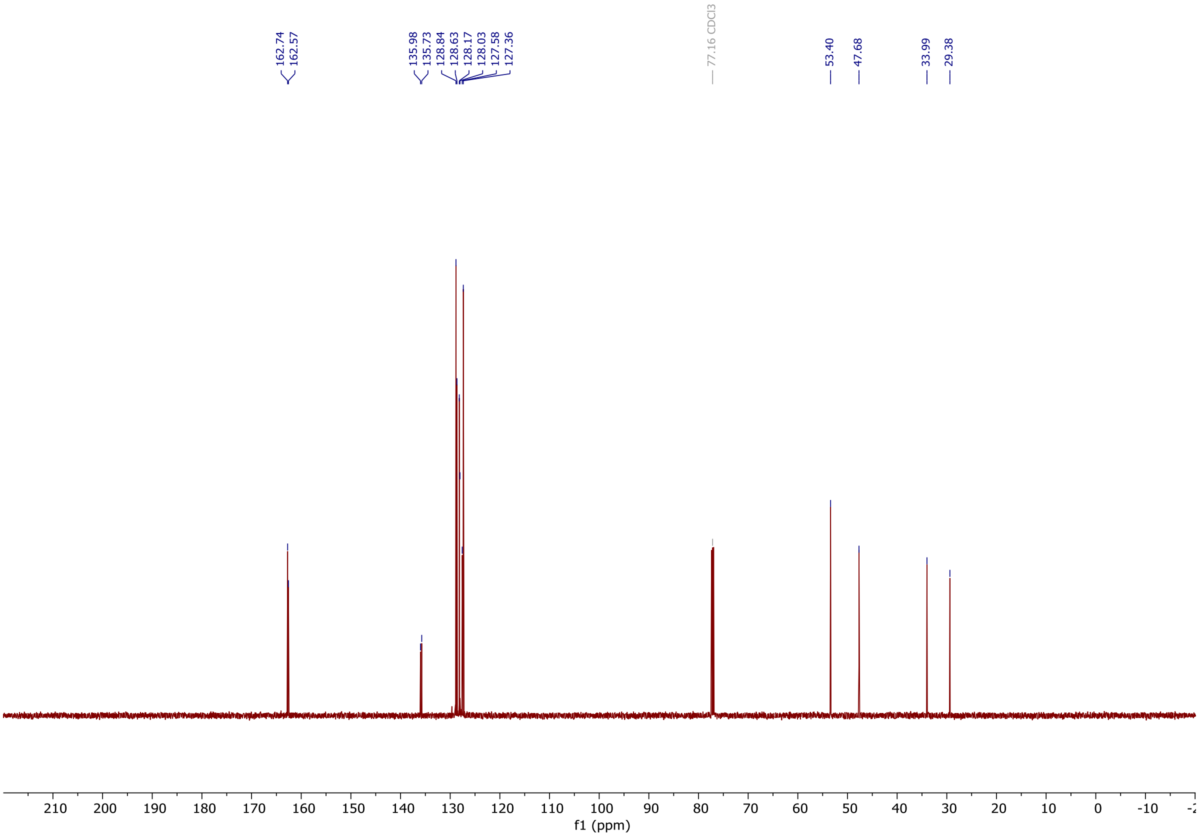

N-methyl-*N*-phenethylformamide (**3a**24)

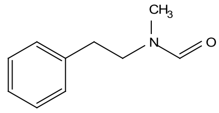

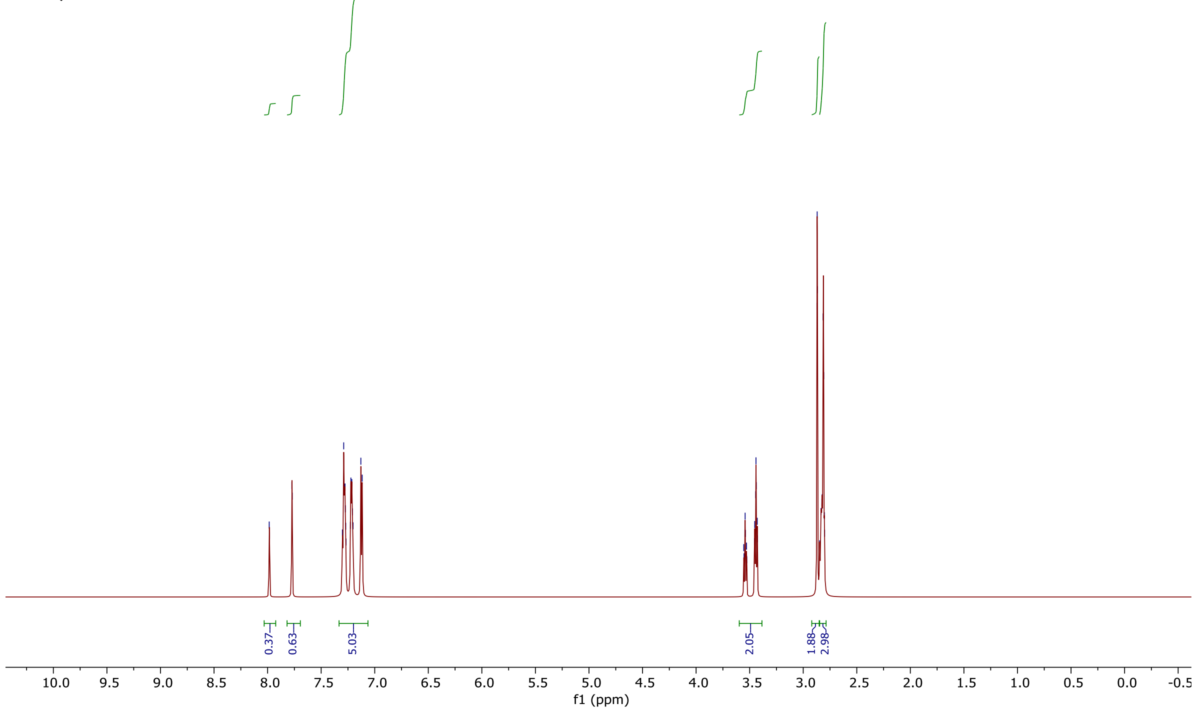

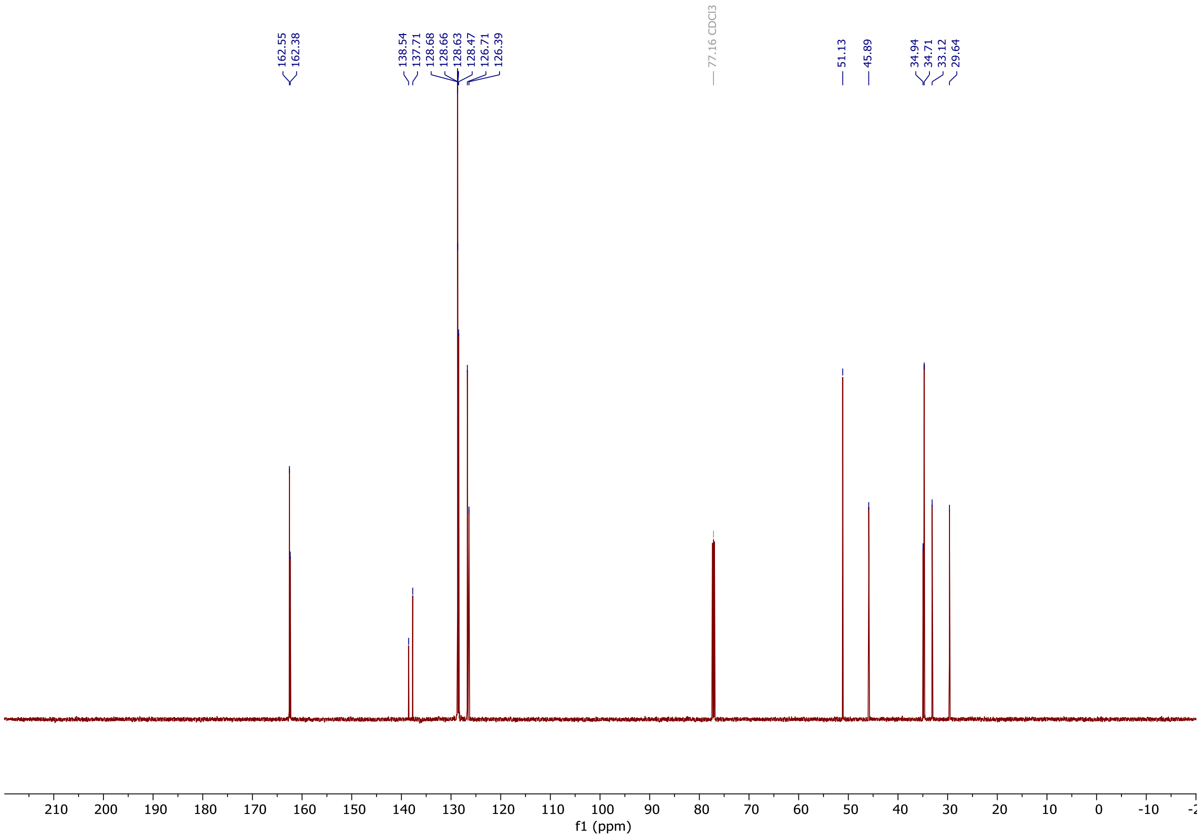

3,4-dihydroisoquinoline-2(1H)-carbaldehyde (**3a25**)

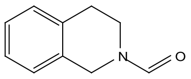

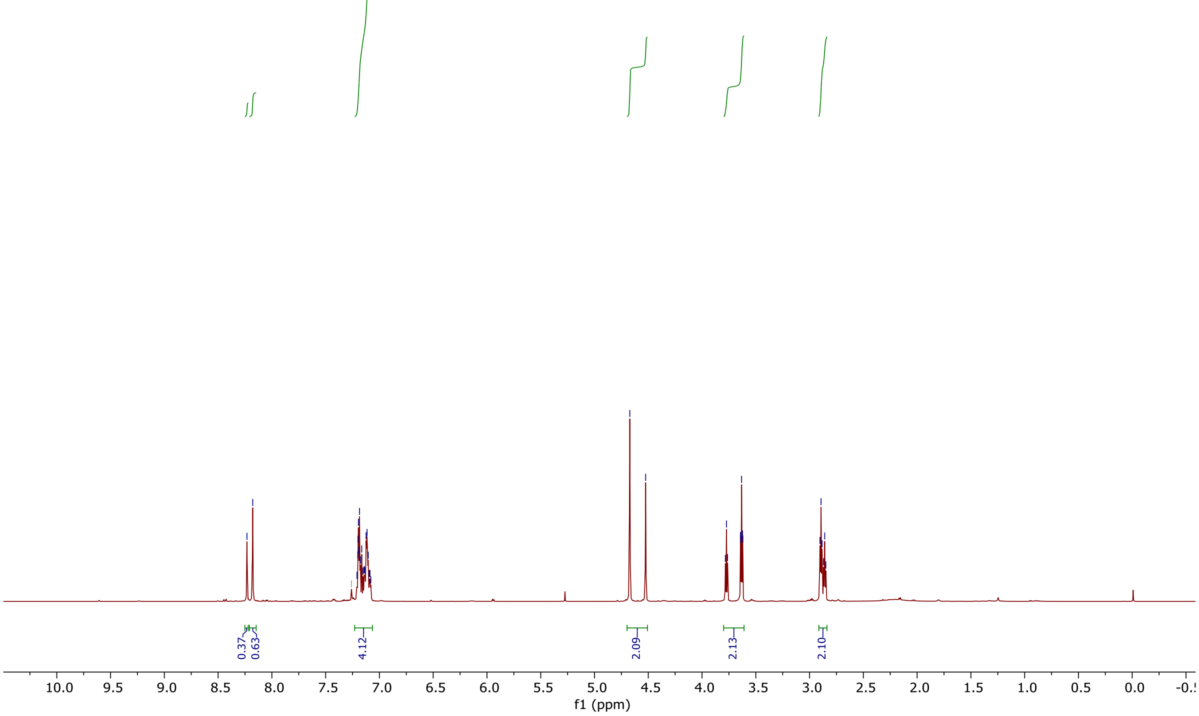

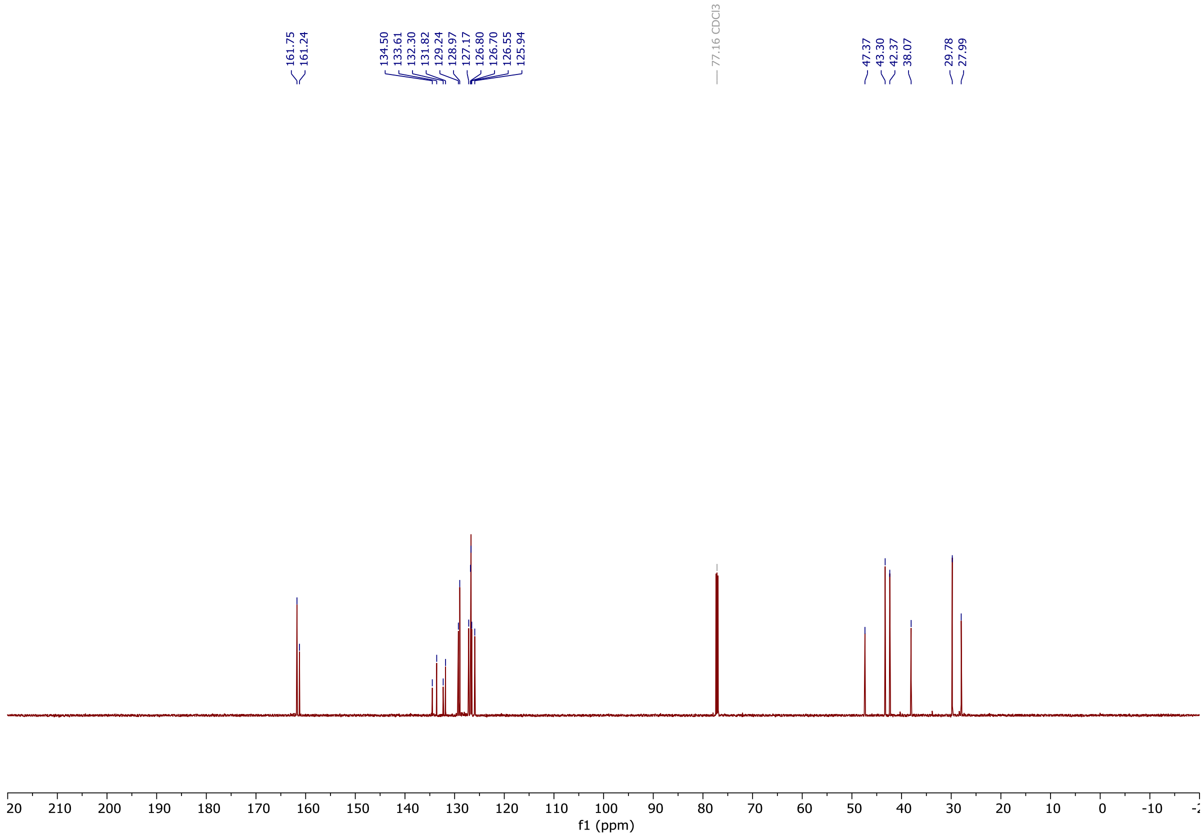

N-benzyl-*N*-phenethylformamide (**3a**26)

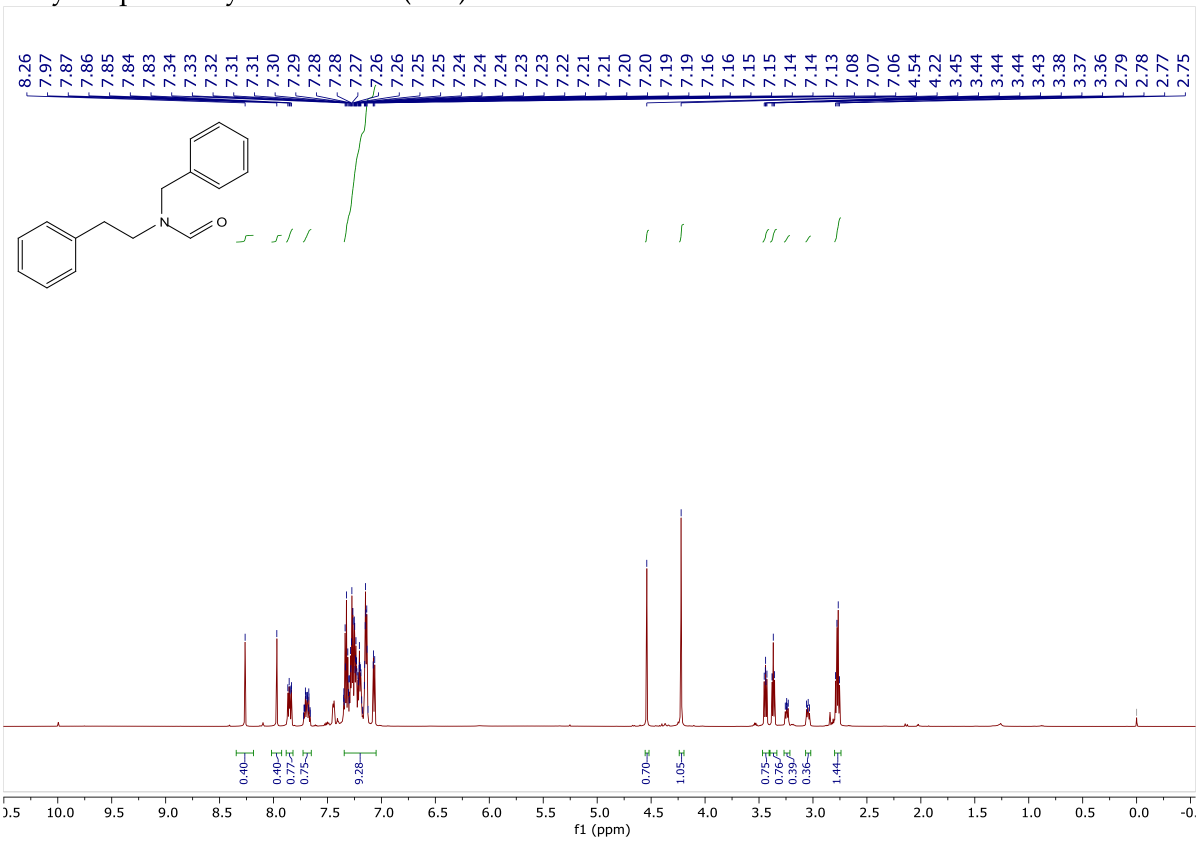

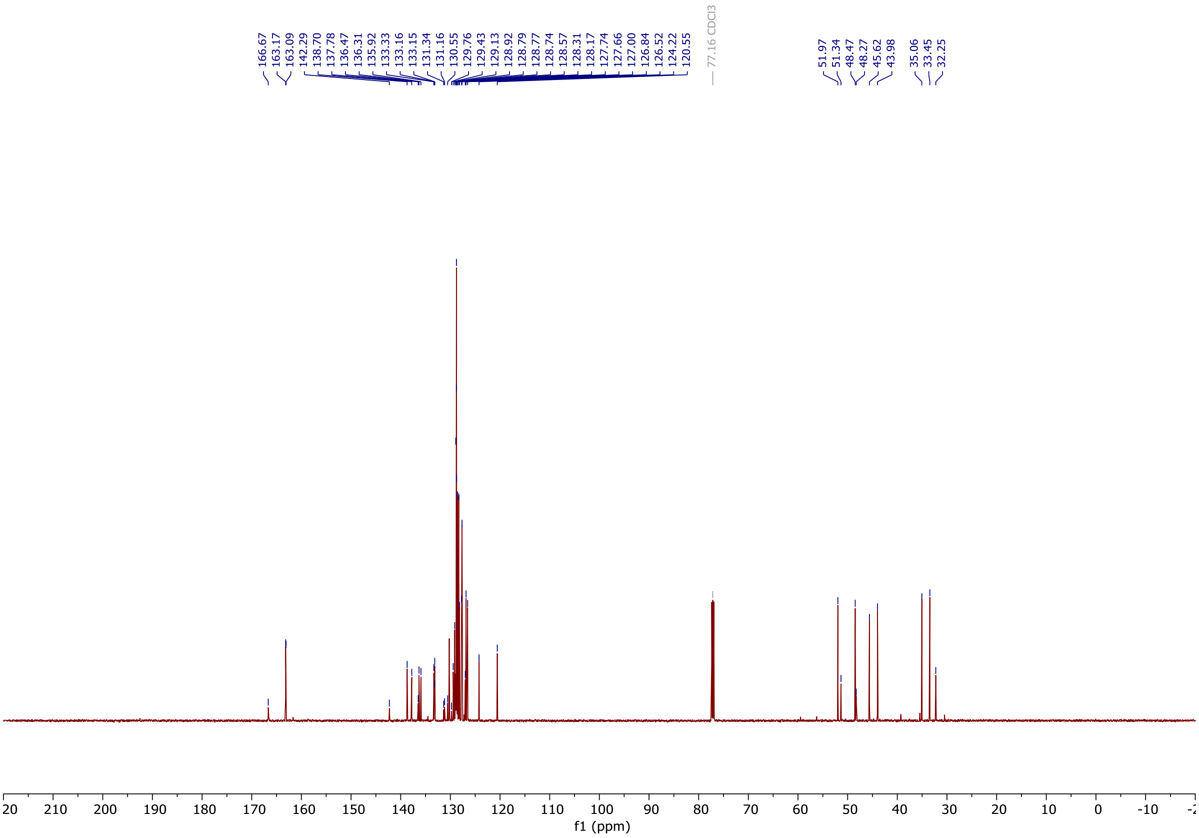

N-heptylformamide (**3a**27)

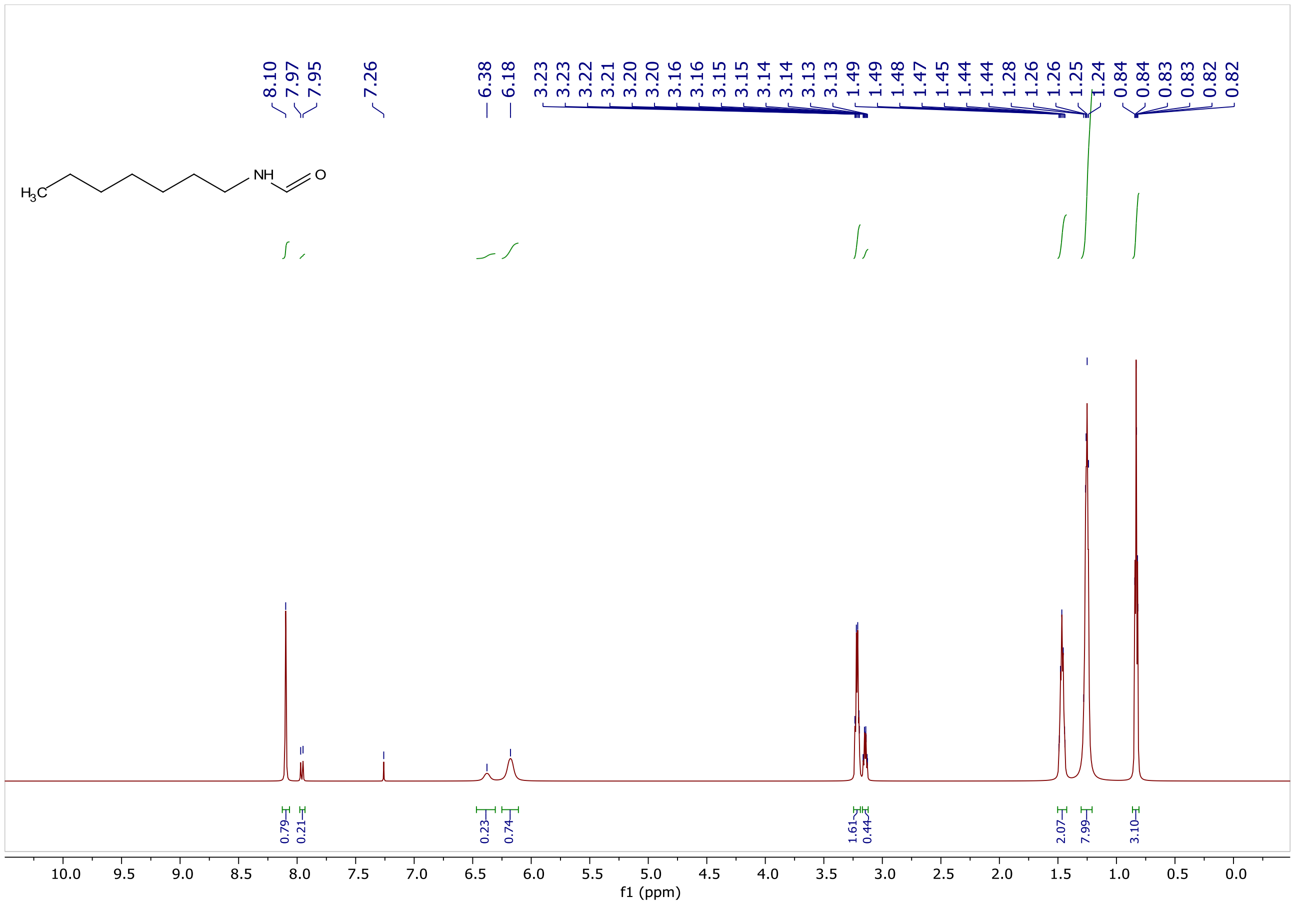

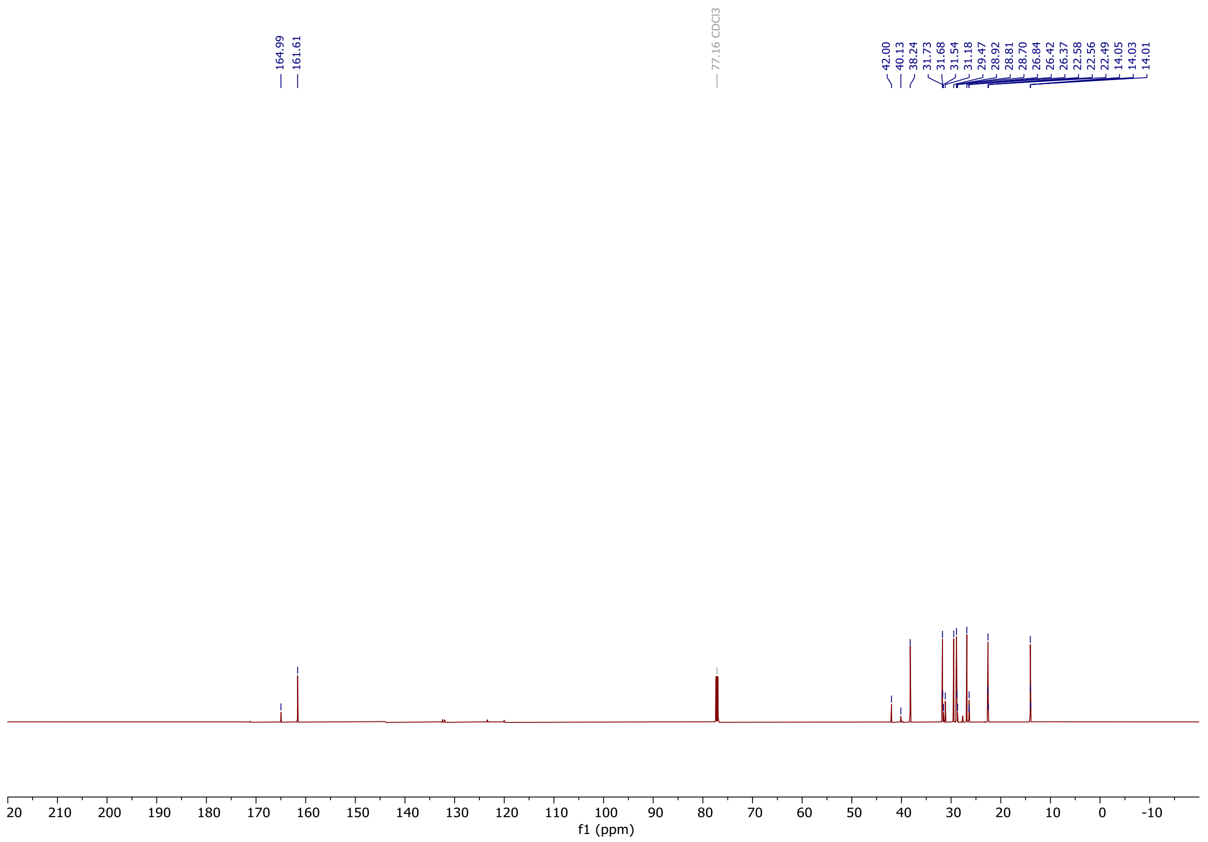

N-octyl formamide (**3a**28)

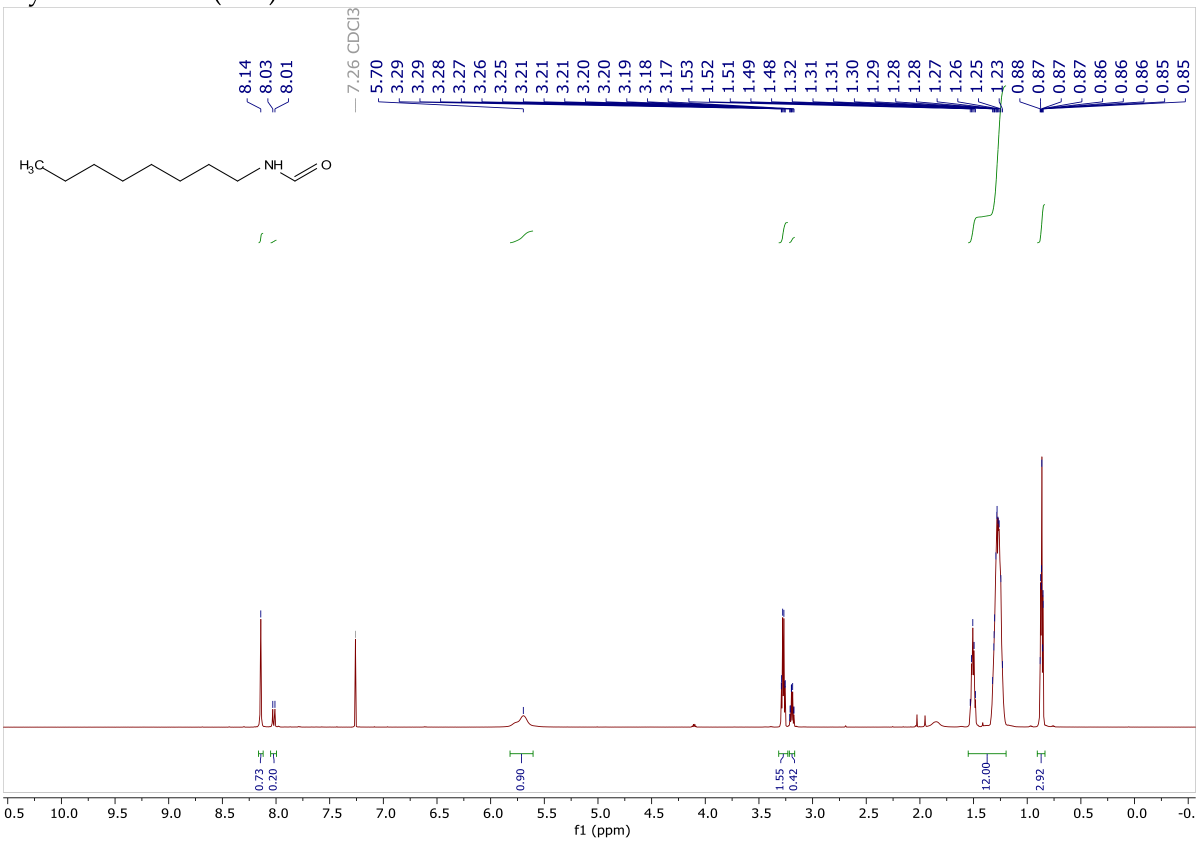

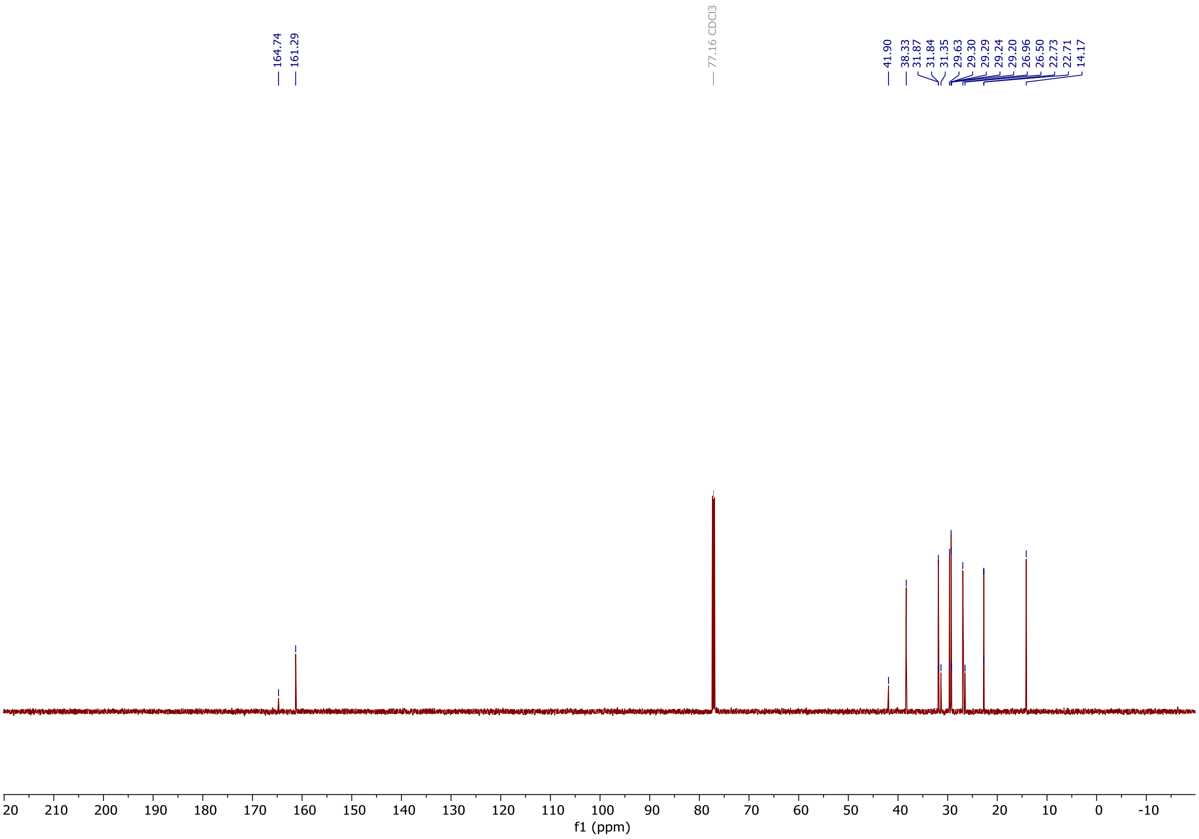

(±)-*N*-(heptan-2-yl) formamide (**3a**[29])

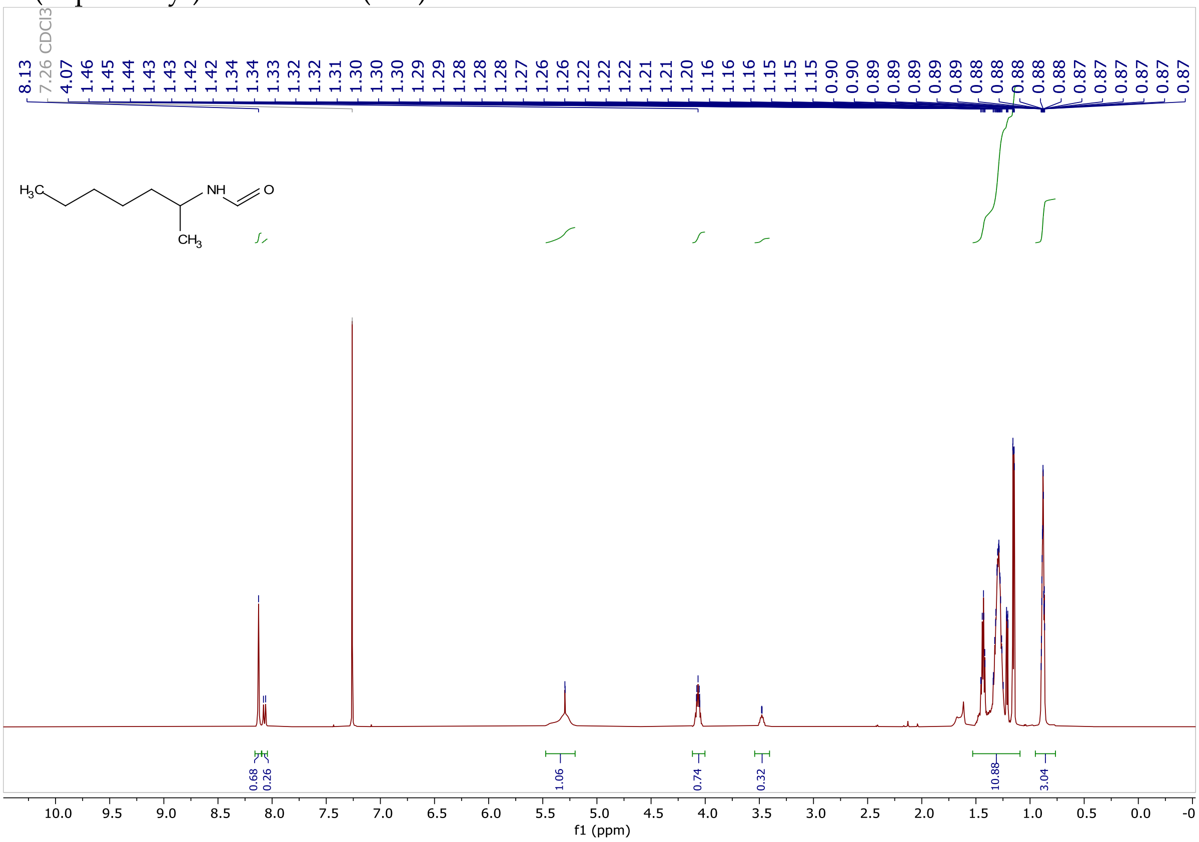

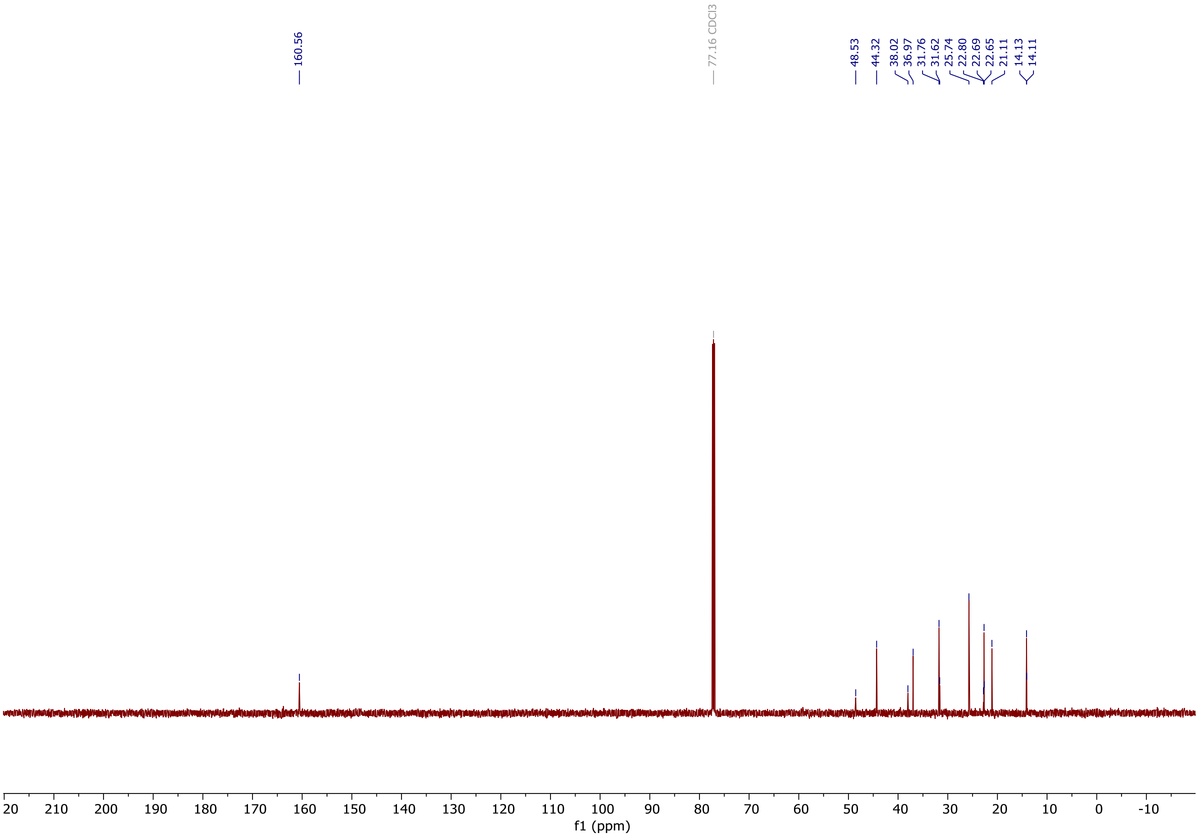

N-allyl-*N*-methylformamide (**3a**30)

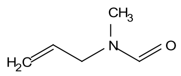

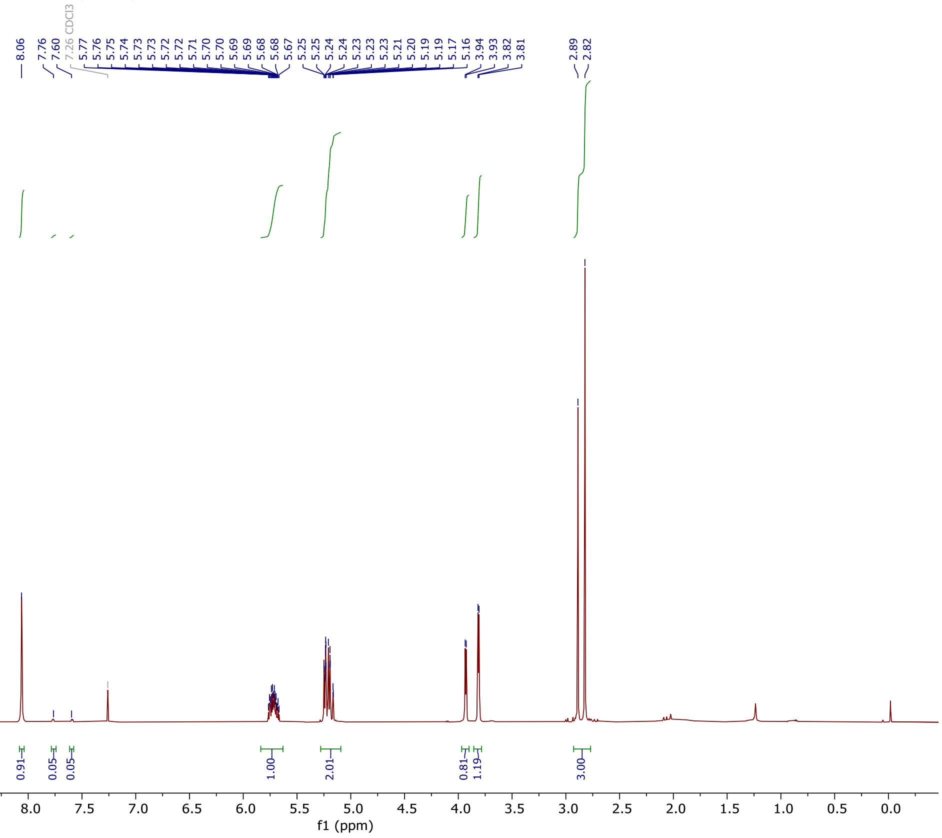

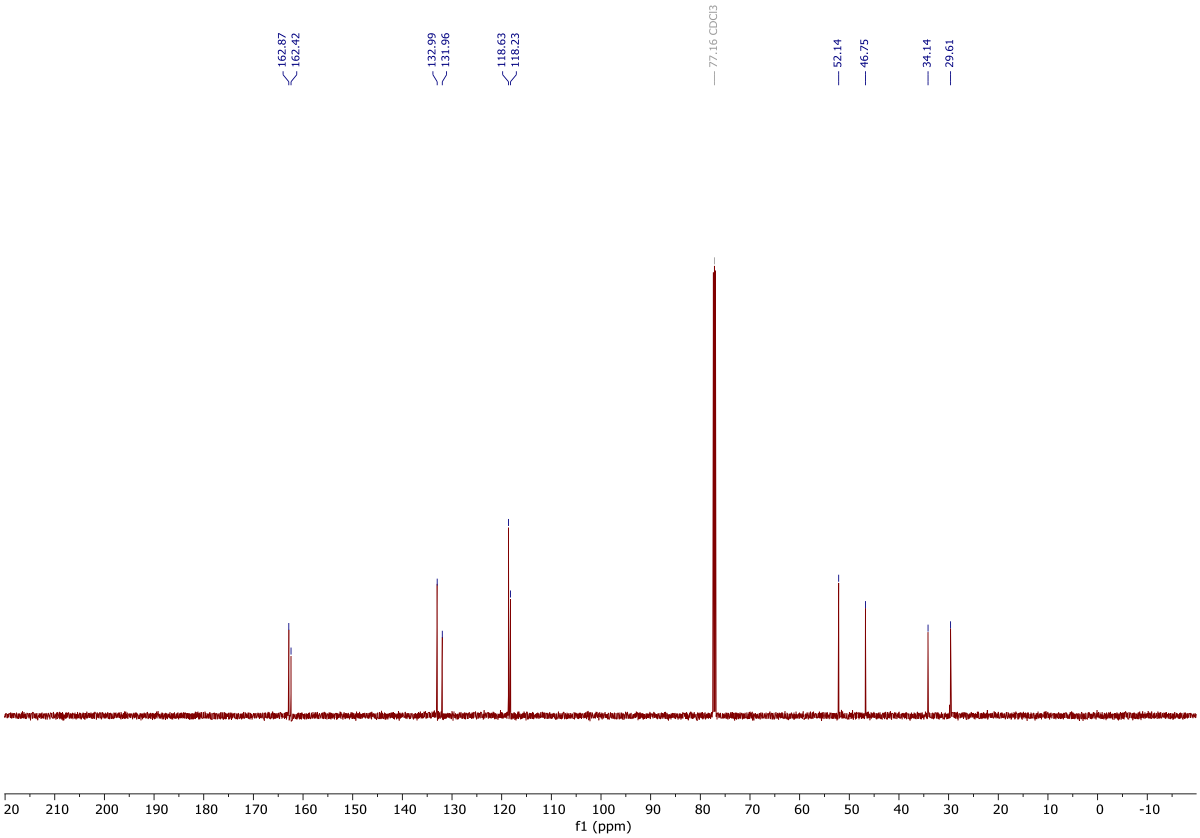

N-cyclohexylformamide (**3a**31)

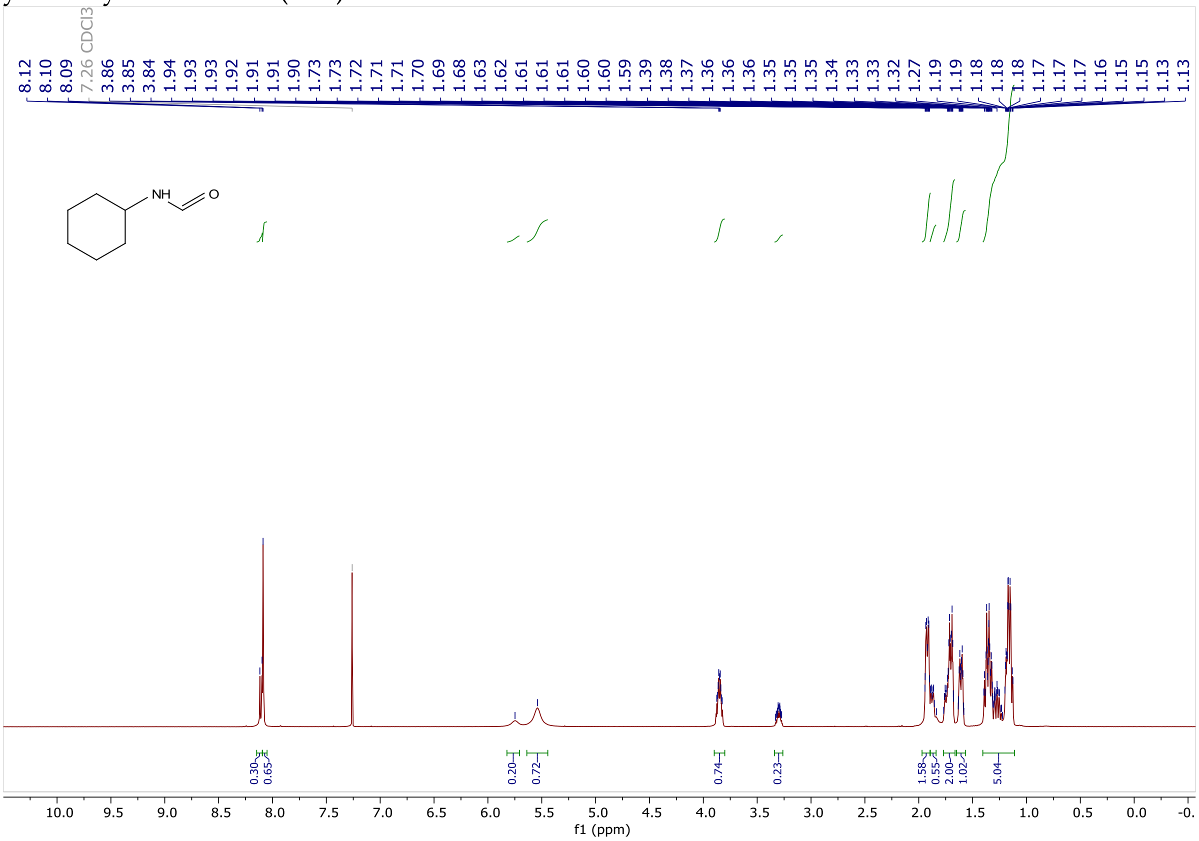

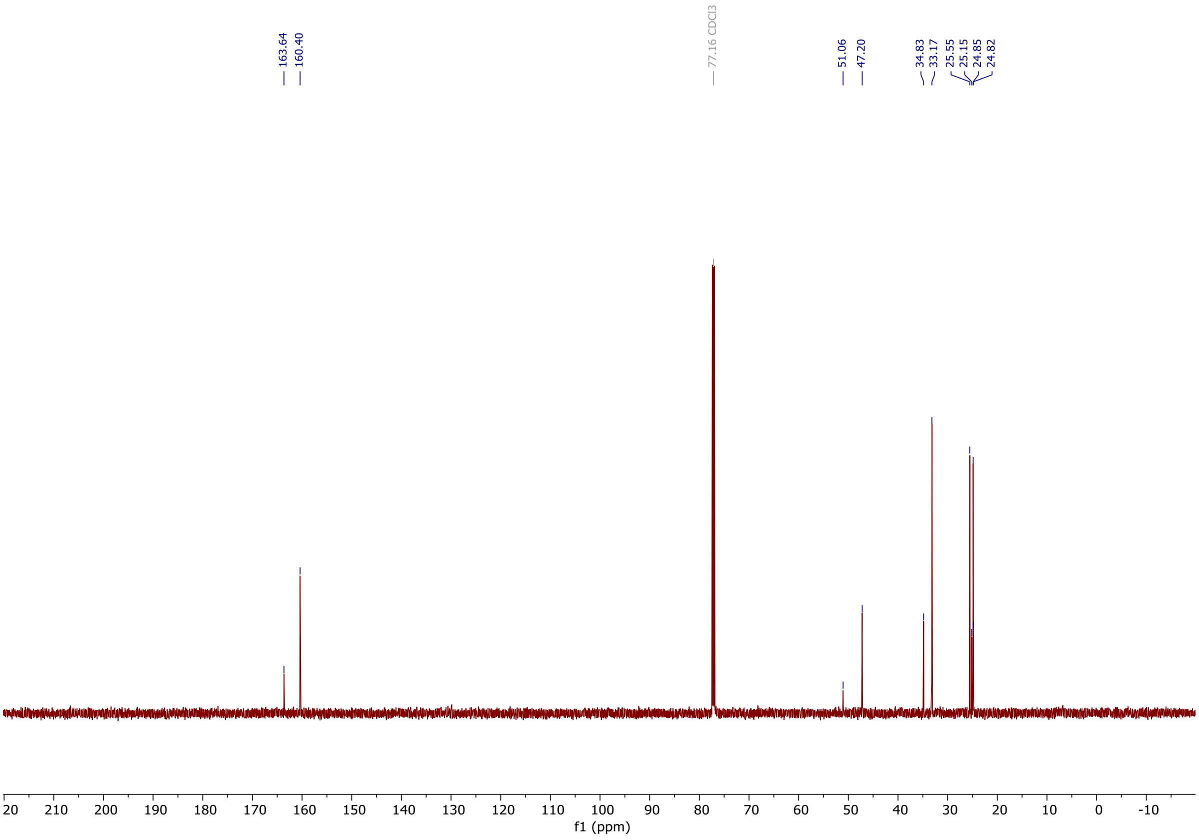

Pyrrolidine-1-carbaldehyde (**3a32**)

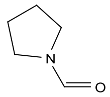

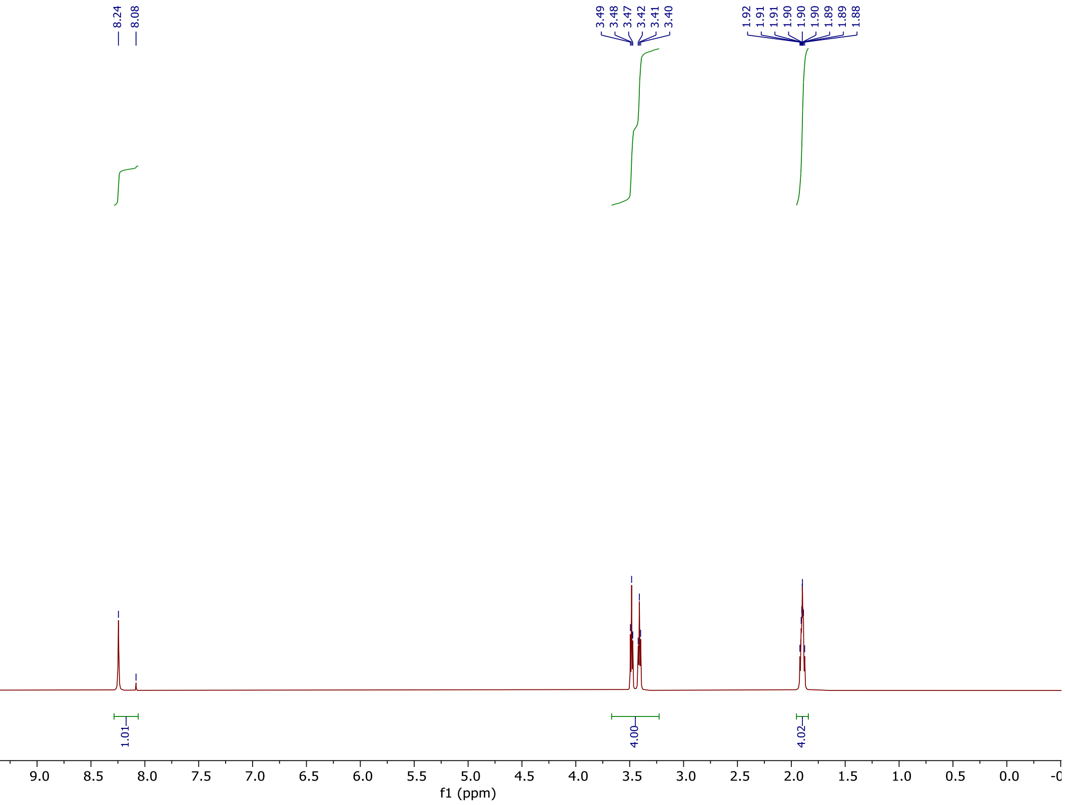

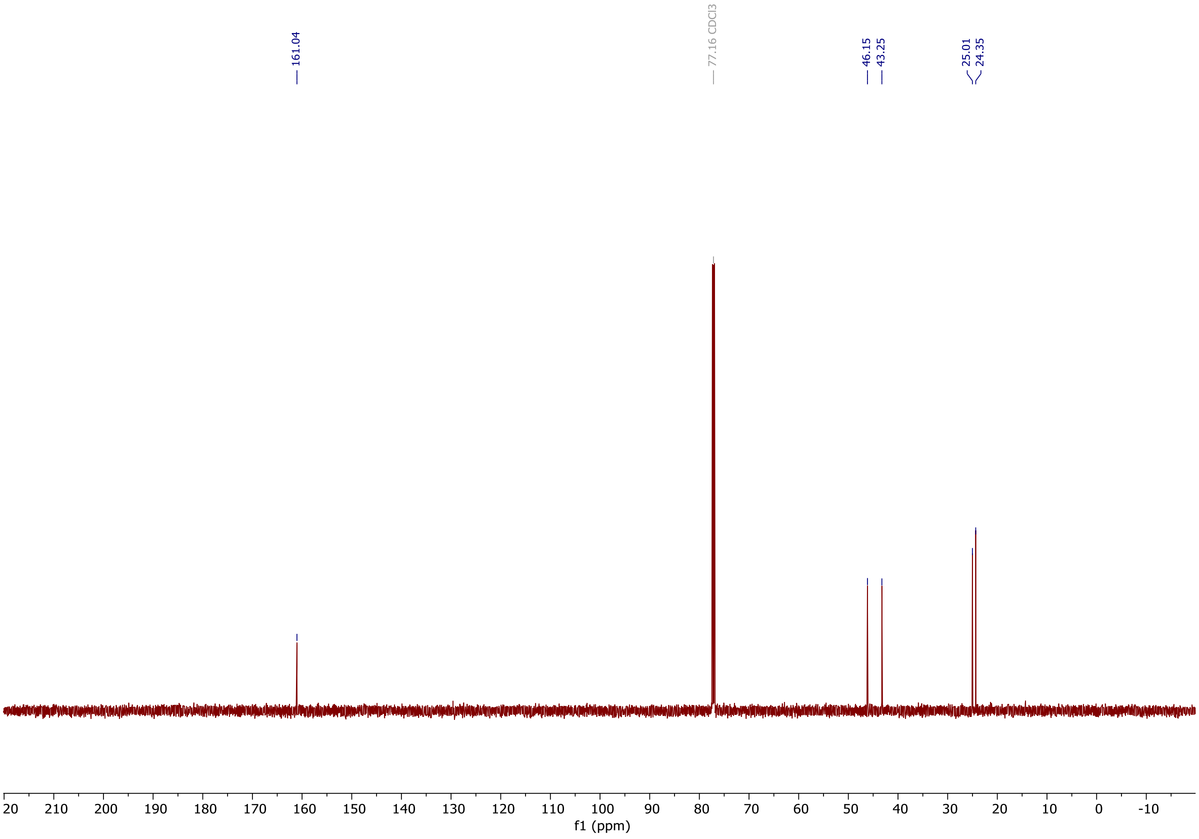

Piperidine-1-carbaldehyde (**3a**33)

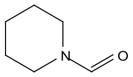

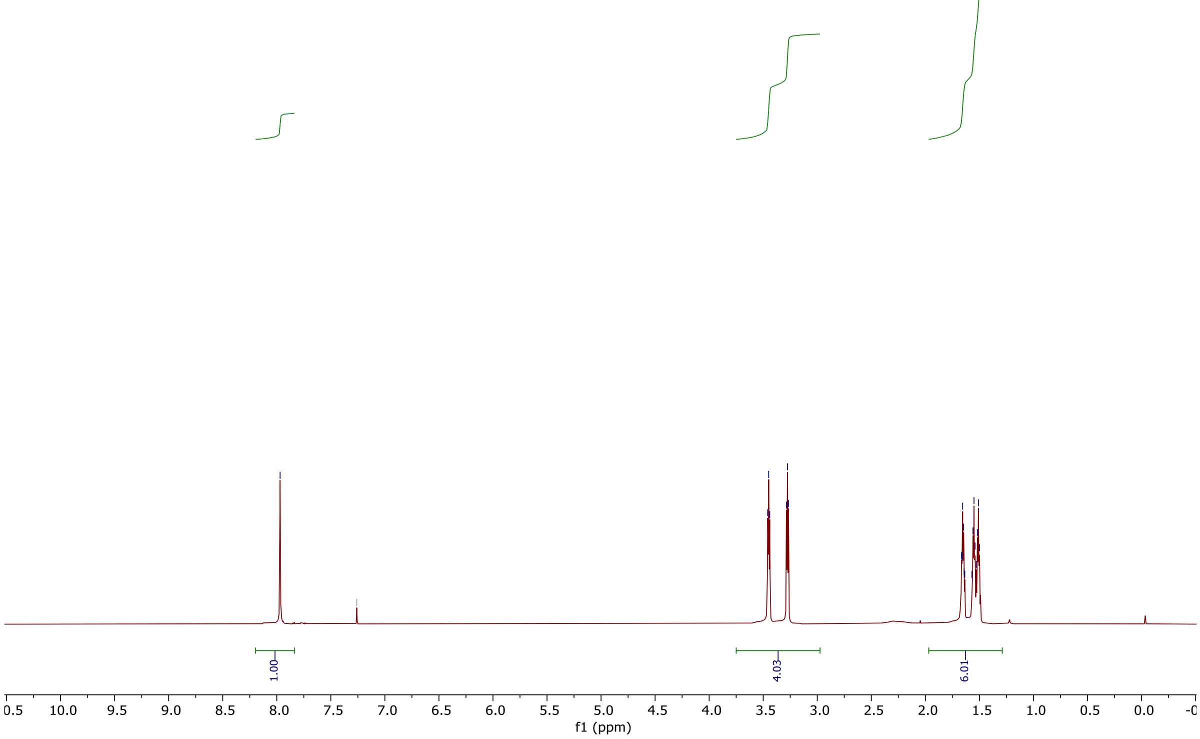

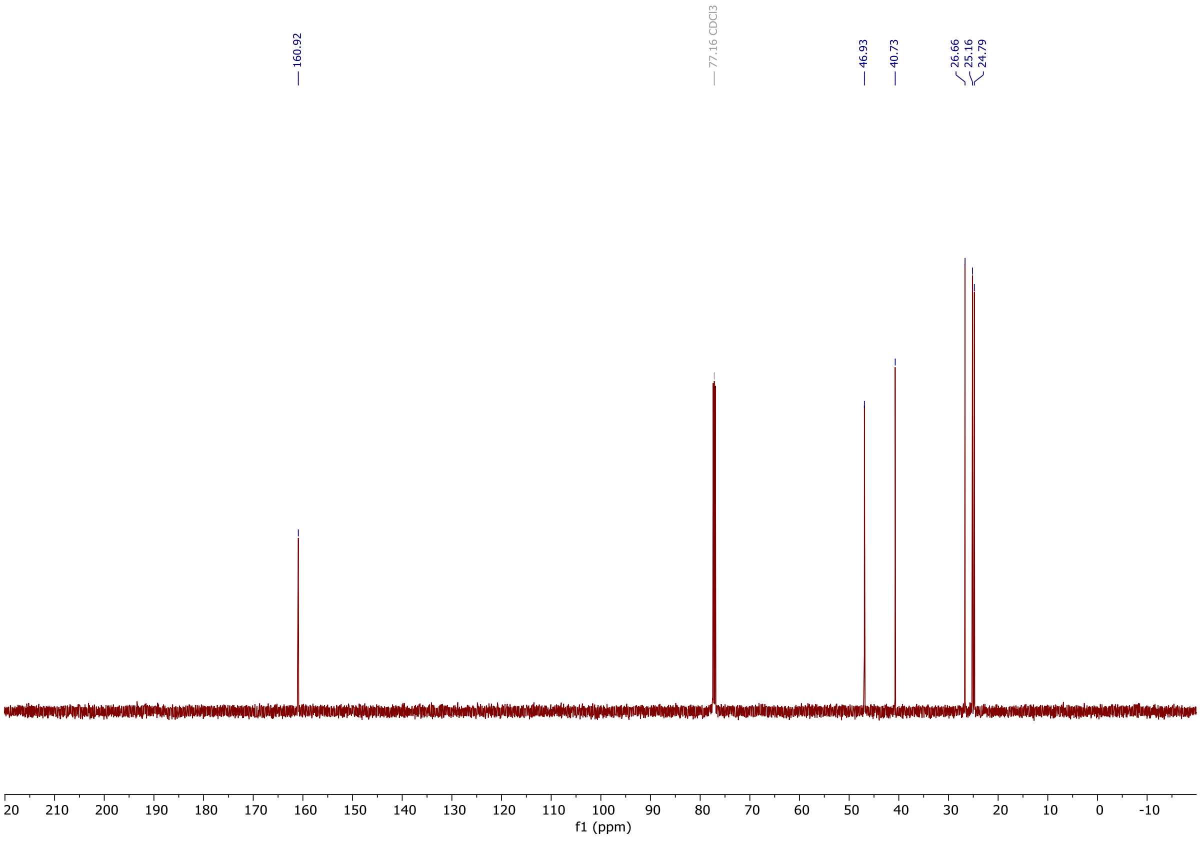

Morpholine-4-carbaldehyde (**3a**34)

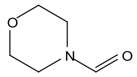

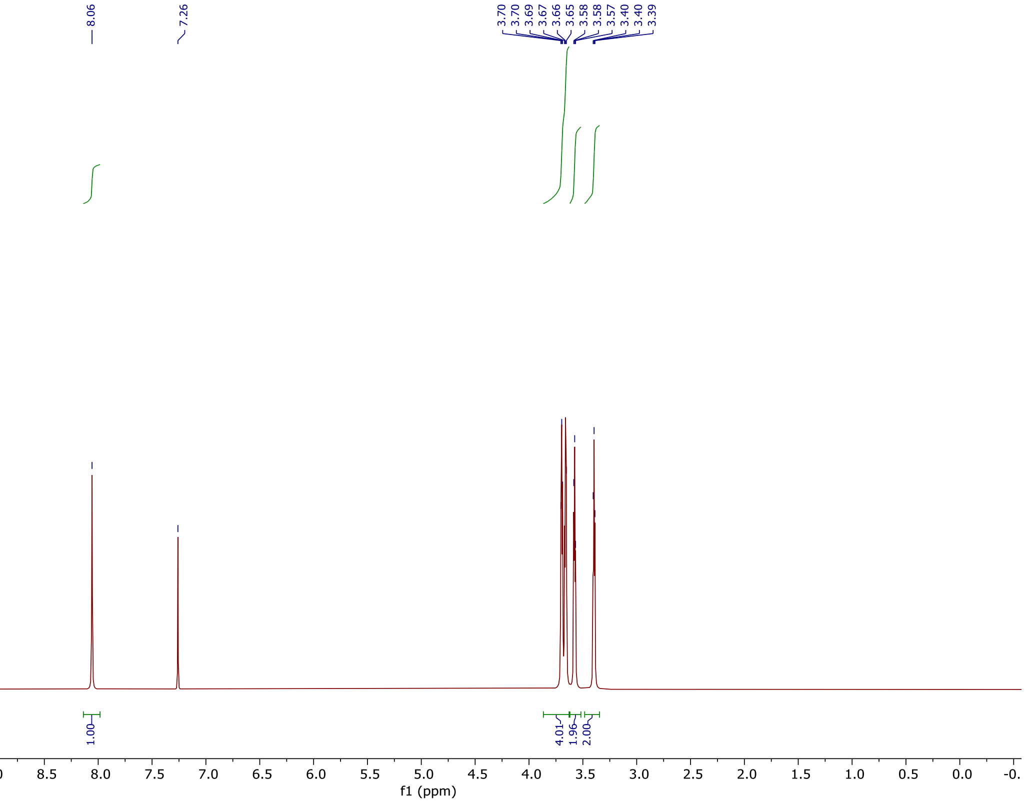

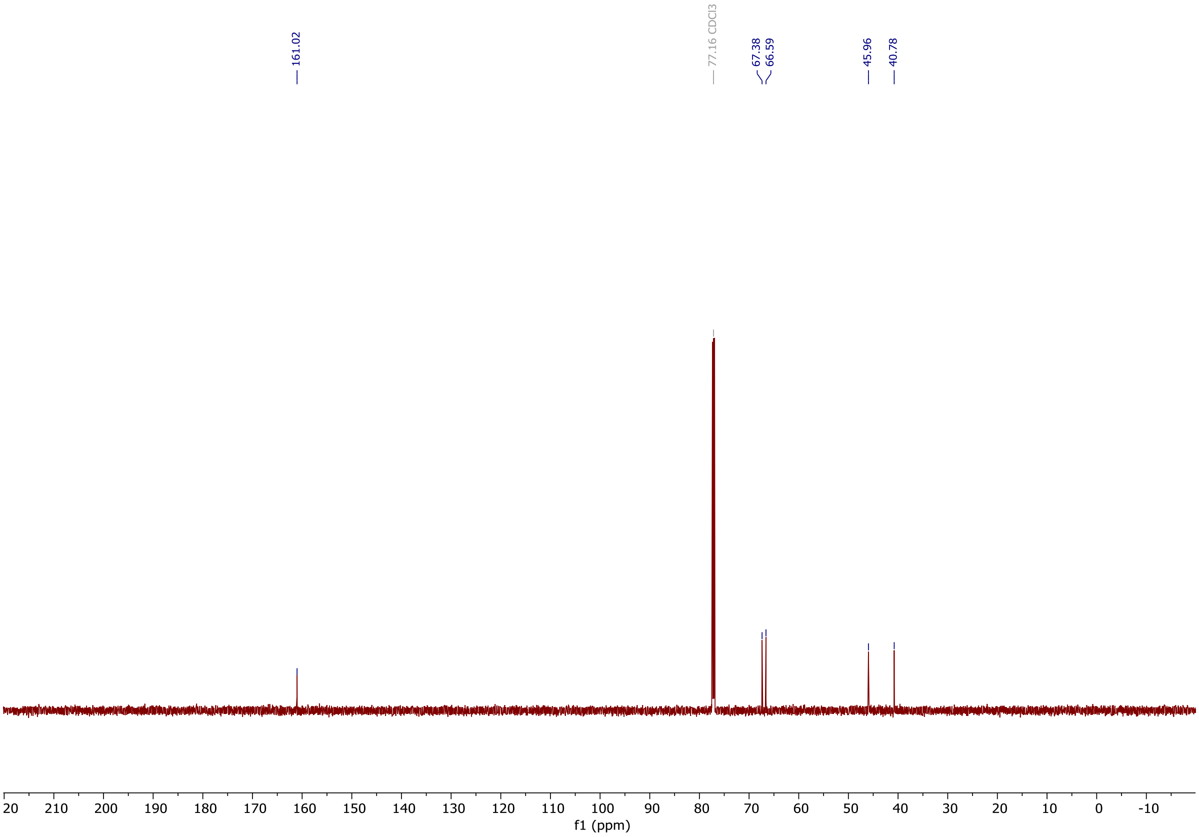

N-phenylacetamide (**4a**$_1$)

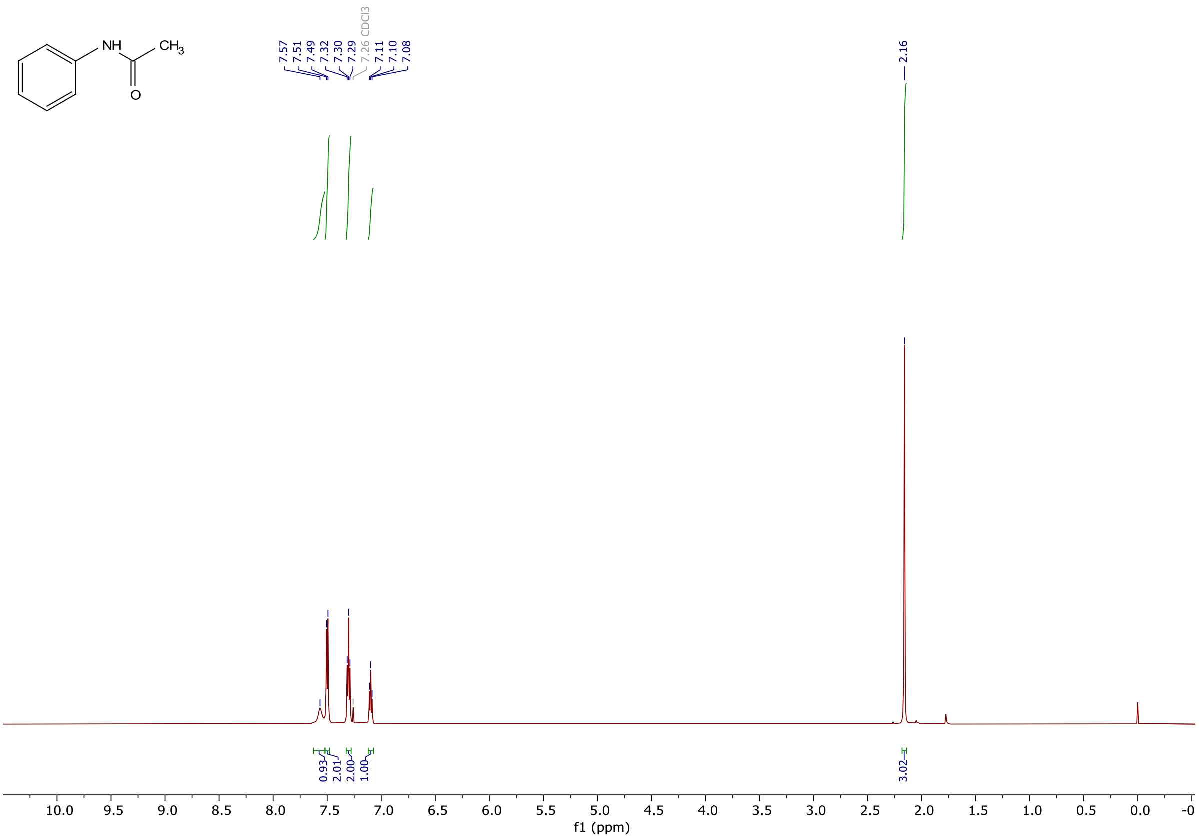

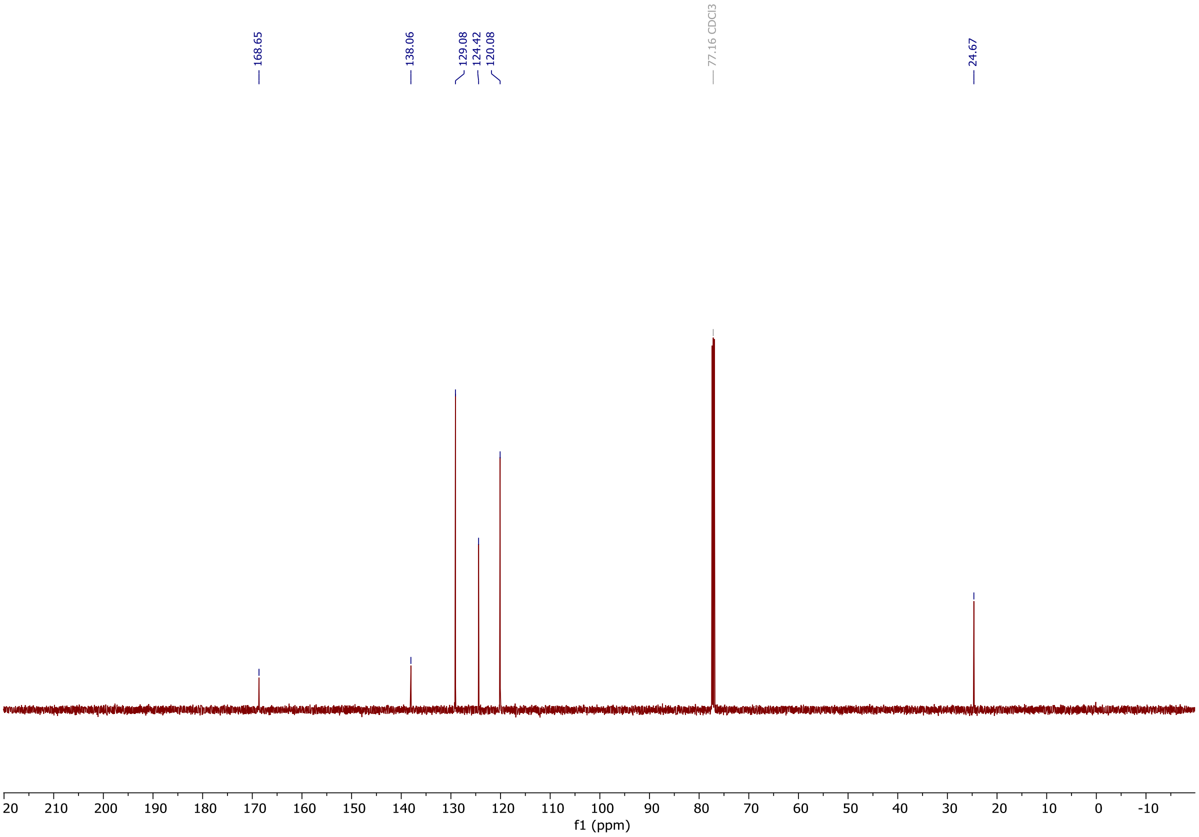

N-(4-fluorophenyl) acetamide (**4a3**)

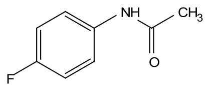

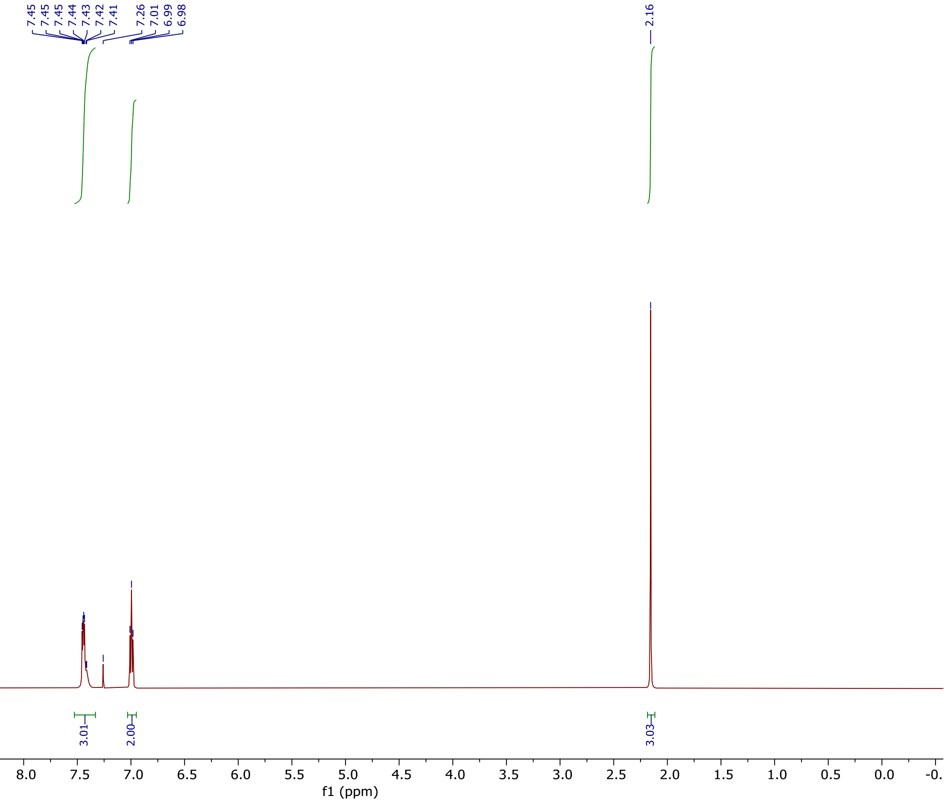

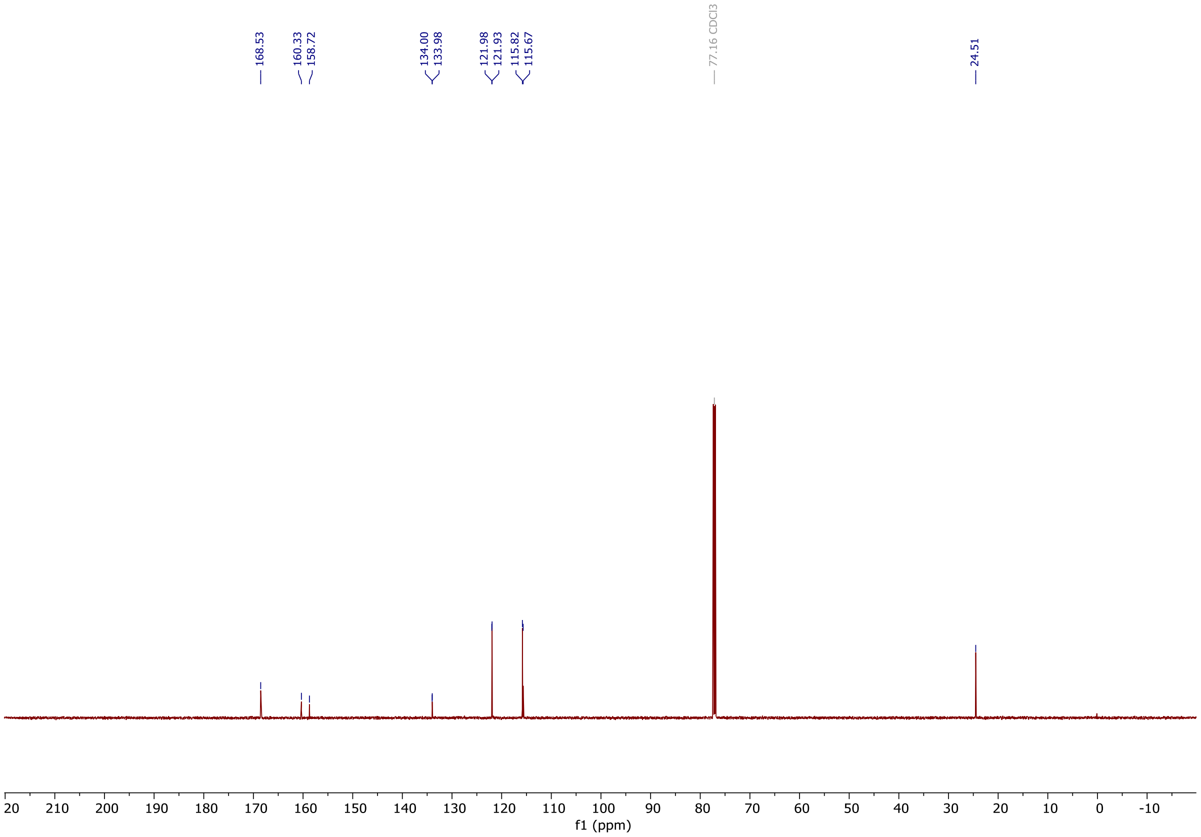

N-(4-bromophenyl) acetamide (**4a5**)

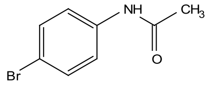

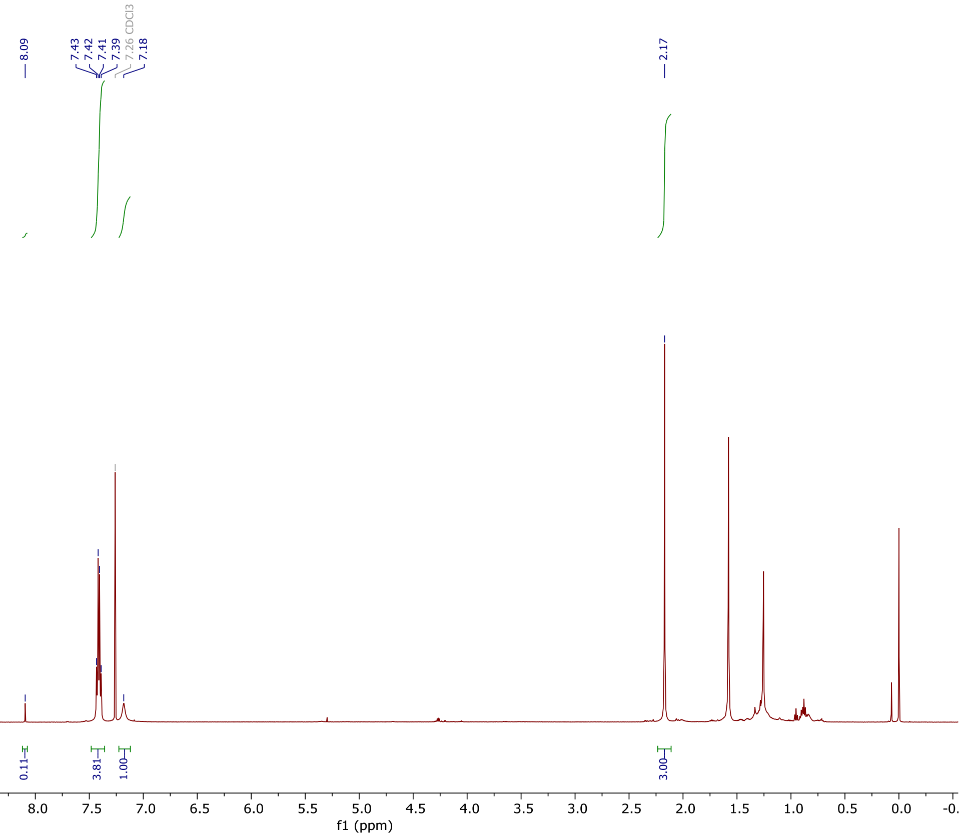

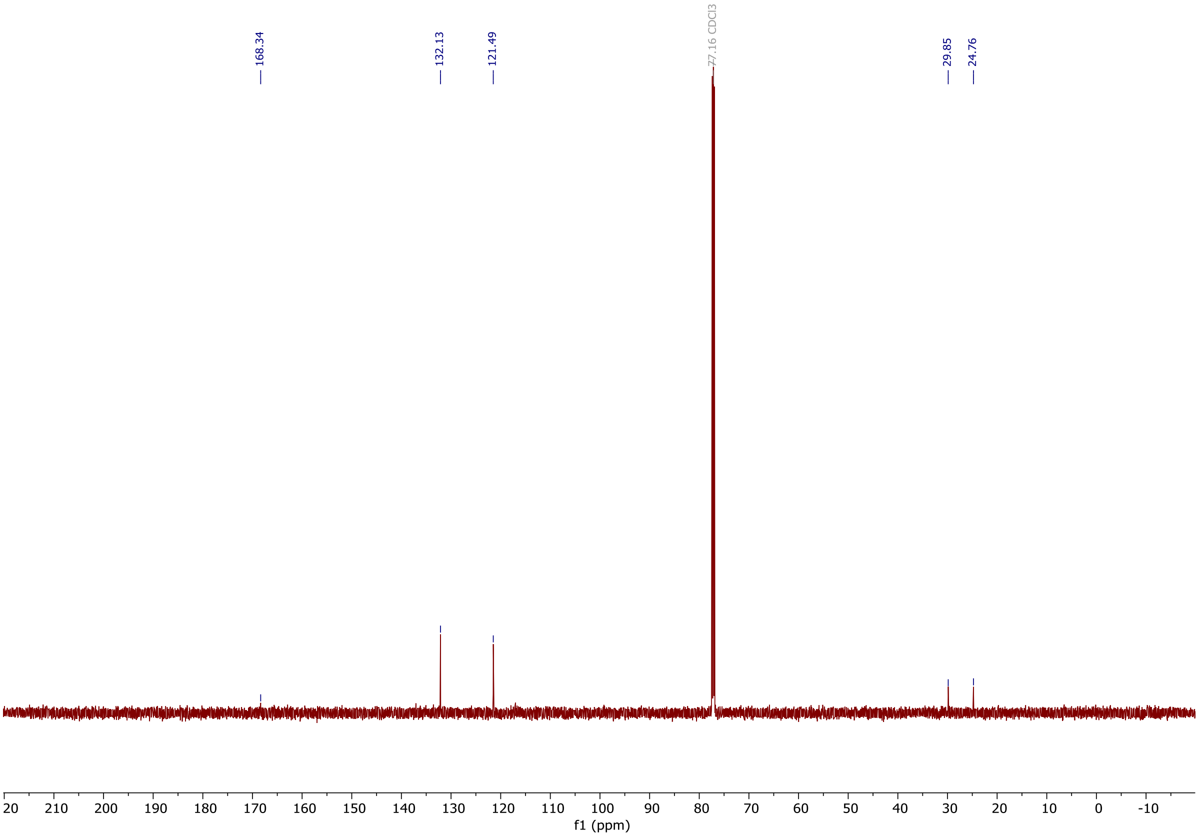

N-(2-iodophenyl) acetamide (**4a**$_6$)

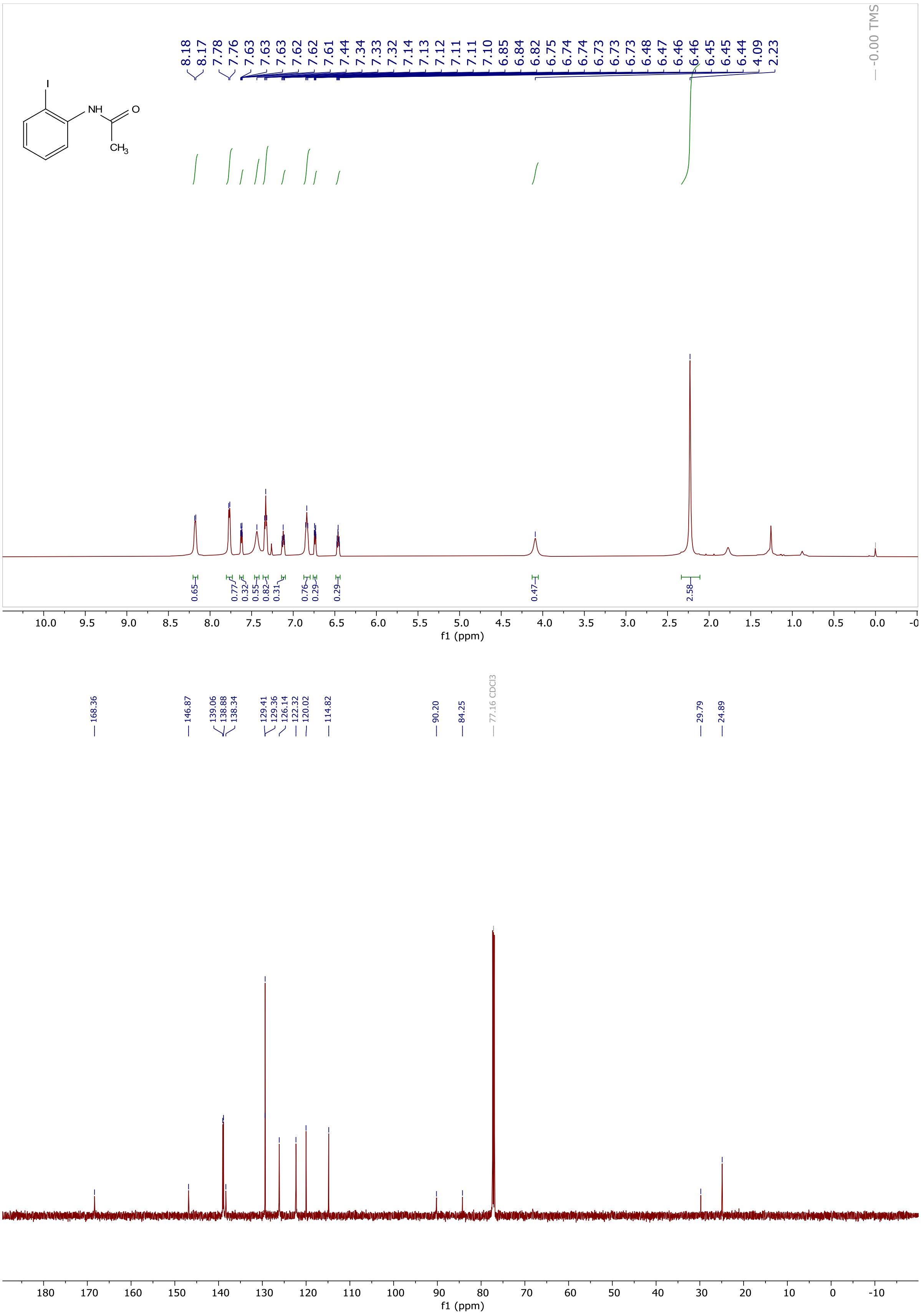

N-(4-hydroxyphenyl) acetamide (**4a**11)

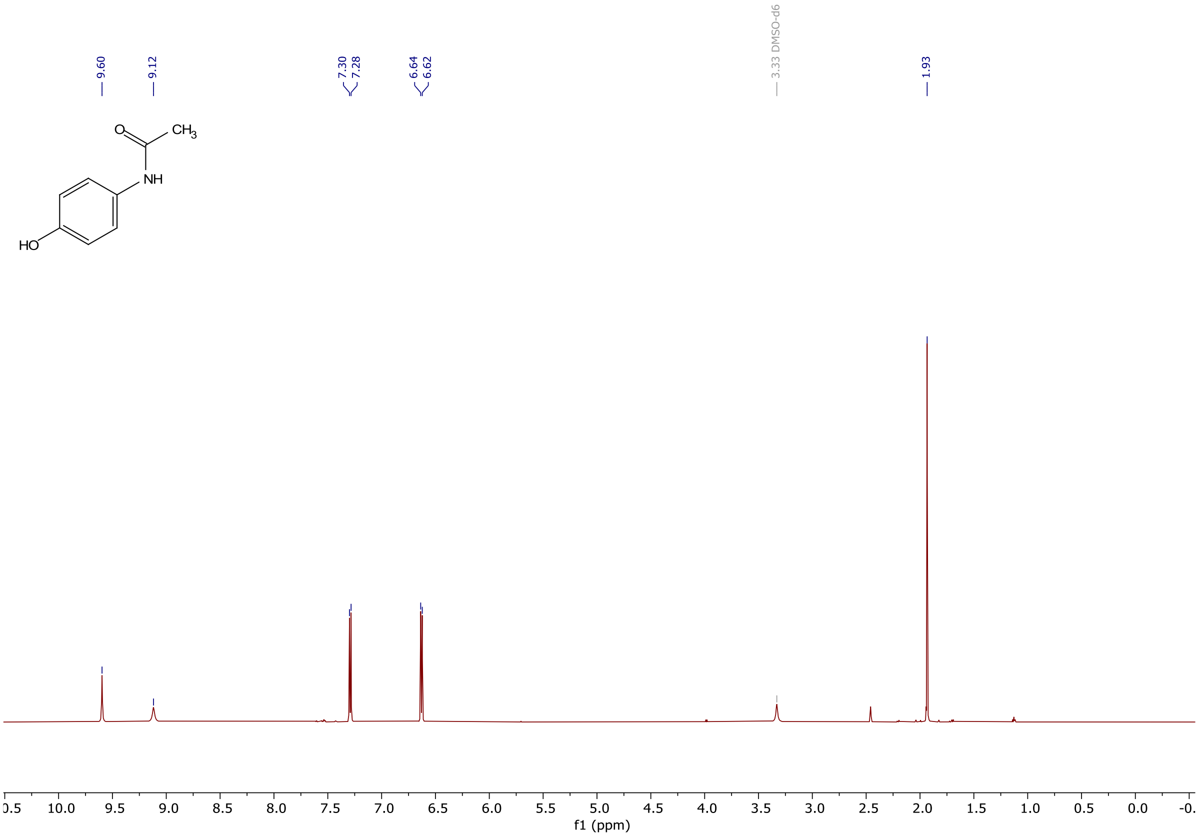

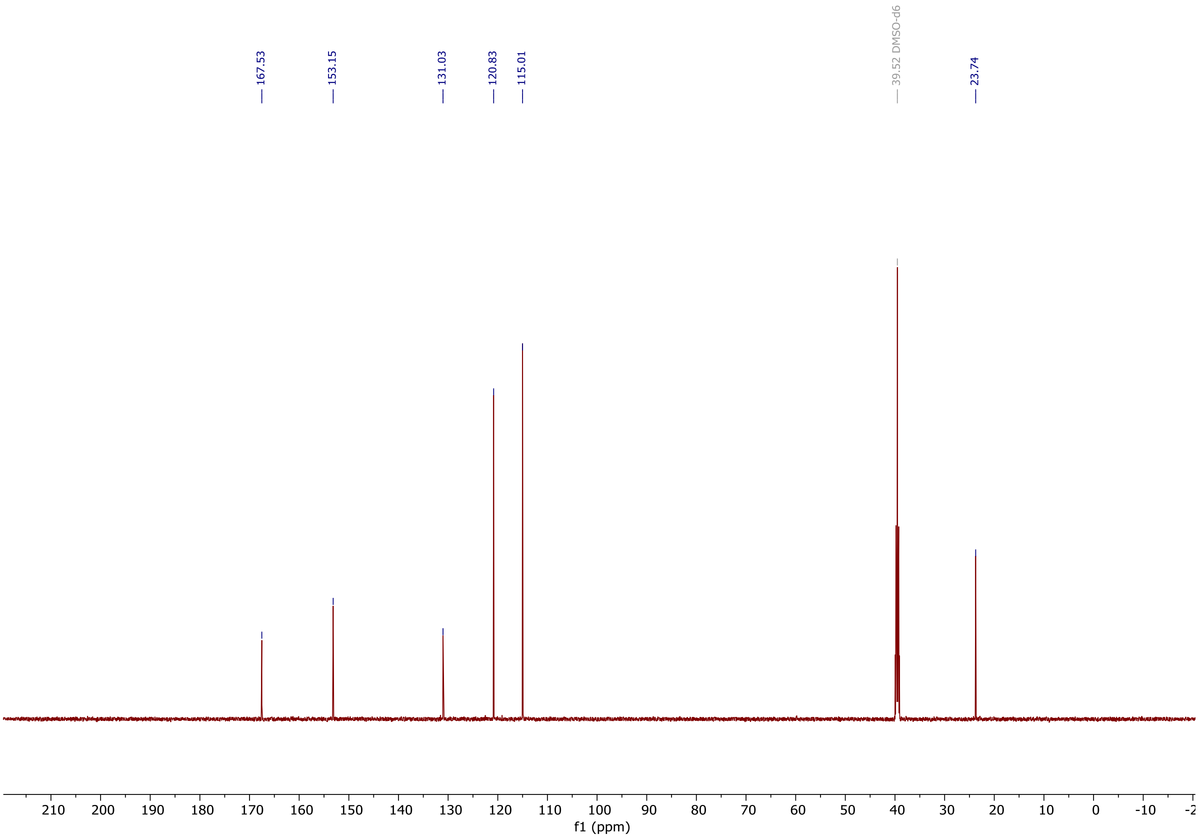

N-benzylacetamide (**4a18**)

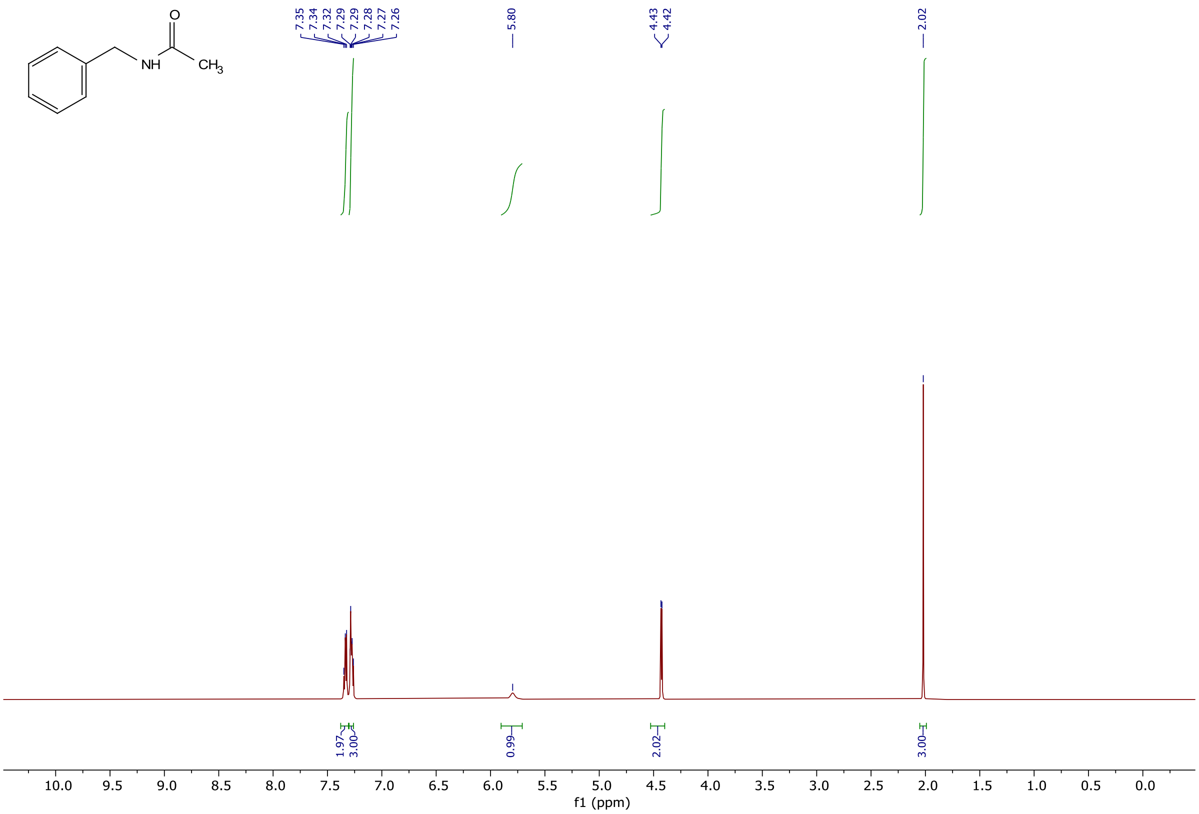

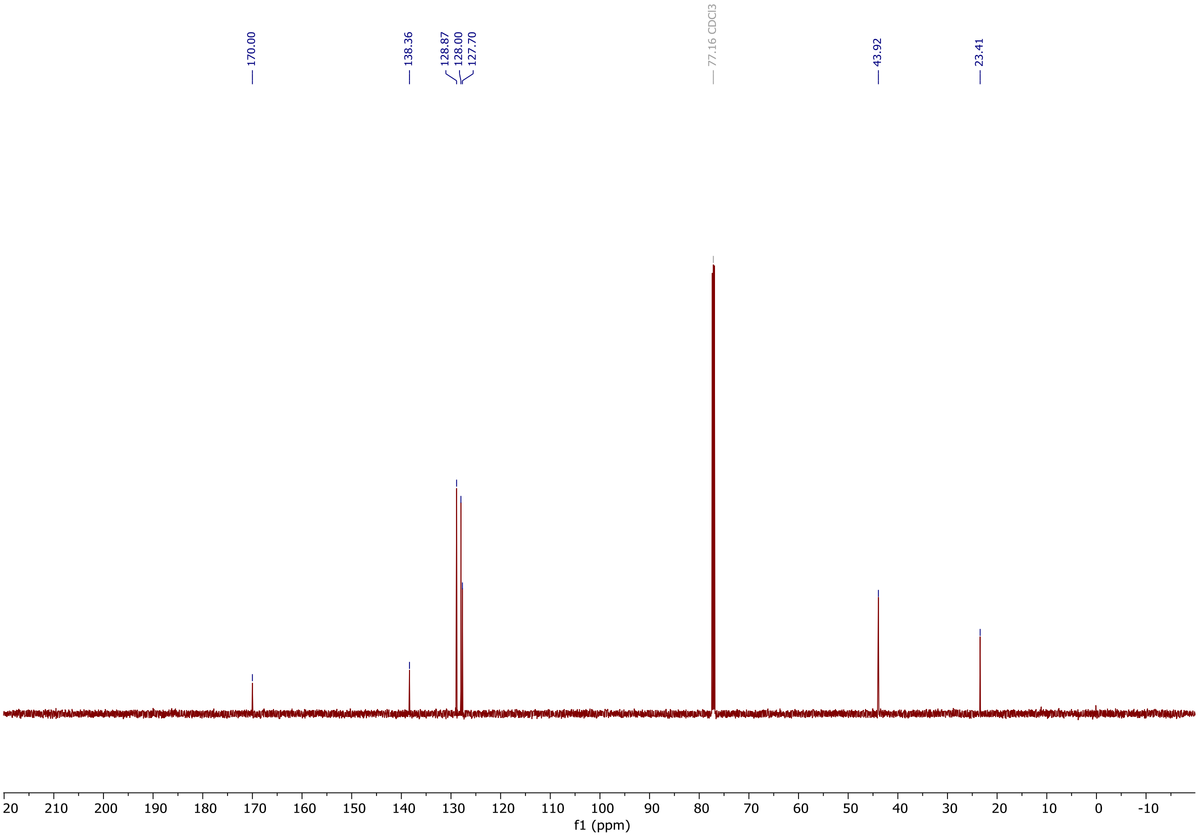

N-(furan-2-ylmethyl) acetamide (**4a21**)

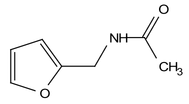

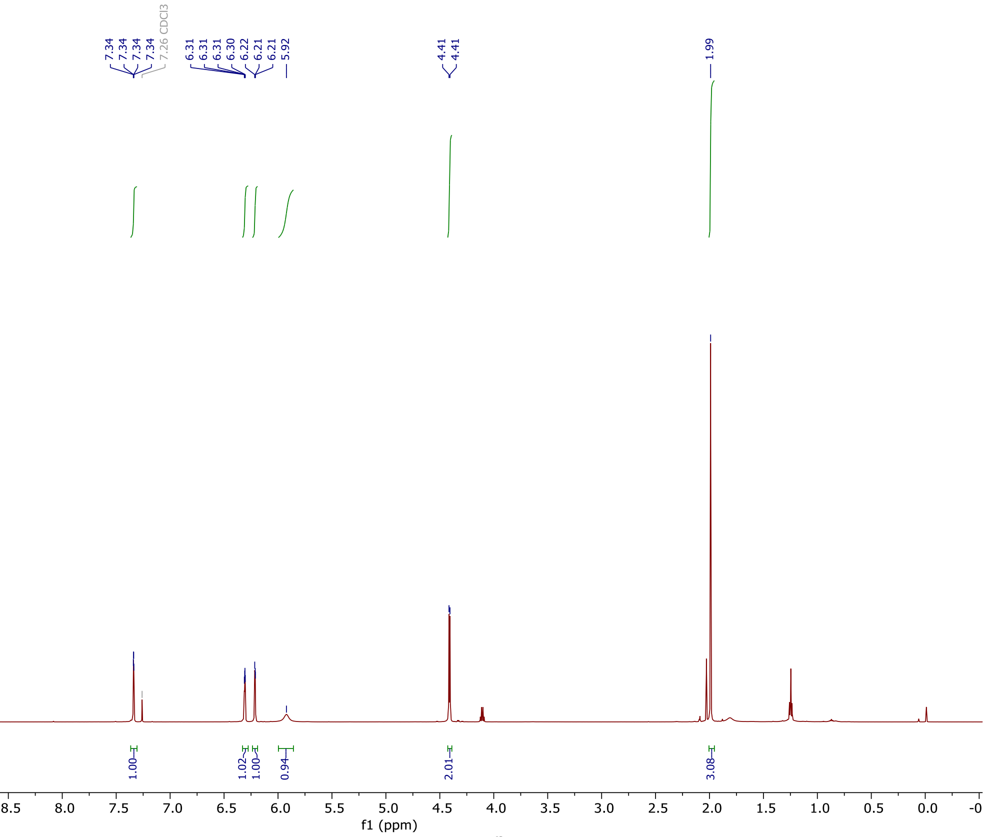

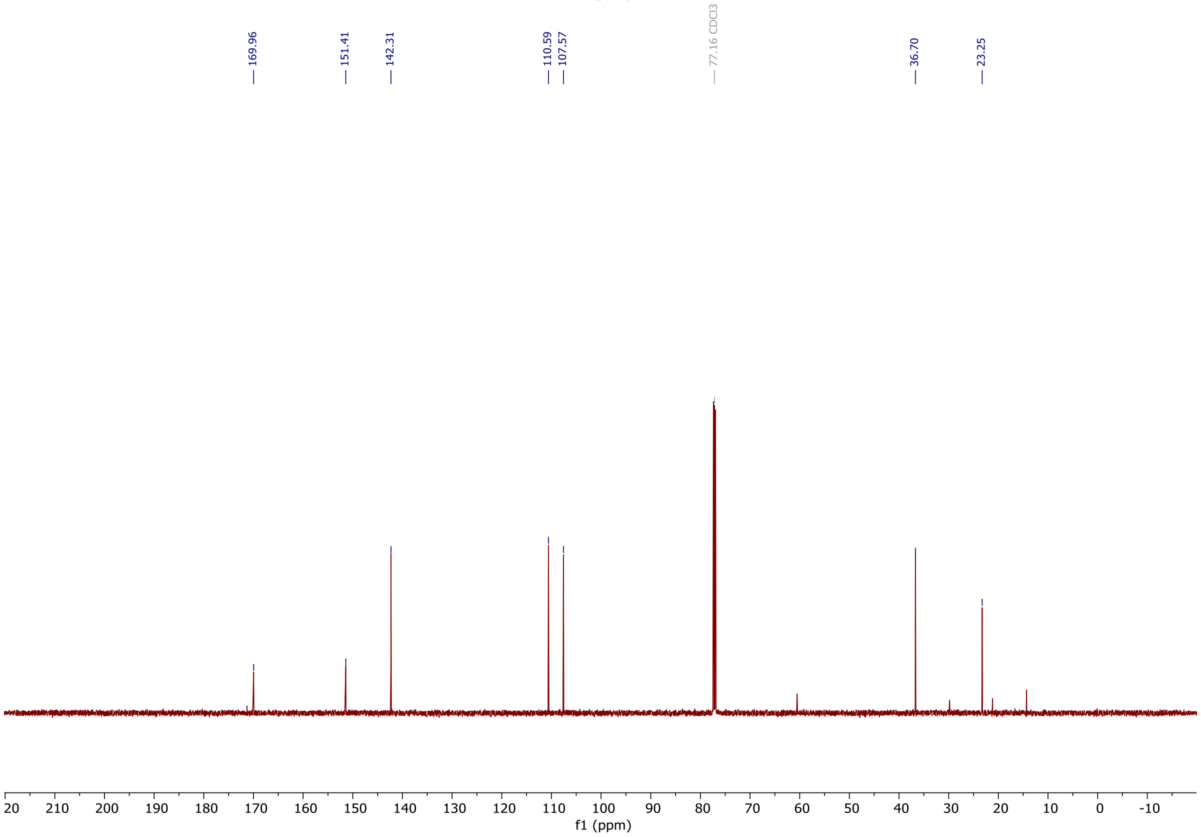

N-benzyl-*N*-methylacetamide (**4a**23)

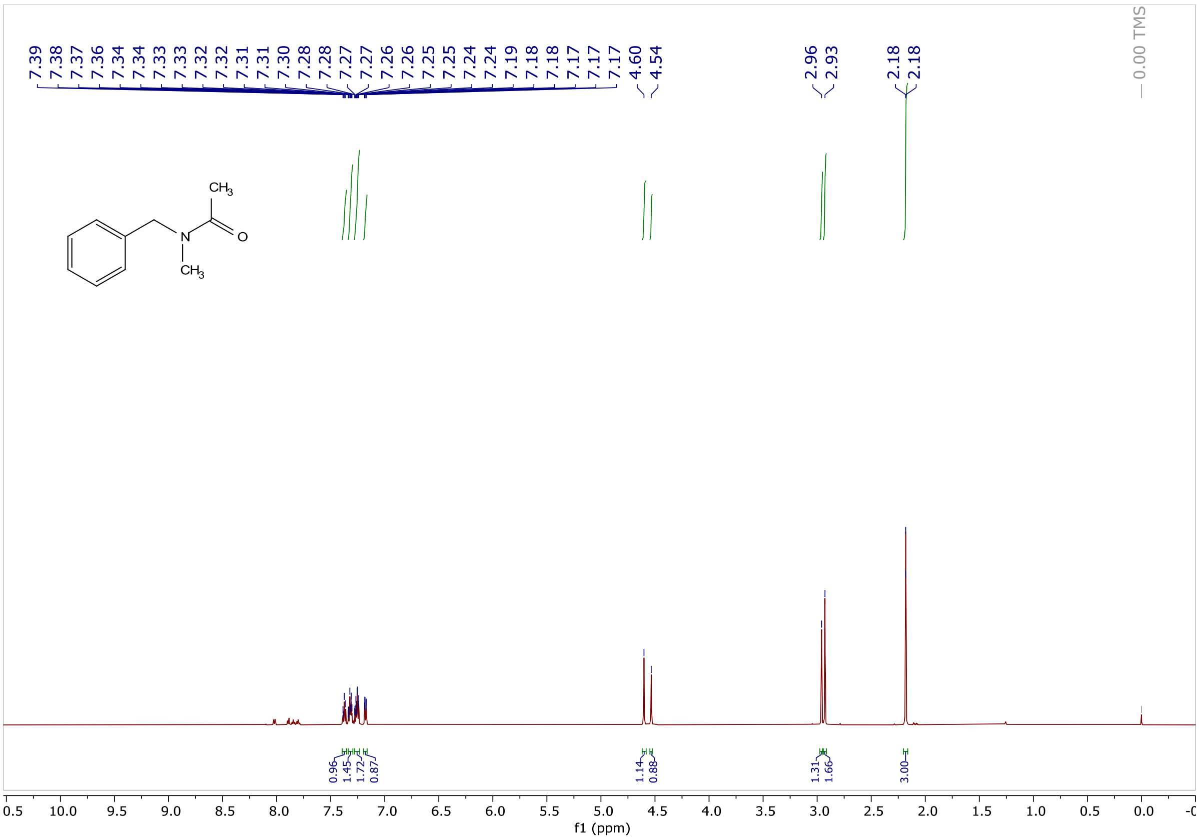

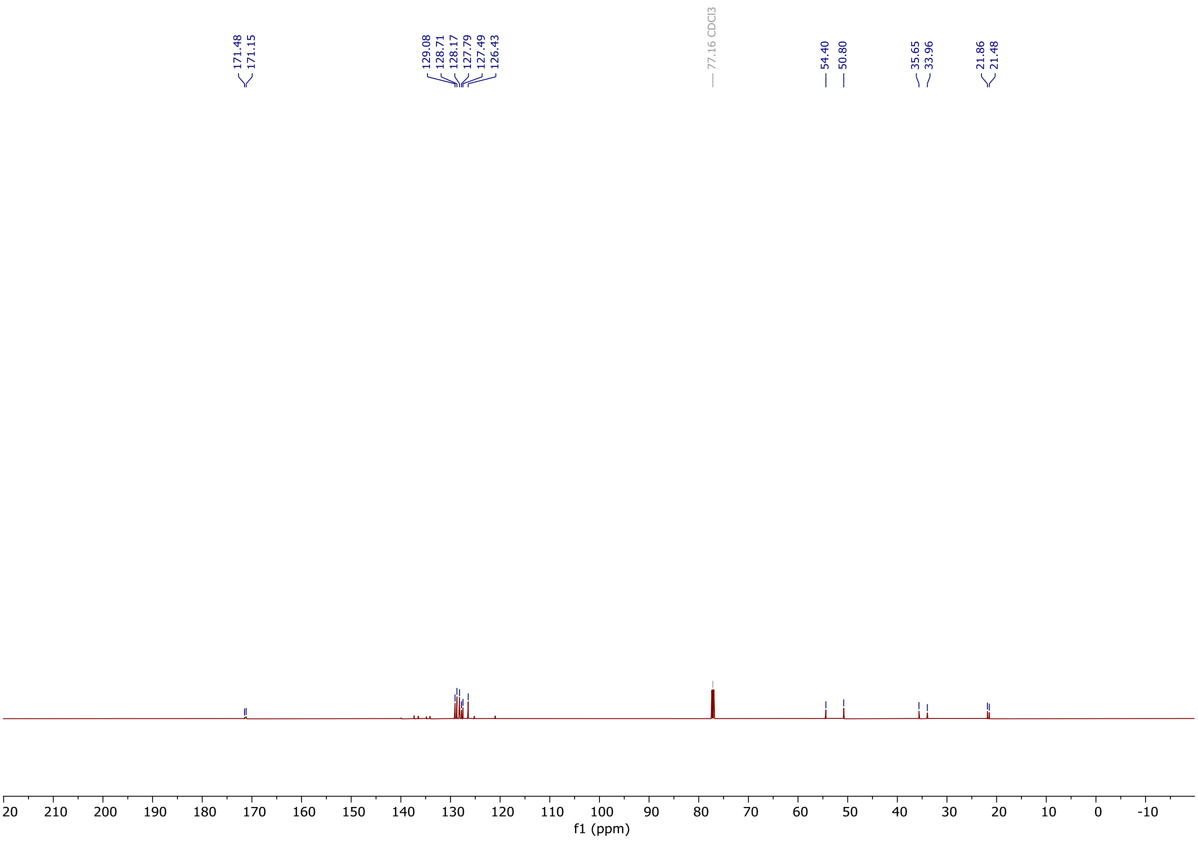

N-octylacetamide (**4a28**)

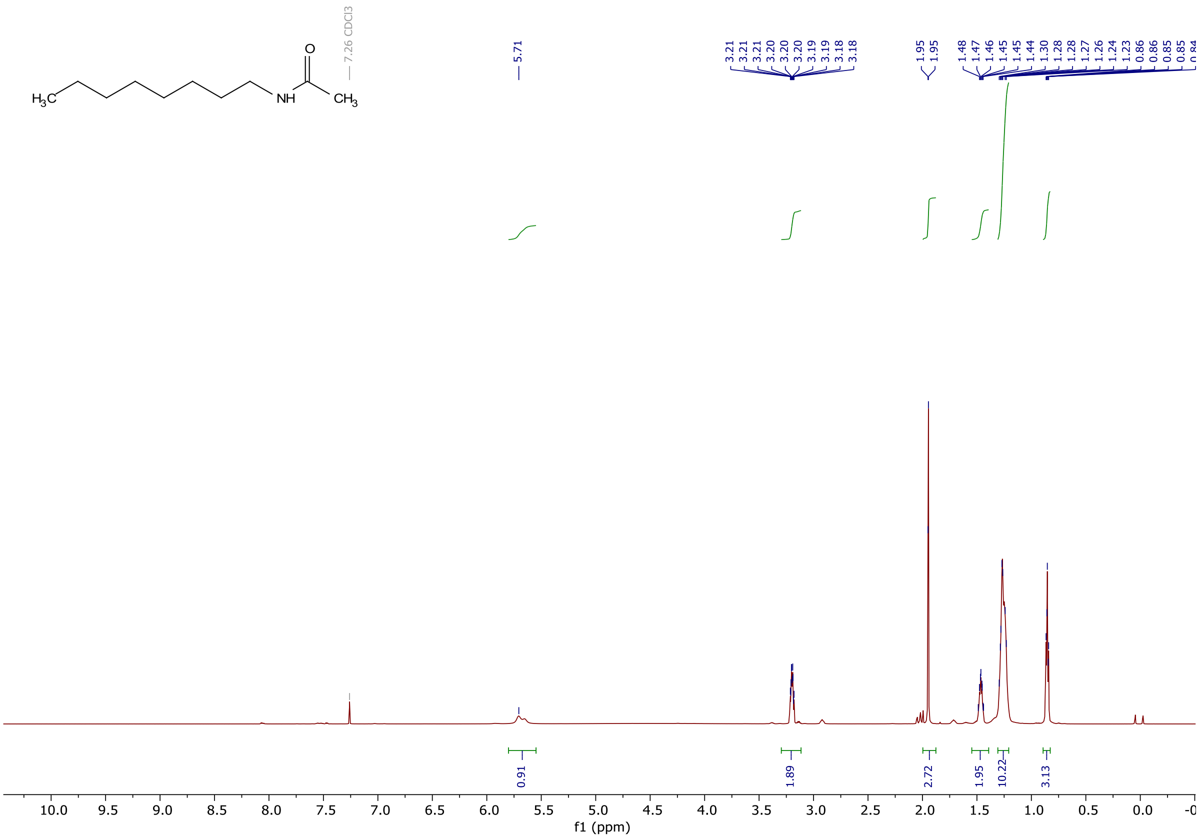

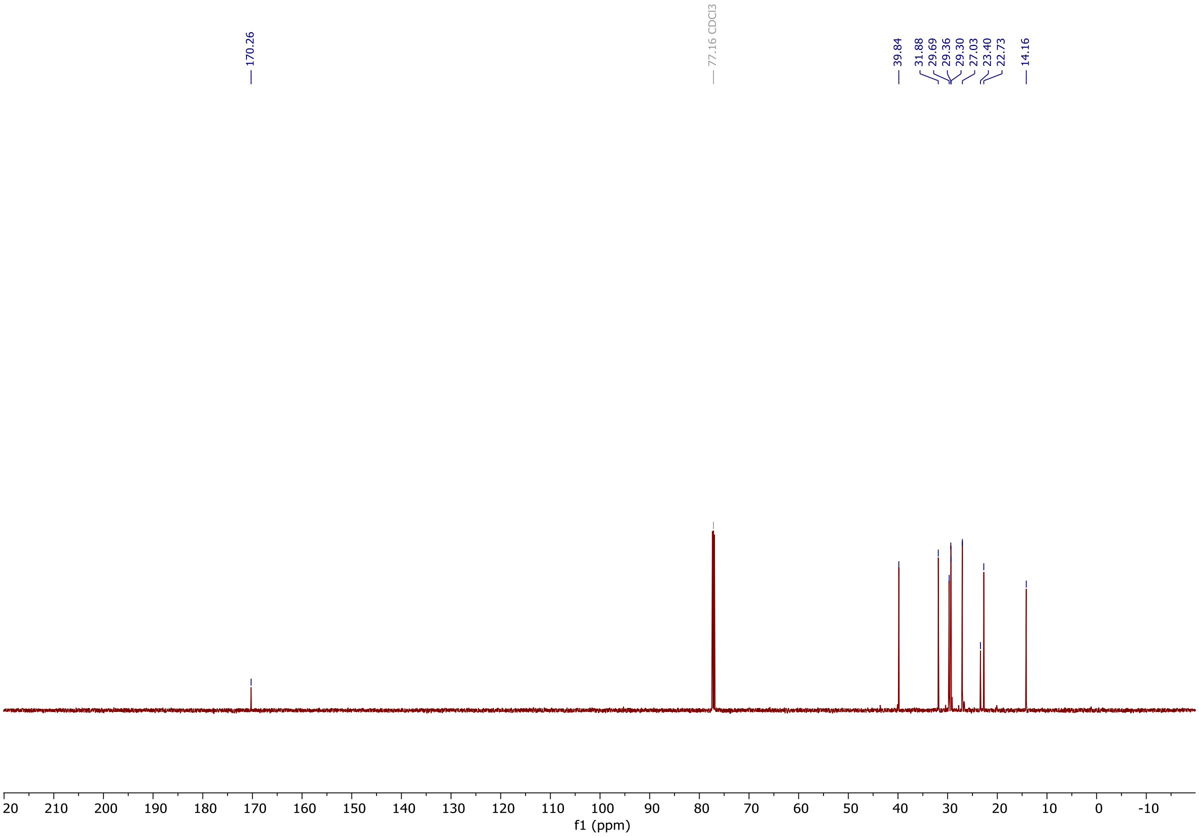

N-phenylpropionamide (**5a**1)

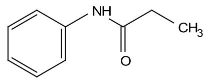

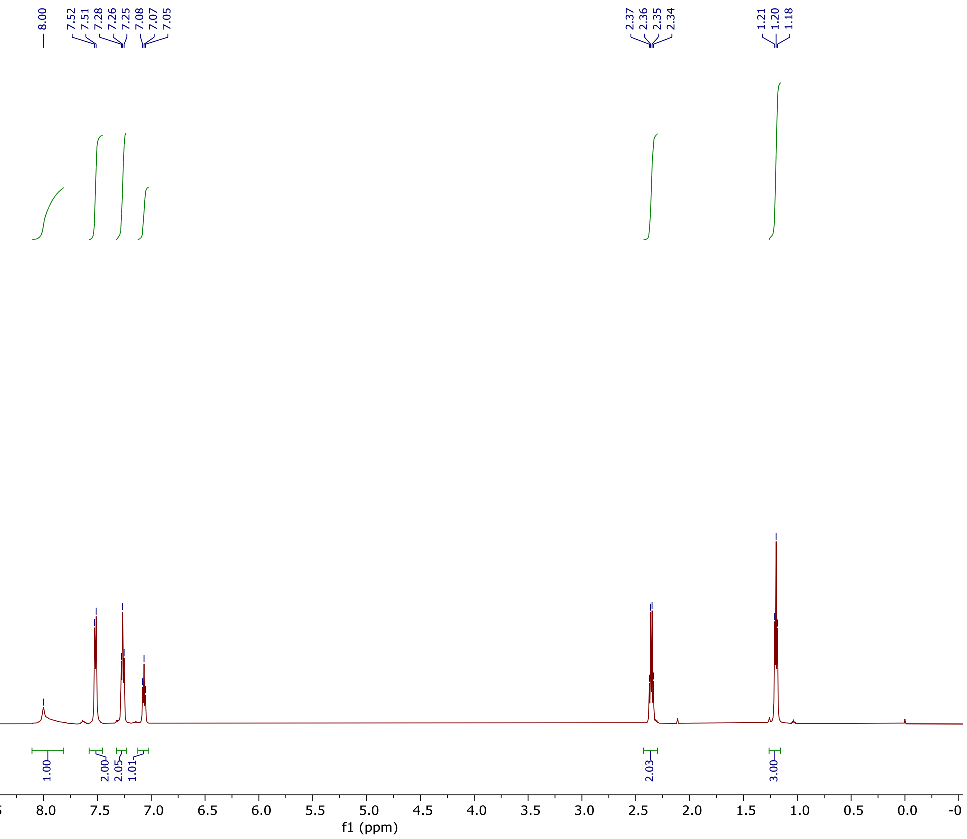

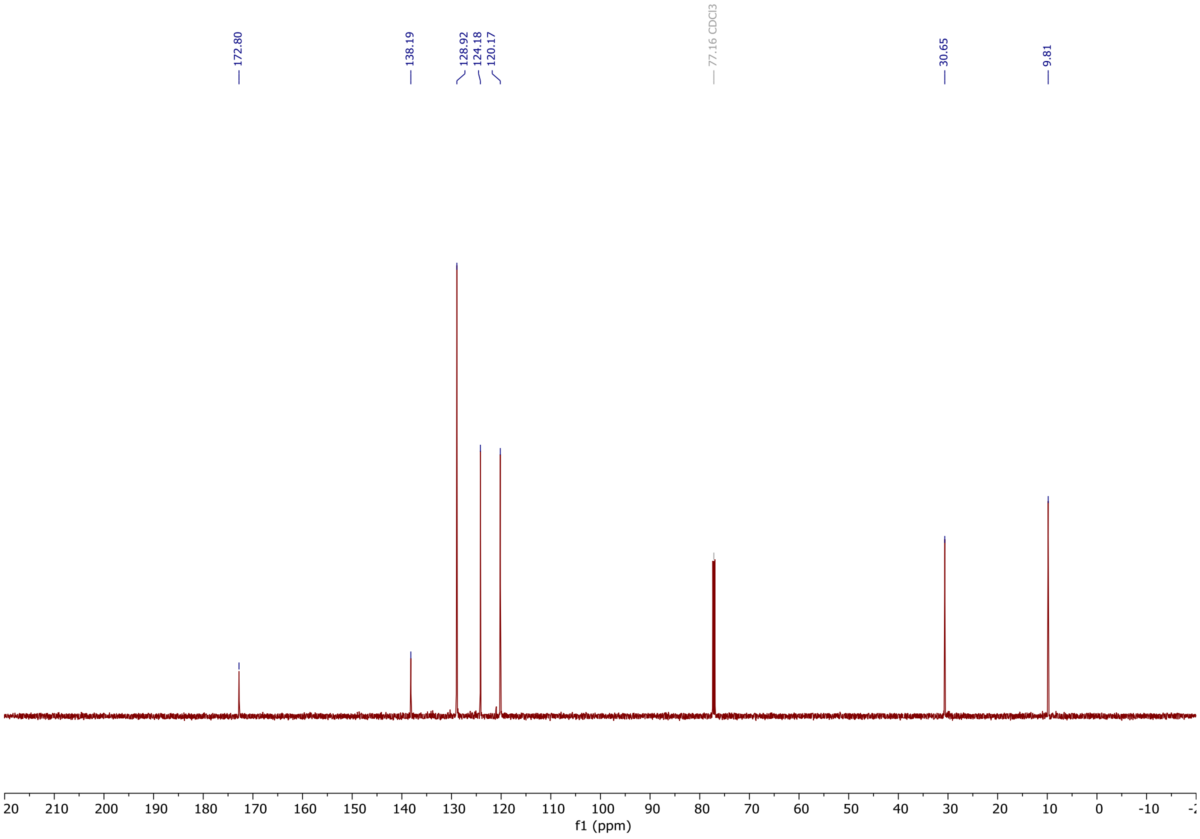

N-(2,4-dimethylphenyl) propionamide (**5a7**)

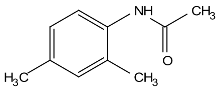

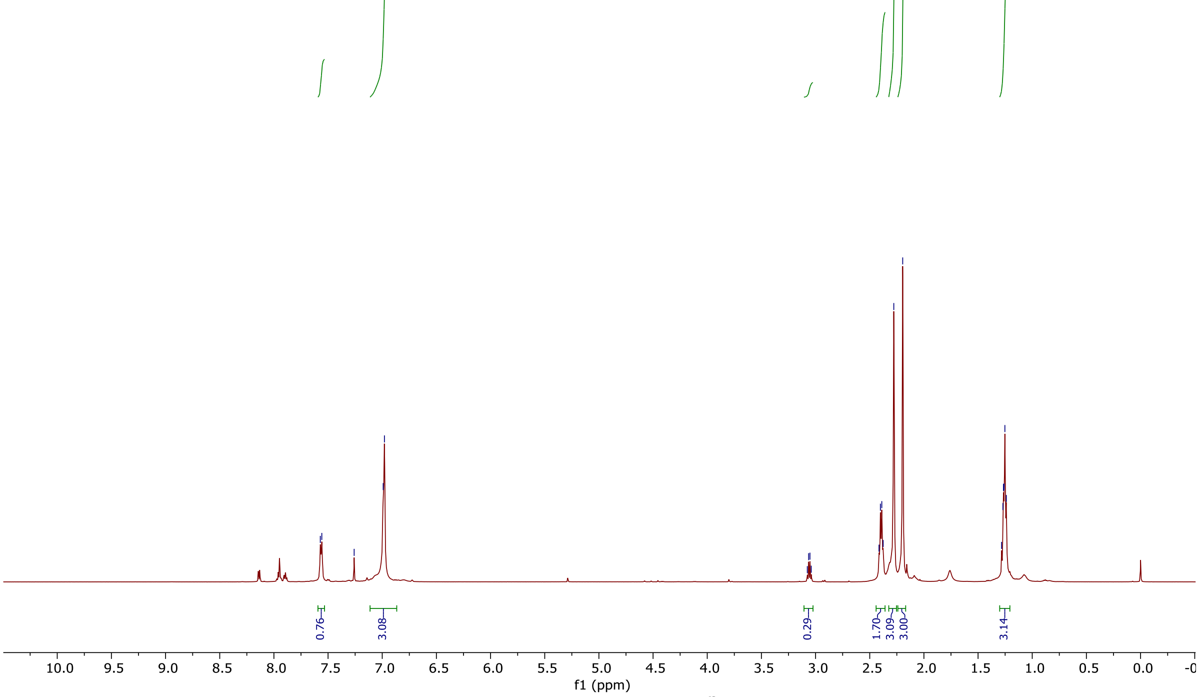

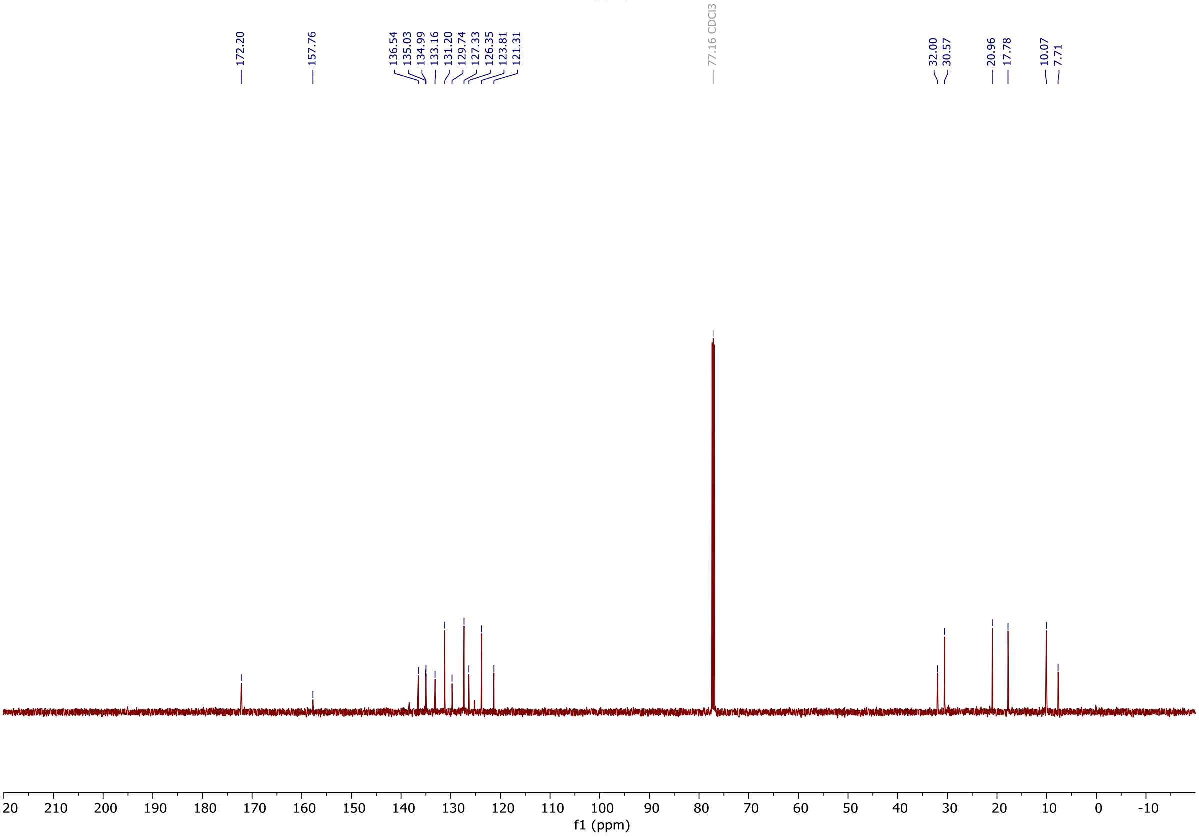

N-(4-methoxyphenyl) propionamide (**5a**12)

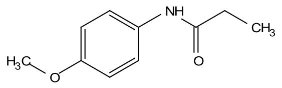

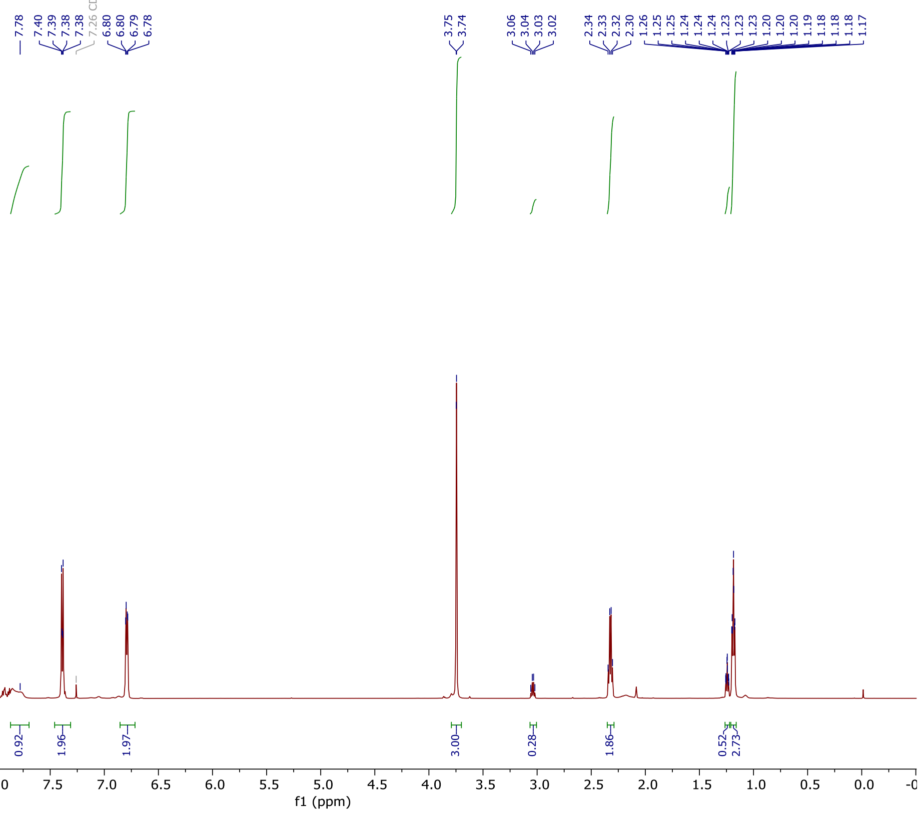

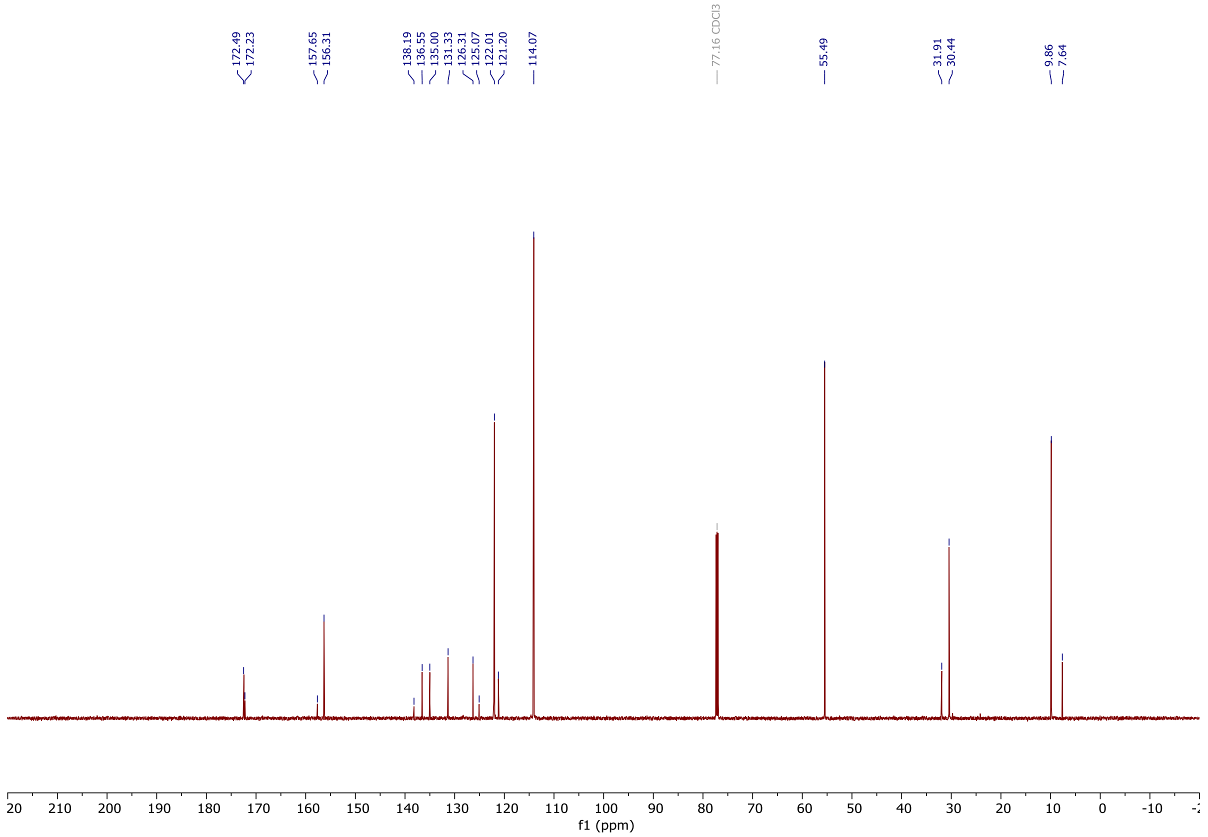

N-(naphthalen-1-yl) propionamide (**5a**15)

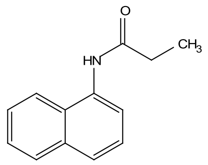

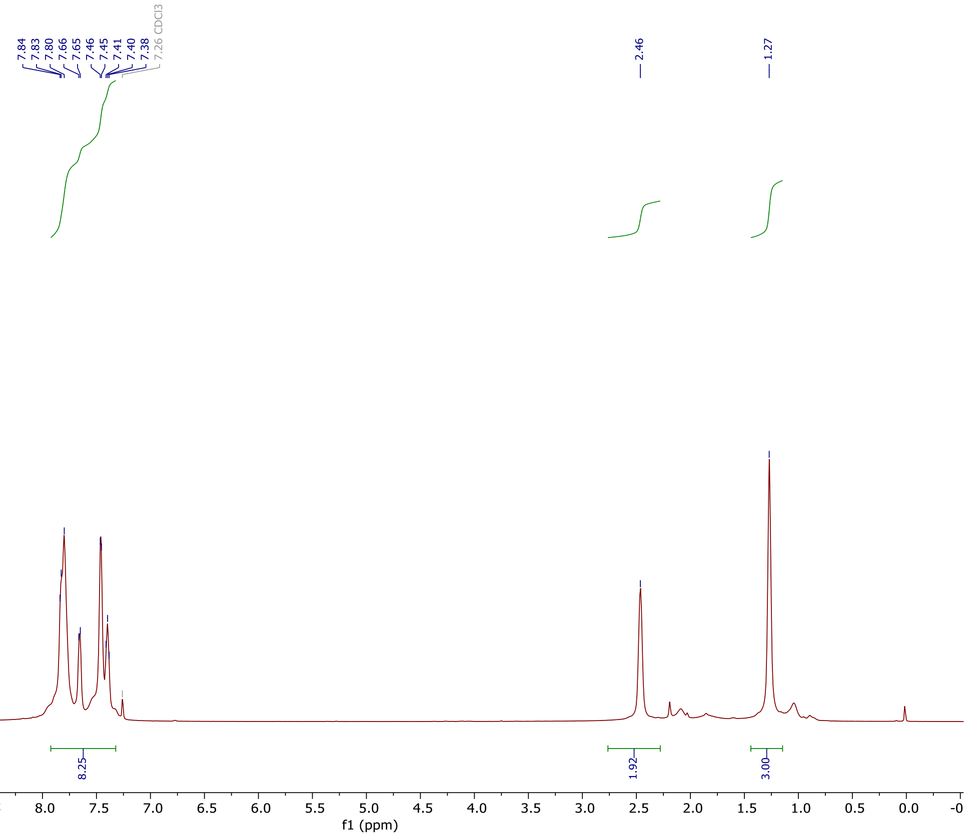

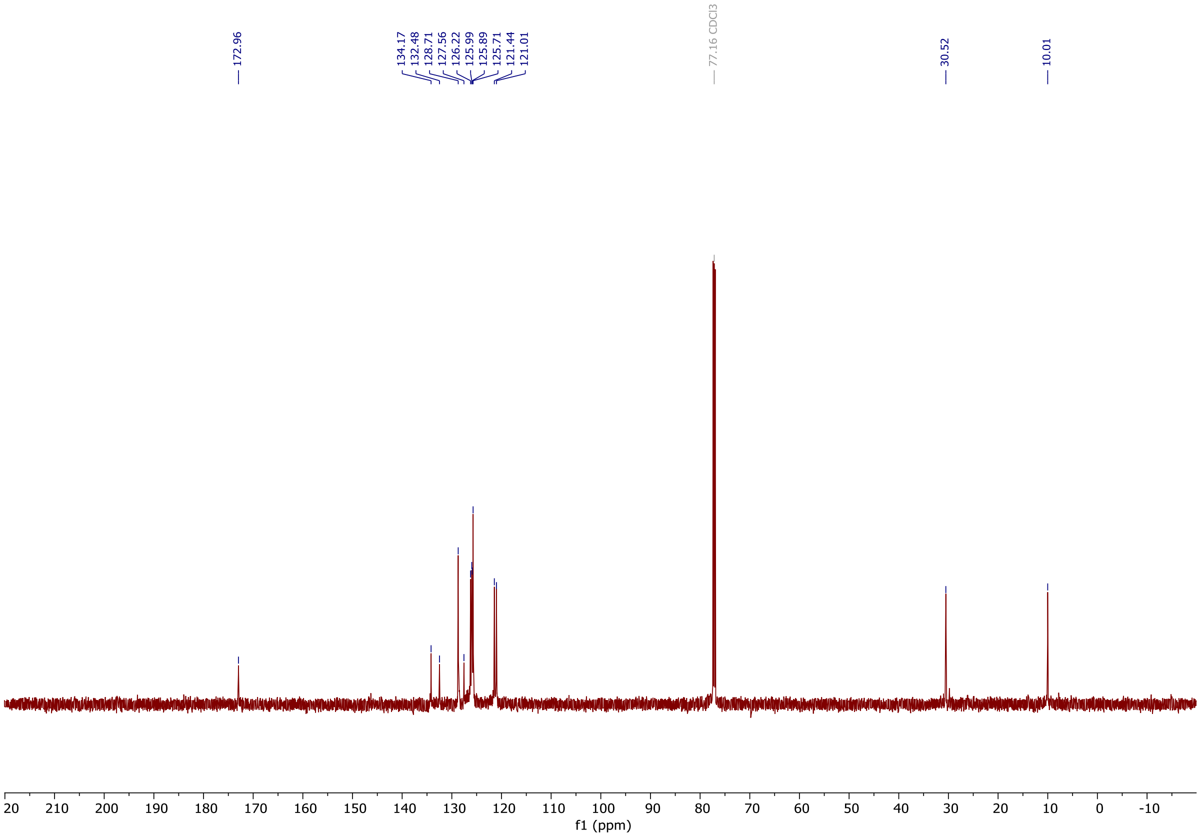

N-benzylpropionamide (**5a**18)

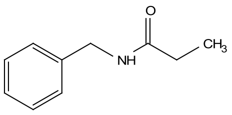

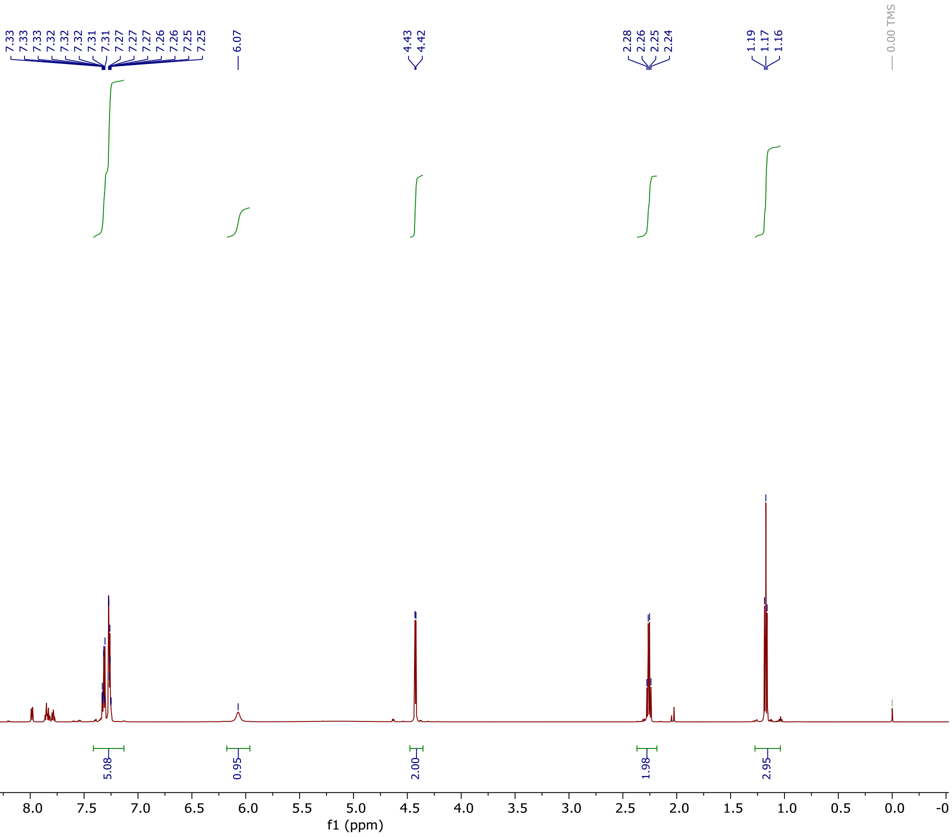

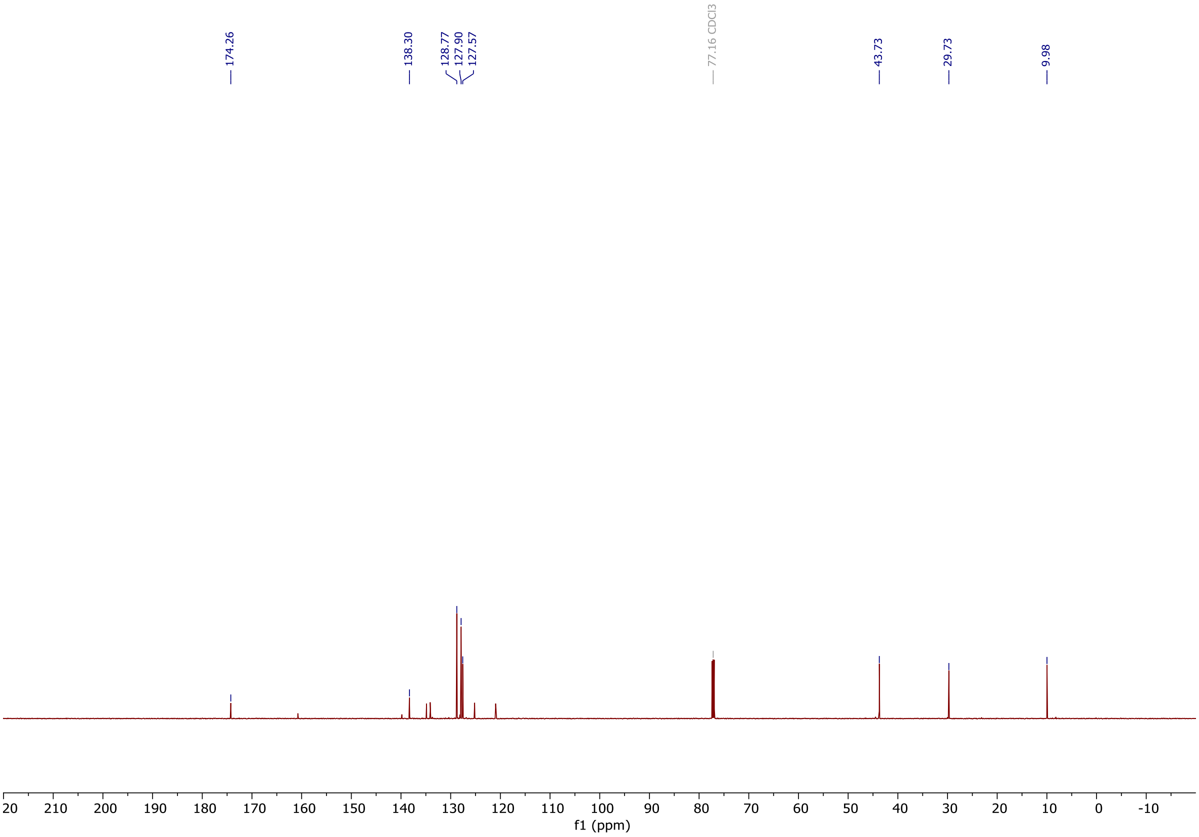

N-methyl-*N*-phenylpropionamide (**5a22**)

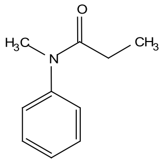

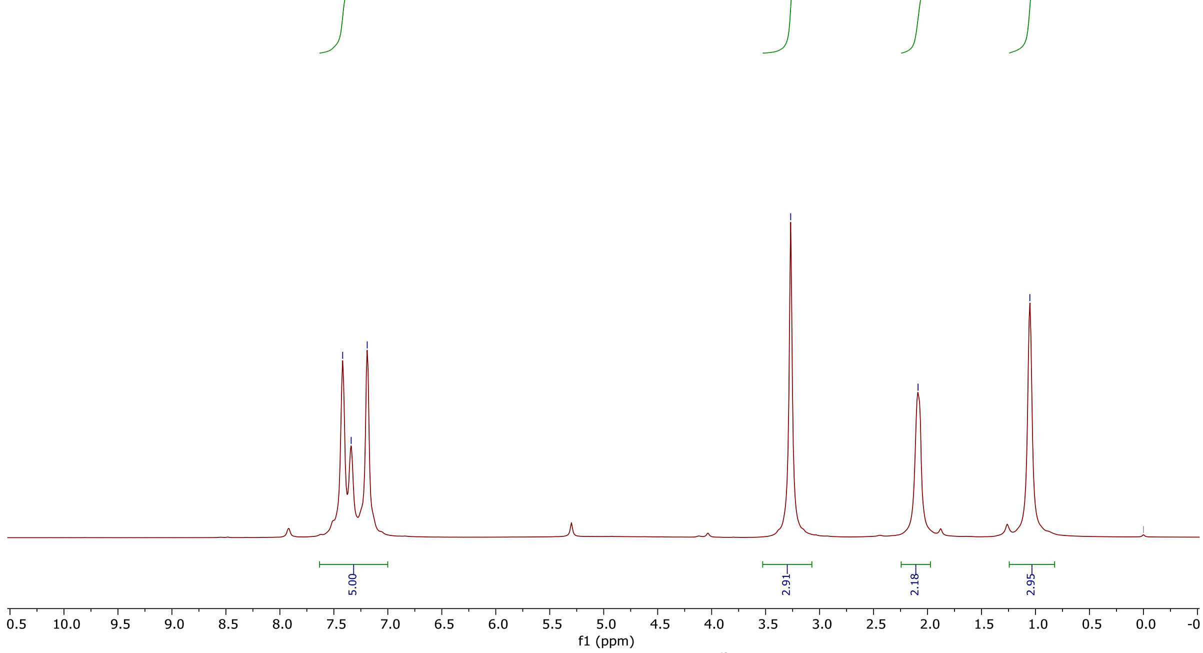

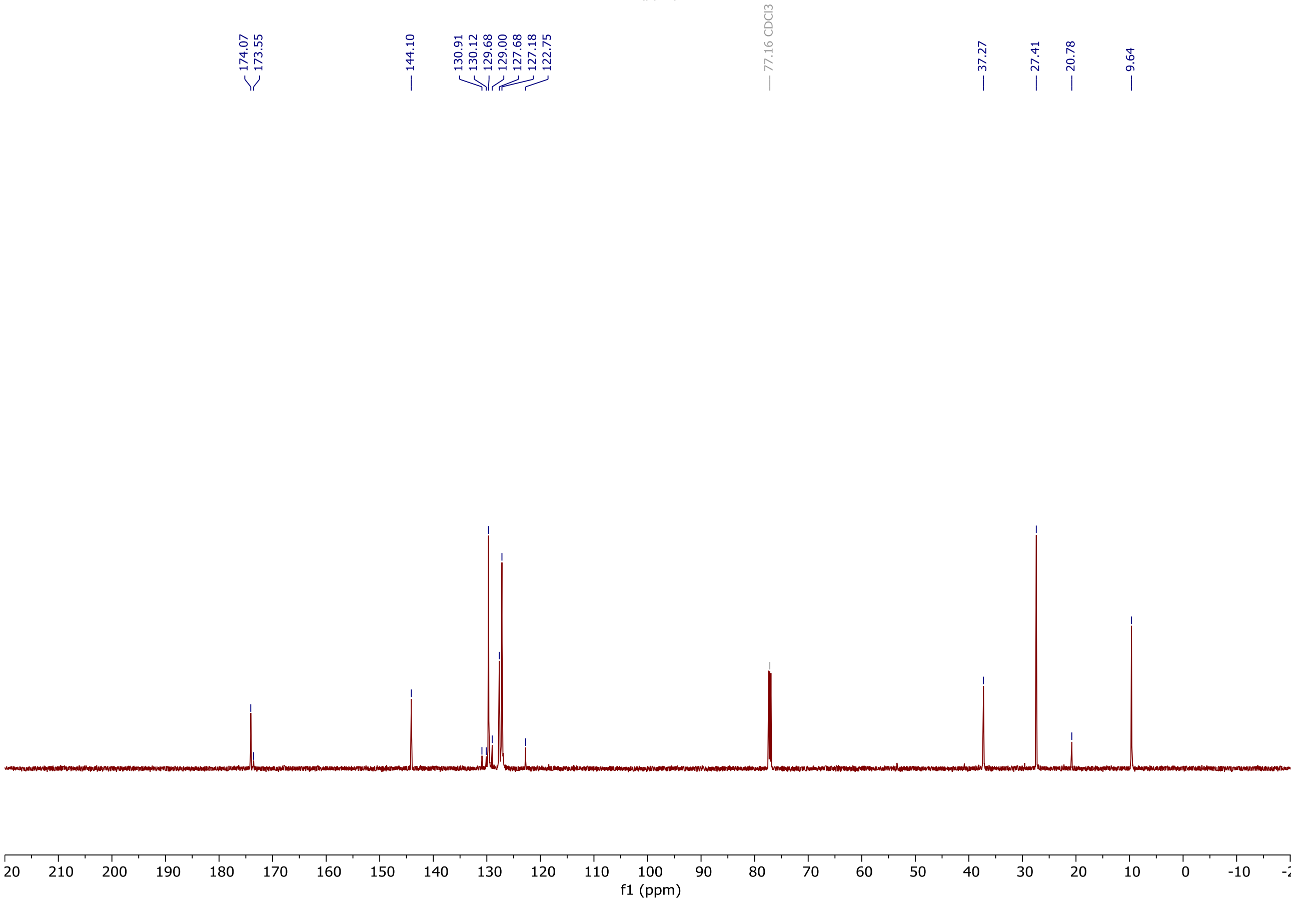

N-allyl-*N*-methylpropionamide (**5a**30)

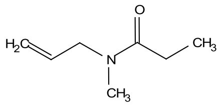

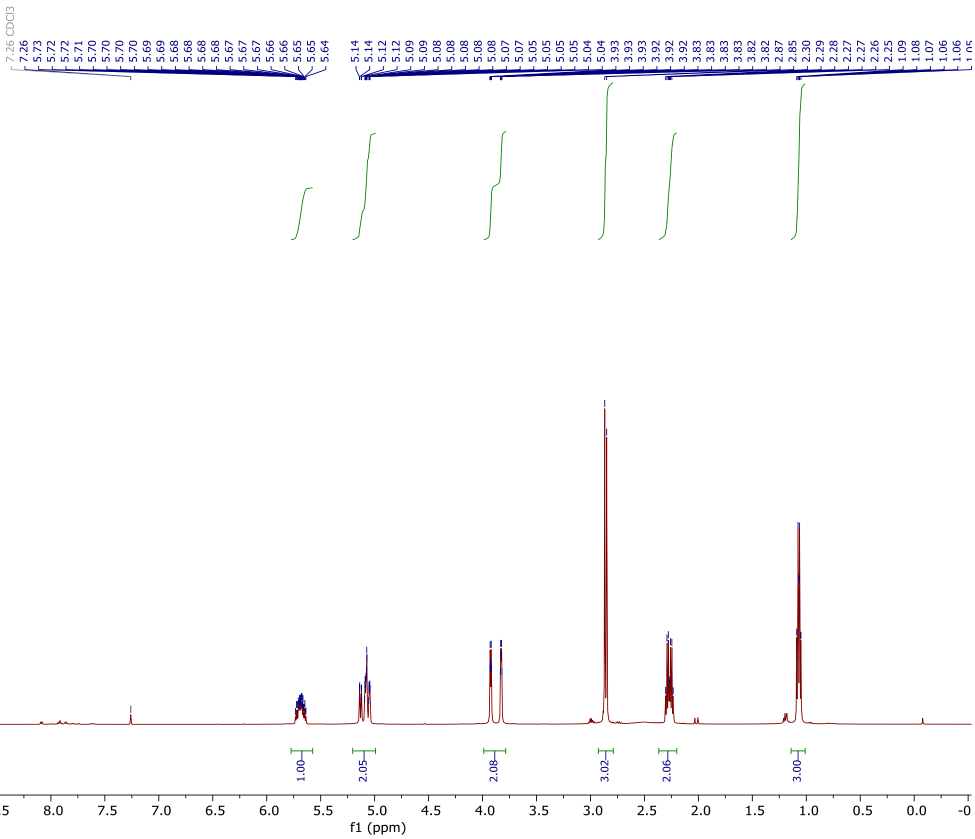

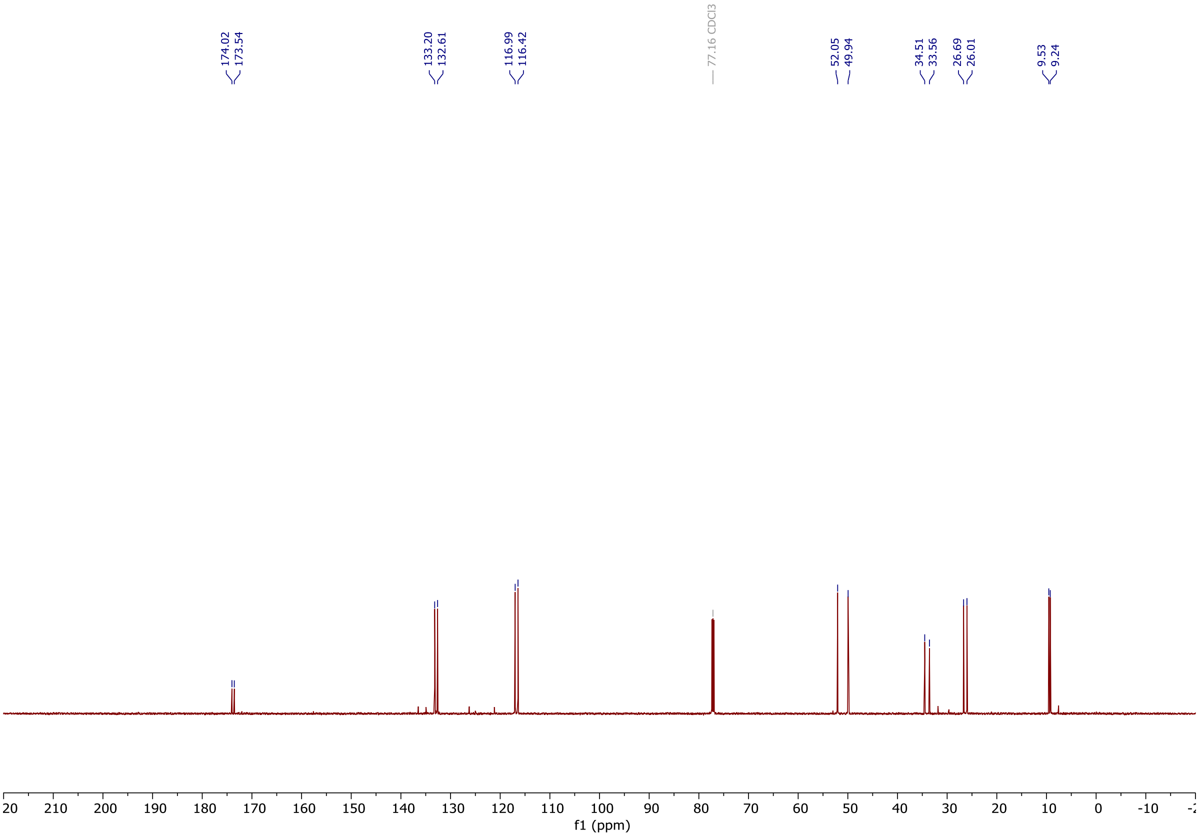

Supplement: Supplementary file 1 [file molecules-27-05450-s001.zip › molecules-1843403-supplementary.pdf]
